# Attrition, physical integrity and insecticidal activity of long-lasting insecticidal nets in sub-Saharan Africa and modelling of their impact on vectorial capacity

**DOI:** 10.1186/s12936-020-03383-6

**Published:** 2020-08-28

**Authors:** Olivier Briet, Hannah Koenker, Laura Norris, Ryan Wiegand, Jodi Vanden Eng, Alex Thackeray, John Williamson, John E. Gimnig, Filomeno Fortes, Martin Akogbeto, Anges W. Yadouleton, Maurice Ombok, M. Nabie Bayoh, Themba Mzilahowa, Ana Paula Abílio, Samuel Mabunda, Nelson Cuamba, Elhadji Diouf, Lassana Konaté, Busiku Hamainza, Cecilia Katebe-Sakala, Gabriel Ponce de León, Kwame Asamoa, Adam Wolkon, Stephen C. Smith, Isabel Swamidoss, Mike Green, Salam Gueye, Jules Mihigo, Juliette Morgan, Ellen Dotson, Allen S. Craig, Kathrine R. Tan, Robert A. Wirtz, Tom Smith

**Affiliations:** 1 Swiss Tropical and Public Health Institute, 4051 Basel, Switzerland; 2 University of Basel, 4001 Basel, Switzerland; 3 PMI VectorWorks, JHU Center for Communication Programs, Baltimore, MD USA; 4 Tropical Health LLP, Baltimore, MD USA; 5 U.S. President’s Malaria Initiative, U.S. Agency for International Development, Washington, DC USA; 6 Present Address: Bill & Melinda Gates Foundation, Seattle, WA USA; 7 Division of Parasitic Diseases and Malaria, Centers for Disease Control (CDC) and Prevention, Atlanta, GA USA; 8 PMI VectorWorks, Denver, CO USA; 9 National Malaria Control Program (NMCP), Ministry of Health, Luanda, Angola; 10 Institute of Hygiene and Tropical Medicine, NOVA University Lisbon, Lisbon, Portugal; 11 Centre de Recherche Entomologique de Cotonou (CREC), Cotonou, Bénin; 12 Programme National de Lutte contre le Paludisme (PNLP), Ministry of Health, Cotonou, Bénin; 13 Kenya Medical Research Institute (KEMRI), Kisumu, Kenya; 14 Present Address: PMI VectorLink Project, Abt Associates, Lusaka, Zambia; 15 College of Medicine, Malaria Alert Centre, P/Bag 360, Blantyre 3, Malawi; 16 Instituto Nacional de Saúde (INS), Ministério da Saúde, Maputo, Mozambique; 17 National Malaria Control Programme (NMCP), Ministry of Health, Maputo, Mozambique; 18 Faculté des Sciences et Techniques (FST), Université Cheikh Anta Diop (UCAD), Dakar, Senegal; 19 National Malaria Control Centre (NMCC), Lusaka, Zambia; 20 U.S. President’s Malaria Initiative, Division of Parasitic Diseases and Malaria, Centers for Disease Control (CDC) and Prevention, Atlanta, GA USA

**Keywords:** Long-lasting insecticidal nets, Durability monitoring, Vectorial capacity, Malaria

## Abstract

**Background:**

Long-lasting insecticidal nets (LLINs) are the primary malaria prevention and control intervention in many parts of sub-Saharan Africa. While LLINs are expected to last at least 3 years under normal use conditions, they can lose effectiveness because they fall out of use, are discarded, repurposed, physically damaged, or lose insecticidal activity. The contributions of these different interrelated factors to durability of nets and their protection against malaria have been unclear.

**Methods:**

Starting in 2009, LLIN durability studies were conducted in seven countries in Africa over 5 years. WHO-recommended measures of attrition, LLIN use, insecticidal activity, and physical integrity were recorded for eight different net brands. These data were combined with analyses of experimental hut data on feeding inhibition and killing effects of LLINs on both susceptible and pyrethroid resistant malaria vectors to estimate the protection against malaria transmission—in terms of vectorial capacity (VC)—provided by each net cohort over time. Impact on VC was then compared in hypothetical scenarios where one durability outcome measure was set at the best possible level while keeping the others at the observed levels.

**Results:**

There was more variability in decay of protection over time by country than by net brand for three measures of durability (ratios of variance components 4.6, 4.4, and 1.8 times for LLIN survival, use, and integrity, respectively). In some countries, LLIN attrition was slow, but use declined rapidly. Non-use of LLINs generally had more effect on LLIN impact on VC than did attrition, hole formation, or insecticide loss.

**Conclusions:**

There is much more variation in LLIN durability among countries than among net brands. Low levels of use may have a larger impact on effectiveness than does variation in attrition or LLIN degradation. The estimated entomological effects of chemical decay are relatively small, with physical decay probably more important as a driver of attrition and non-use than as a direct cause of loss of effect. Efforts to maximize LLIN impact in operational settings should focus on increasing LLIN usage, including through improvements in LLIN physical integrity. Further research is needed to understand household decisions related to LLIN use, including the influence of net durability and the presence of other nets in the household.

## Background

Insecticide-treated bed-nets (ITNs) have repeatedly been shown to reduce morbidity and mortality due to malaria in children [1]. A large part of malaria reduction from 2000 to 2015 has been attributed to their widespread distribution and use [2]. As long-lasting insecticidal nets (LLINs) are expected to retain their biological activity for at least 3 years of recommended use under field conditions according to the World Health Organization (WHO) definition, national malaria control programmes have designed distribution systems on a 3-year cycle. However, LLINs lose their effectiveness against malaria transmission over time from the moment of distribution by accumulating holes as well as through loss of insecticide. Furthermore, many LLINs fall out of use for a variety of reasons before 3 years are over. Recent studies in several countries have suggested that the decline in physical integrity of LLINs exceeds that of insecticidal activity [3–8]. Declining physical integrity is a concern not only because mosquitoes are more likely to enter holed or torn LLINs and feed on the occupants, but also because LLIN owners stop using, discard, or repurpose holed nets, which then no longer contribute to malaria transmission control.

Given that LLINs are the primary prevention and control strategy for much of sub-Saharan Africa, the WHO developed guidelines and recommendations for monitoring three elements of LLIN durability—attrition (also expressed as 1-survivorship), fabric integrity, and insecticidal activity—to guide programmes in designing distribution strategies and enable informed selection of LLIN brands for each country [9]. Beginning in 2009, the US President’s Malaria Initiative (PMI) funded operational research studies in three countries and routine monitoring in four additional countries. The operational research studies directly compared multiple LLINs existing at the time whereas the routine monitoring activities followed LLIN brands distributed through mass campaigns as part of national programme implementation. Some of these activities have published reports while others are being finalized [10, 11].

This analysis extends these individual studies and monitoring activities by exploring large-scale trends using a pooled dataset and exploiting the larger sample size, information on geographical variation, and differences between net brands in durability. These studies monitored the physical integrity (holes), insecticide content, bio-efficacy, use, and attrition of LLINs over time. This analysis combined this monitoring information with experimental hut data to estimate the effect on the vectorial capacity (VC) of LLIN cohorts from distribution until the end of the follow-up. The relative contributions of these different factors to the decay in effect on the VC were computed by comparing the estimated effects on the VC in the real LLIN cohorts with those in counterfactuals in which one of these factors was kept at the best level while the other factors decayed as observed. In principle, such analyses might be relevant for deciding LLIN distribution strategies and frequencies, which are the LLINs with the longest (or shortest) duration of effect, whether purchasing decisions should prioritize physical or chemical durability, and which aspect should be the focus of behavioural change communication (BCC). The results should also inform LLIN manufacturers aiming to develop novel, more durable LLINs.

## Methods

### Study sites and general methodology

This analysis included studies on LLIN durability conducted in seven countries (Table 1) over 5 years. All of the studies monitored one or more factors of LLIN decay including attrition, use, physical integrity and insecticidal activity; however, the design and implementation varied by country (Tables 1 and 2). All studies were prospective, collecting data on selected study LLINs, although the study in Benin included a retrospective component. In all studies, the units of observation were individual nets which allowed for follow up from distribution until loss; the presence and role of non-study nets in the households was not systematically assessed. In Angola and Benin, only a single LLIN brand was evaluated in multiple sites within the country. In contrast, in Mozambique and Zambia, different products were each tested in different sites. In four of the countries, LLINs were monitored as part of routine distribution while in Kenya, Malawi and Senegal, multiple LLIN brands were distributed in a single site, usually comprised of multiple villages, to obtain direct comparisons of different LLIN brands under similar conditions. Each monitoring programme assessed the attrition, physical integrity and insecticidal activity of the LLINs at 6 or 12-month intervals. At each assessment visit, a questionnaire was administered to determine the presence or absence of the LLINs and, if absent, to determine the reason the LLIN was lost. Some questionnaires also assessed LLIN use and washing, and in some studies other factors potentially related to LLIN durability (incl. house type, number of residents, presence of animals) were surveyed. Physical integrity was measured either in the field or in the laboratory using LLINs that were destructively sampled. In most studies, the holes were counted in size categories although in Kenya and Senegal, holes were individually measured. Most studies conducted the WHO cone bioassays with the standard laboratory strain (*Anopheles gambiae* Kisumu strain) although different studies tested different locations on the LLIN. All countries performed chemical analysis by gas chromatography (GC) or high-performance liquid chromatography (HPLC), except for Benin where a colorimetric test was used to measure surface levels of insecticide on most LLINs and HPLC was only used on LLINs with low levels of insecticide.

**Table 1:** Field activities by country

Country	Angola	Benin	Kenya	Malawi	Mozambique	Senegal	Zambia
Years	2011–2014	2011–2014	2009–2014	2009–2013	2008–2011	2010–2013	2012–2014
Sites	4 municipalities in 2 provinces (Kwanza Sul and Uije)	6 sites	Gem District	Chikhwawa District	6 sites in Nampula Province (Malema, Chiulo, Ribaue, Quinga, Chinga, Angoche)	5–6 villages, depending on the number of LLINs available	4 districts in Luapula Province and 4 districts in Northern Province
Target nets distributed	2671 nets	500 per site (2 sites for retrospective, 4 sites for prospective)	4505 (attrition)	3120 (attrition)	6000 were barcoded, 2023 were found in mapping exercise	600 per village	500 each brand, distributed Feb-June 2011 (nets not distributed as part of study)
Frequency of Follow up	Annual	Every 6 months	Every 6 months	Every 6 months	Annually	Every 6 months	Every 6 months
Mean Households interviewed at each follow up (range)	230 (153–307)	1426 (978–1821)	3745 (2962–4383)	2369 (1948–2693)	214 (198–232)	1919 (747–3151)	815 (545–999)
Average Percentage of nets examined for holes (%)	48.9	51.0	13.1^a^	7.5^a^	69.8	69.7	78.7
Assignment of LLIN brands to sites	Not random	Not random	Randomly assigned to several villages (at least two villages per brand)	Olyset Net was tested in one area only. Other LLIN brands distributed randomly	Not random	5 LLIN brands tested concurrently in 17 villages	Not random
Study design	Repeated cross-sections of 50 households per district once per year	Retrospective 2-year-post (2007–2008). Prospective with visits every 6-monthly (2011 on)	Prospective, with surveys every 6 months	Prospective, with surveys every 6 months	Repeated cross-sections of 30 LLINs collected per site once per year	Prospective, with surveys every 6 months	Prospective (each Peace Corps Volunteer followed 25 LLINs for length of study with surveys every 6 months)
Quantification of hole size	Measurement of long axis to the nearest centimeter; holes < 0.5 cm recorded as < 0.5 cm. 0.5–1 cm recorded as 1 cm	Categories: (I—smaller than a coin); (II—between coin and hand size); (III—larger than a hand);	Length at widest point; distance from bottom of the LLIN	Small (< 2 cm rod), medium (> 2 cm rod, < 9 cm rod), large (> 9 cm rod)	Measurement of long axis to the nearest centimeter; for 0.5–1 cm holes record as 1 cm, for < 0.5 cm record as < 0.5 cm	Counted by size (< 5 cm vs > 5 cm) and placement (top, side, or bottom)	Classified relative to thumb size (0.5–2 cm)
Laboratory hole counting	Yes	No	Yes	Yes	Yes	Yes	No

^a^In both Kenya and Malawi 30 randomly sampled nets were selected for hole counting and chemistry every 6 months for first 2.5 years; in later surveys 50 were selected

**Table 2:** Number of LLINs included in the analysis of attrition and use

Country	Dawa-plus 2.0	DuraNet	Inter-ceptor	LifeNet	Net-Protect	Olyset Net	Perma-Net 2.0	Perma-Net 3.0	Total
Angola^a^	186								186
Benin^b^						2002			2002
Kenya^b^	592	772	687		638	563	521	610	4383
Malawi^b^		527	535		577	528	518		2685
Mozam-bique^a^						66	125		191
Senegal^b^	474			361	442	666	477		2420
Zambia^b^						500	499		999
Total	1252	1299	1222	361	1657	4325	2140	610	12,866

^a^The tabulated numbers for these countries are the average numbers of LLINs included in each cross-sectional evaluation

^b^The tabulated numbers for these countries (where LLIN cohorts were analysed) are the numbers of LLINs included in the first follow-up. A few additional non-cohort LLINs were included at subsequent time points in the analysis of overall LLIN use

### Attrition

Attrition of LLINs, the process whereby the cohort of LLINs is reduced over time, was assessed by comparing LLIN cohorts in successive surveys. LLINs identified as missing with a reason of ‘destroyed’, or repurposed (e.g. for fishing) were considered to have been eliminated, while LLINs identified as missing for other reasons (e.g. stolen, given away or lost) were considered missing data (lost to follow up) as stolen or lost LLINs could still be in use elsewhere.

Studies in Angola and Mozambique deployed repeated cross-sectional sampling, and the survival function was calculated as the number of LLINs present, divided by the number of LLINs present plus the number of LLINs destroyed or repurposed. Studies in Benin, Kenya, Malawi, Senegal, and Zambia, followed LLINs longitudinally. Kaplan–Meier estimates were used to estimate attrition by age of LLIN. A logistic regression Bayesian approach was used to estimate the survival function separately for each site and LLIN brand, allowing for missing data when the status was unclear such as when a LLIN was not observed in a survey and/or the household was not available at the time of the survey (see Additional file 1).

### LLIN use

In studies with a repeated cross-sectional design (Angola and Mozambique), LLIN use, conditional on LLINs being present, was calculated as the number of LLINs reported to have been used, divided by the number of LLINs present. For each site, LLIN brand, and time point, the proportion of all distributed LLINs in use was calculated by multiplying the proportion in use conditional on presence by the proportion still present up to that time (from the attrition analysis). These proportions were jointly estimated in a Bayesian hierarchical model.

In the longitudinal studies in Kenya, Malawi, Senegal and Zambia, the same LLIN was observed on one or more follow up visits. Accordingly, LLIN use conditional on a LLIN being present was analysed with LLIN-level random effects, accounting for non-independence of observations (using a Bayesian model described in Additional file 1). In the Benin study, information on use was elicited only when collecting sampled LLINs, so each LLIN was observed only once.

### Physical integrity of LLINs

Where available, data on the numbers of holes in LLINs were converted to total holed area (in cm^2^) using published conversion factors for elliptical holes [12]. The distribution of hole sizes in the whole LLIN cohort was estimated by fitting normal distributions to the log_e_(1 + holed area), with the mean and variance varying by use-status, LLIN brand and country. LLINs that had additional unquantified damage (e.g. when seam failures were recorded or where parts of panels of LLINs were missing) were treated as right-censored observations. Example code for this analysis is provided in Additional file 1.

### Insecticide content and insecticidal effects

Standard HPLC and GC methods were used to measure the active ingredient content of sampled LLINs from a subset of the surveys. Cone bioassays [13] were applied to estimate the corresponding insecticidal effects. For each LLIN brand in each country, logistic regression models were fitted separately, relating the proportion of mosquitoes dead in the cone bioassays to the logarithmically transformed active ingredient content. These models were used to standardize active ingredient content by translating them onto a scale of lethality in cone bioassays, and mapping this onto the corresponding equivalent for a standard LLIN brand (PermaNet 2.0) (for details see Additional file 2). The models were again fitted using a Bayesian Markov chain Monte Carlo (MCMC) algorithm. This provided point and interval estimates for the factors scaling the active ingredient content to the standard, for each LLIN brand and for each country (Kenya, Malawi, Mozambique, Senegal and Zambia) that provided bio-assay data for multiple LLIN brands including PermaNet 2.0. In a further analysis the data from all countries were combined to provide a single scaling factor for each LLIN brand (Additional file 2).

### Impact on vectorial capacity

A discrete-time mathematical model of malaria in mosquitoes [14] was used to make predictions of the impact of a LLIN on VC of the mosquito population as a function of the standardized active ingredient content, and the holed area of the LLIN. Previously published parameters were used to obtain estimates of VC from a model of the feeding cycle for *Anopheles gambiae* sensu stricto (s.s.) in the absence of intervention [15, 16]. This model captures the reduction in VC by LLINs as increases in mortality at different stages of the feeding cycle and extension of the duration of the cycle as a result of deterrence. These effects were parameterized with data from multiple experimental hut studies. The values for the effects on the probability that a mosquito enters a hut ($P_{ent}$) were derived from a study of effects of washing LLINs on hut entry [17]. Data from mosquito release–recapture assays in huts in Benin [18] were used to estimate how the probabilities of attacking ($P_{att}$), of pre-prandial ($P_{B\mu }$), and of post-prandial killing ($P_{C\mu }$) for both susceptible *An. gambiae s.s.* Kisumu strain and resistant *An. gambiae* sensu lato (s.l.) (Akron) depend on insecticide content and holed surface area in PermaNet 2.0 nets. This analysis led to a series of logistic models for each of these parameters as functions of the holed surface area, *H,* in cm^2^, and the insecticide, X, of content [X] in mg/m^2^:

$$\begin{aligned} \log {\text{it}}\left( {P_{ent} \left( X \right)} \right) & = \beta_{0,ent} + \beta_{1,ent} \gamma \ln \left[ {X + 1} \right] \\ \log {\text{it}}\left( {P_{att} \left( {X,H} \right)} \right) & = \beta_{0,att} + \beta_{1,att} \ln \left( {H + 1} \right) + \beta_{2,att} \gamma \ln \left[ {\left( X \right) + 1} \right] + \beta_{3,att} \gamma \ln \left[ {\left( X \right) + 1} \right]\ln \left( {H + 1} \right) \\ \log {\text{it}}\left( {P_{B\mu } \left( {X,H} \right)} \right) & = \beta_{0,B\mu } + \beta_{1,B\mu } \ln \left( {H + 1} \right) + \beta_{2,B\mu } \gamma \ln \left[ {\left( X \right) + 1} \right] + \beta_{3,B\mu } \gamma \ln \left[ {\left( X \right) + 1} \right]\ln \left( {H + 1} \right) \\ \log {\text{it}}\left( {P_{C\mu } \left( {X,H} \right)} \right) & = \beta_{0,C\mu } + \beta_{1,C\mu } \ln \left( {H + 1} \right) + \beta_{2,C\mu } \gamma \ln \left[ {\left( X \right) + 1} \right] + \beta_{3,C\mu } \gamma \ln \left[ {\left( X \right) + 1} \right]\ln \left( {H + 1} \right) \\ \end{aligned}$$

where $\gamma$ is the factor used for scaling the insecticide content for the different LLIN brands against PermaNet 2.0 (see Additional files 1, 2, 3). $H_{max}$ is substituted for *H* to calculate the values for an unprotected human, and deterrence is captured by the effects on the availability of LLIN users to mosquitoes, relative to non-users:

$$\frac{\alpha }{\alpha \left( 0 \right)} = \frac{{P_{att} \left( {X, H} \right)P_{ent} \left( X \right)}}{{P_{att} \left( {0, H_{max} } \right)P_{ent} \left( 0 \right)}}$$

Further details, including the parameter estimates, are provided in Additional file 2, and in the appendix to the analysis of the original hut trials from Benin [18].

**Fig. 1:**
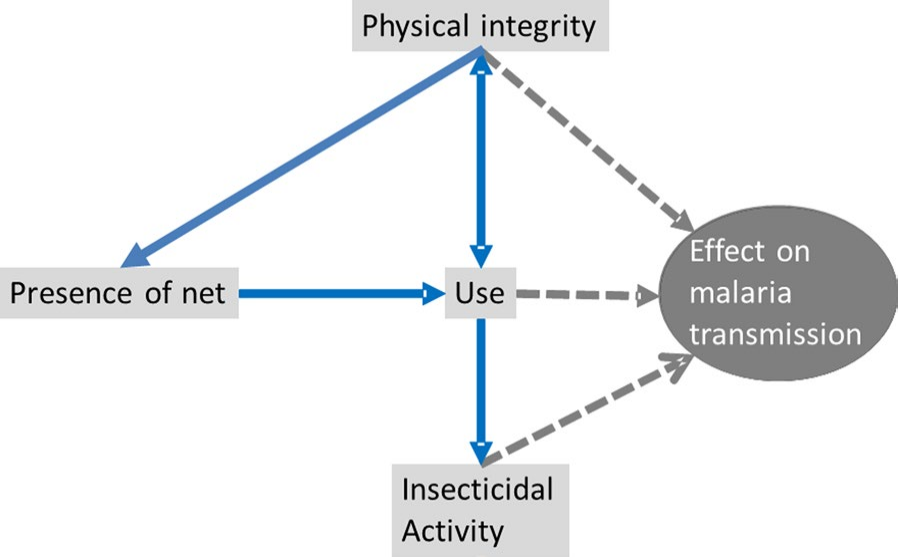
Causal diagram for factors determining the effects of LLINs on malaria transmission. Solid lines indicate the main causal relationships between the measured quantities; dashed lines indicate which factors impact malaria transmission (via relationships estimated from experimental hut data and captured in the mathematical model)

Estimates of the impact of LLINs on VC over time used the estimates of $P_{ent}$, $P_{att}$, $P_{B\mu }$ and $P_{C\mu }$ for the specific case where (1) enough LLINs were distributed to provide 100% (general) population access, (2) access scaled linearly with LLIN survival and use, and (3) no other LLINs were present in the population. A schematic of the analytical approach is provided in Fig. 1.

In order to estimate the importance of attrition, non-use, physical decay, and chemical decay, the effect on VC was calculated for a series of counterfactual scenarios in which different components of net decay (attrition, use, insecticide decay and decay of physical integrity) were precluded in turn, while keeping other properties as in the field surveys. Thus, in the counterfactuals, interactions between the components were not taken into account.

### Software

Where practicable, Bayesian models were used to fit statistical models to the data using MCMC algorithms to estimate each average quantity by LLIN brand and survey. This made it possible to propagate the statistical uncertainty at each stage through the analyses, allowing for variation in the physical and chemical status of LLINs. Models were fitted using the software JAGS [19], called from the software platform R (version 3.6.2) [20]. Details are provided in Additional files 1, 2, 3. The variance component analysis was carried out using a restricted maximum likelihood (REML) algorithm in the R variance component analysis (VCA) package. The mathematical model of Chitnis et al. [14] was implemented in R as described previously [15].

## Results

**Fig. 2:**
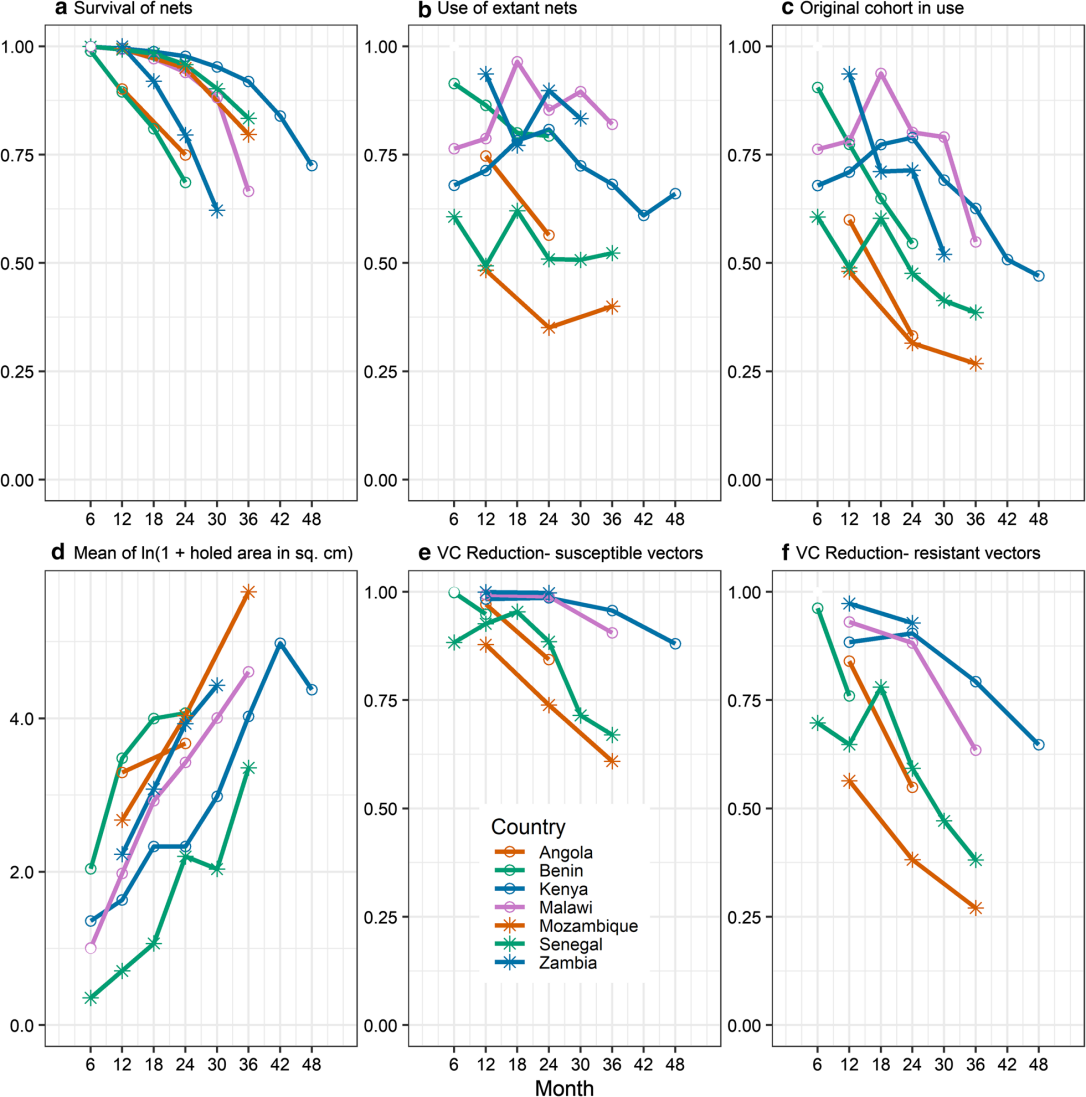
Survival (1-attrition) (**a**), use of nets currently in the household (**b**), proportion of original cohort of nets in use (**c**), mean of the natural log of estimated hole area (**d**), reduction in vectorial capacity of pyrethroid susceptible mosquitoes (**e**), and reduction in vectorial capacity of pyrethroid resistant mosquitoes (**f**), by country

**Fig. 3:**
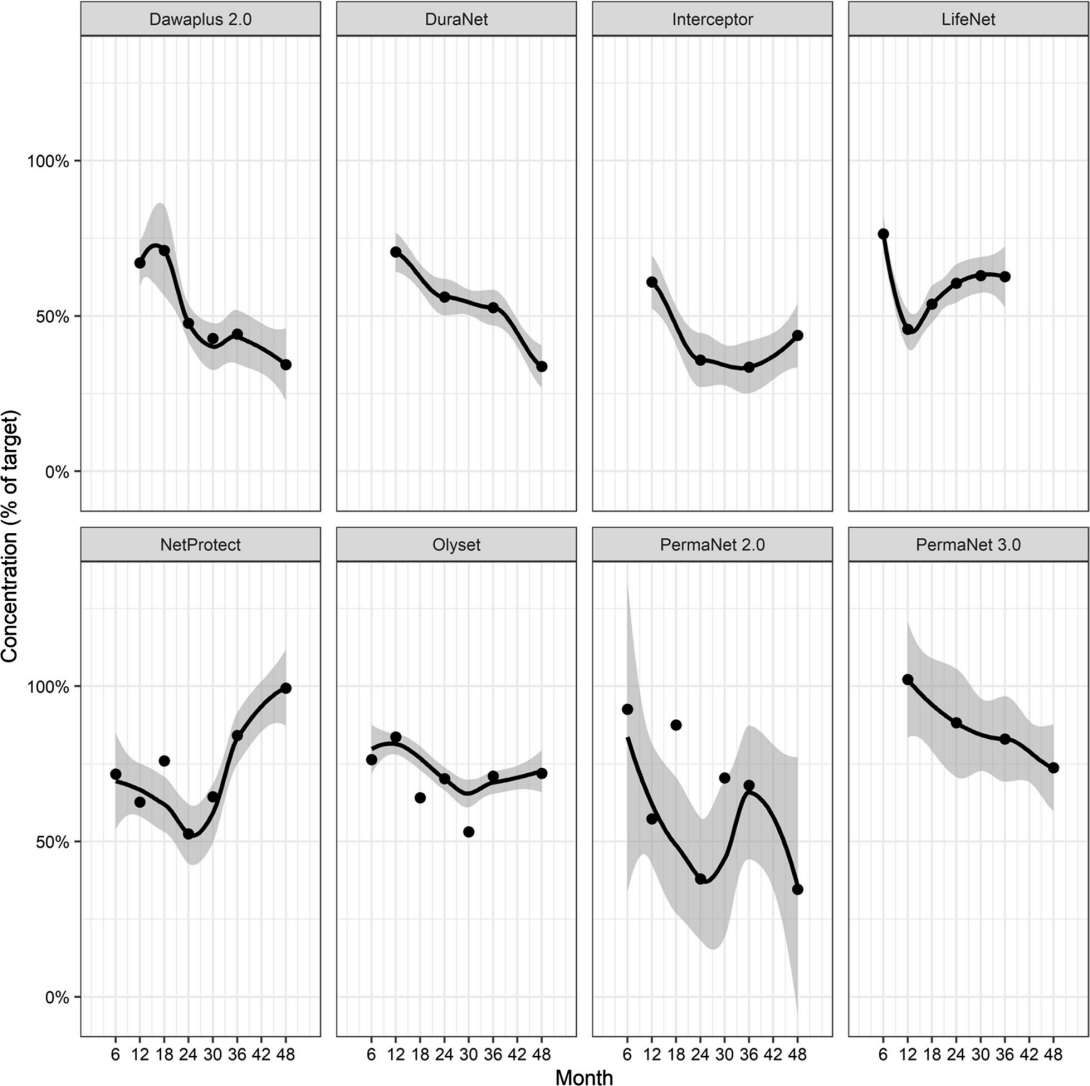
Persistence of active ingredients by LLIN brand

Temporal decay is evident for each factor considered in the study, but the patterns of attrition, use, physical decay, and loss of insecticide from LLINs are very different (Figs. 2 and 3, and Additional file 2: Figs. S1–S5).

Average survivorship (1-attrition) of LLINs by country follows convex curves (Fig. 2a), in contrast to exponential decays (which would be concave). Country-specific variation in average attrition is evident, with the most rapid attrition in Benin, and the slowest in Kenya (Table 3). Patterns of attrition varied more strongly depending on the country than depending on the LLIN brand, and LLINs of the same brand varied between countries (see Additional file 2: Fig. S1). This is confirmed by variance component analysis (Table 4), which indicated that the proportion of the variation in attrition due to differences due to country was 4.6 times more important than differences due to net brand.

**Table 3:** Average status at 24 months

Country	Survival of LLINs	Proportion of extant LLINs being used	Proportion of original LLIN cohort being used	Mean of ln(holed area in sq. cm)	VC Reduction -resistant mosquitoes	VC Reduction -sensitive mosquitoes
Angola	0.749 (0.034)	0.564 (0.045)	0.332 (0.038)	3.673 (0.239)	0.549 (0.047)	0.844 (0.032)
Benin	0.686 (0.014)	0.793 (0.031)	0.545 (0.024)	4.069 (0.187)	–	–
Kenya	0.977 (0.003)	0.808 (0.007)	0.790 (0.007)	2.329 (0.183)	0.904 (0.005)	0.987 (0.002)
Malawi	0.940 (0.005)	0.853 (0.009)	0.801 (0.010)	3.427 (0.235)	0.882 (0.006)	0.989 (0.001)
Mozambique	0.950 (0.015)	0.351 (0.037)	0.315 (0.034)	4.037 (0.321)	0.382 (0.048)	0.739 (0.040)
Senegal	0.958 (0.005)	0.509 (0.016)	0.476 (0.016)	2.200 (0.098)	0.592 (0.015)	0.885 (0.011)
Zambia	0.795 (0.015)	0.898 (0.015)	0.713 (0.018)	3.930 (0.092)	0.928 (0.007)	0.997 (0.001)

Tabulated values are averages across all LLIN brands weighted in proportion to their representation in the cohorts (Table 2). Values in parentheses are standard errors of the means. ln() = natural logarithmic transformation

**Table 4:** Variance component analysis of differences between countries, LLIN brands and survey periods in factors contributing to durability

	Source of variation				
	Country	LLIN brand	Time period	Country: time period interaction	LLIN brand: time period interaction
Survival of LLINs^a^					
σ^2^	1.1 (0*–3.2)	0.2 (0*–0.6)	2.8 (0*–6.7)	2.2 (0.6–3.8)	0.4 (0*–0.8)
%Total	14.6	3.2	36.0	27.9	5.2
Use of extant LLINs^a^					
σ^2^	15.6 (0*–35.8)	3.6 (0*–8.3)	0.5 (0*–2.4)	1.4 (0*–4.2)	0.0 (0*–0.0)
%Total	47.3	10.8	1.4	4.3	0*
Original cohort LLINs in use^a^					
σ^2^	10.7 (0*–25.2)	3.2 (0*–7.4)	6.6 (0*–15.2)	2.0 (0*–5.1)	0* (0.0–0.0)
%Total	33.0	9.8	20.3	6.2	0*
Mean of Logarithm of holed area					
σ^2^	0.7 (0*–1.6)	0.4 (0*–0.9)	1.7 (0*–3.6)	0.0 (0*–0.1)	0.0 (0*–0.1)
%Total	21.5	12.3	52.6	1.1	1

σ^2^: Estimate of variance component (95% confidence intervals); %Total is the percentage of the total variance in the outcome

*The REML algorithm constrains the estimates of variance components to be positive. Values of zero are substituted where the algorithm did not converge on a positive value

^a^Proportions analysed on a logit scale

LLIN use was assessed both as the proportion of LLINs used the last night out of LLINs present in the household (extant nets), and as the proportion of LLINs used the last night out of the original LLIN cohort, thus taking attrition into account. In contrast to patterns of attrition, patterns of use of the LLINs over time (Fig. 2b) did not show any clear overall temporal pattern, and the ranking of countries is different, with Malawi and Zambia showing high use, and Mozambique the lowest (Table 3). As with attrition, differences in overall use between LLIN brands were less clear than were the differences between countries (see Additional file 2: Figure S2 and analysis) and variance component analysis (Table 4), shows that the proportion of the variation in use due to differences due to country was 4.4 times greater than differences due to net brand.

The specific LLIN brands that were in use did not show clear relationships with the corresponding rates of attrition. Interceptor nets (polyester, coated nets) had the highest average use, and LifeNet the lowest (polypropylene, incorporated nets), but the ranking of LLIN brands by use differed by country. There is no obvious explanation for the patterns. However, in most cases there was a clear decrease with age of LLIN in the proportion of the original cohort of LLINs that were used (Fig. 2c and Additional file 2: Fig. S3 and analysis). In some countries, such as Malawi, there was an initial increase in use of cohort LLINs over time, which could arise if LLINs from an earlier distribution were available, and the new LLINs only came into use as the previously distributed LLINs were subject to attrition.

The holed area of those cohort LLINs in use varied considerably between country, but in each country increased over time approximately exponentially for the first 3 years (i.e. approximately following straight lines in Fig. 2d). In the follow-up from 42 to 48 months in Kenya (the only country with 4-year follow-up) there was no further increase in average holed area. Within countries, different net brands generally had similar patterns of physical decay (see Additional file 2: Fig. S4 and analysis). As with LLIN attrition and LLIN use, variance component analysis (Table 4) indicated that the proportion of the variation in holed area due to differences due to country was larger than differences due to net brand, although only 1.8 times larger.

In contrast to this evidence for increasing rates of physical damage over time, there was generally a slower decline in the measured levels of the active insecticidal ingredients (Fig. 3 and Additional file 2: Fig. S5), with rather stable differences between LLIN brands in average content of active ingredients, indicating chemical stability. Four of the net products had average insecticide content > 50% of the target dose after 3 years and none had lost more than 75% of their target dose. The differences in average content between LLIN brands are largely a result of the different active agents (Table 5), but the relationships between the proportion of dead mosquitoes in cone bioassays with the insecticide content varied considerably among countries for the same LLIN brand, with regression coefficients for e.g. PermaNet 2.0 varying between 1.34 and 2.70, a two-fold difference (Additional file 2). However, the ratio between the coefficients of specific LLIN brands and that of PermaNet 2.0 was more reproducible across country studies, with at maximum a 1.38-fold difference. This would suggest much of the mortality effects are user dependent and may reflect differences in the mosquito strains tested, differences in their rearing practices or differences in the performance of the bioassay (i.e. people may be less gentle with the mosquitoes when transferring from cone to paper cup or insectary conditions such as temperature or humidity may affect mortality) but these differences were consistent by LLIN brand within the same country/lab. Since the differences between countries in bioassay results are thus likely to be an artefact, this outcome was not included in the variance component analysis (Table 4).

**Table 5:** LLIN target insecticide content levels and scaling factors

LLIN brand	Insecticide	Target active agent content (mg/m^2^)	Scaling factor ($\gamma$) (95% CI)
DawaPlus 2.0	Deltamethrin	80	1.00 (0.98–1.01)
DuraNet	Alphacypermethrin	261	1.04 (1.02–1.07)
Interceptor	Alphacypermethrin	200	0.68 (0.67–0.69)
LifeNet	Deltamethrin	340	0.99 (0.94–1.05)
NetProtect	Deltamethrin	68.1	0.83 (0.82–0.84)
Olyset Net	Permethrin	1000	0.35 (0.35–0.36)
*PermaNet 2.0	Deltamethrin	55	1
PermaNet 3.0 lower side panels	Deltamethrin	85	2.32 (2.24–2.40)
PermaNet 3.0 upper side panels	Deltamethrin	85	2.09 (2.02–2.16)
PermaNet 3.0 top panel	Deltamethrin and PBO	121	1.02 (0.99–1.05)

* Reference LLIN brand

**Fig. 4:**
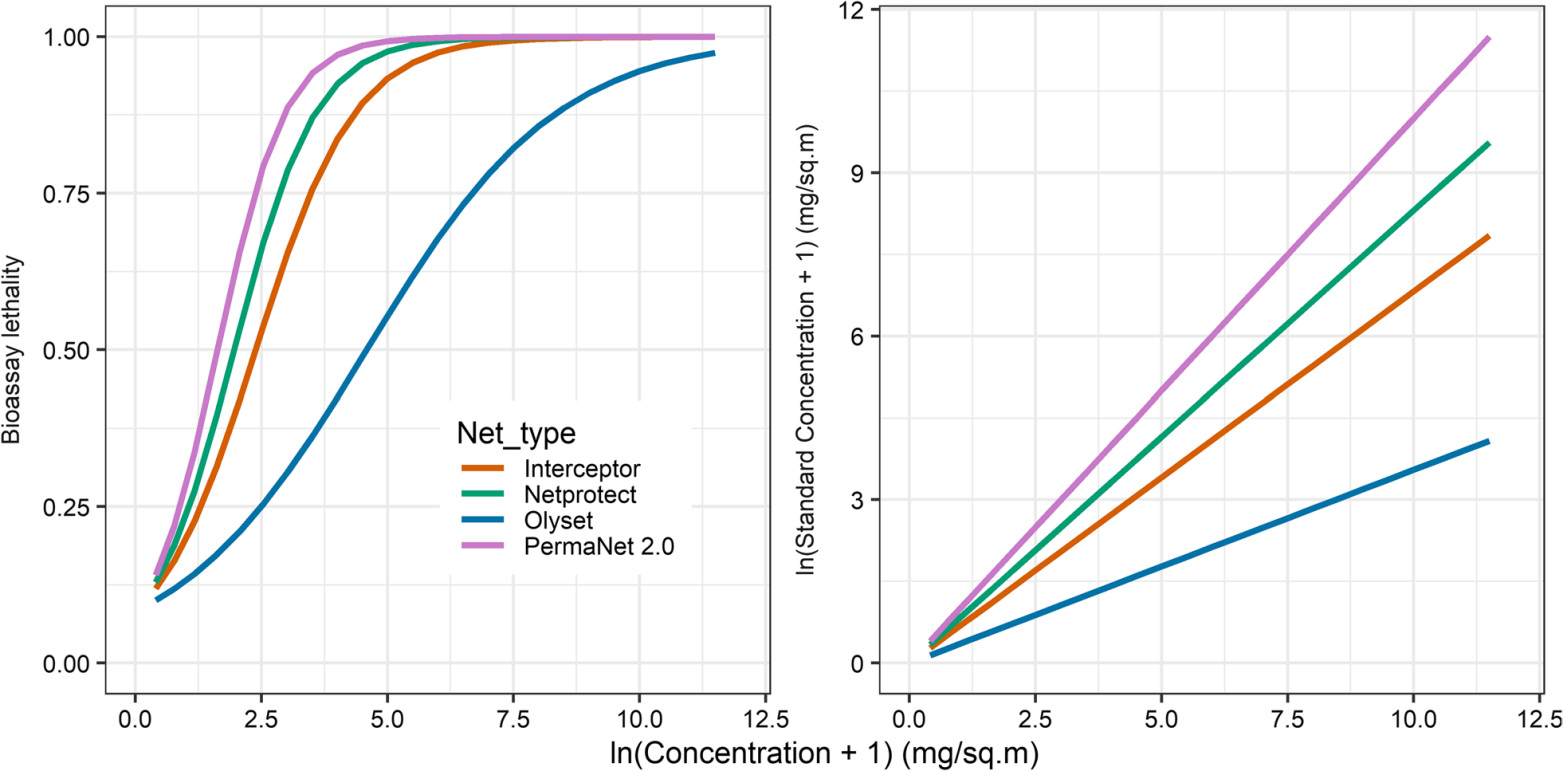
Lethality in cone tests and calibration of active ingredient content. The lethality and calibration curves are shown only for four specific LLIN brands. The lines for the other LLIN brands are very close to that for PermaNet 2.0

The lack of consistency in bioassay results justifies the use of PermaNet 2.0 as a standard for comparison of insecticidal effects of different LLIN brands, and of the straight-line calibration curves (Fig. 4). Differences in the calibration curves may reflect differences in the LLIN material and consequently the release rate of insecticide. For example, PermaNet 2.0 is a coated technology with insecticide held in a resin coating on the fibers; NetProtect was polyethylene where insecticide is incorporated in the fibers and much of the insecticide may be unavailable to contact and kill mosquitoes.

**Fig. 5:**
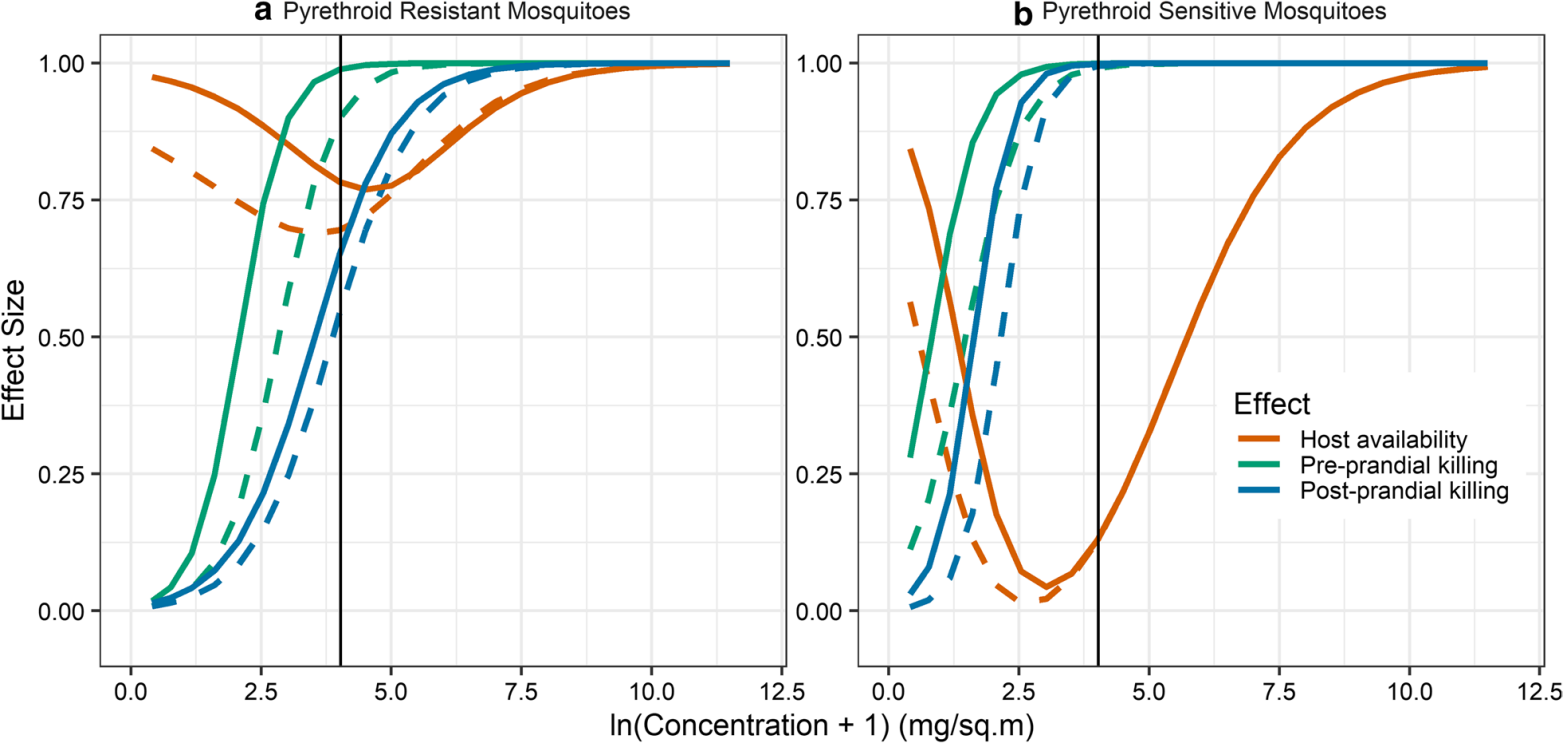
Predicted entomological effects of holed and intact LLINs. **a** Pyrethroid resistant mosquitoes. **b** Pyrethroid sensitive mosquitoes. The vertical black line corresponds to the target active agent content for PermaNet 2.0. The continuous lines correspond to intact LLINs and the dashed lines to LLINs with a holed area of 50 cm^2^. The effect size, on the vertical axis is the proportion by which availability of humans to mosquitoes is reduced, or killing of mosquitoes increased, when the LLIN is in use

The parameterization of the model for predicting impacts on VC of pyrethroid sensitive *An. gambiae* is described in detail elsewhere [15]. House entry depends on insecticide content (but not on the integrity of the LLINs). Perhaps counterintuitively, resistant mosquitoes are more easily deterred from entry than sensitive mosquitoes (Additional file 2: Fig. S9). The effects on pre-prandial and on post-prandial killing are stronger at lower insecticide content with sensitive mosquitoes compared to resistant mosquitoes (Fig. 5). With sensitive mosquitoes (but not resistant ones) there is saturation of both mortality and deterrent effects well below the target insecticide content for PermaNet 2.0. This is consistent with the effect of resistance being to require more insecticide to achieve the same physiological effect. Holed LLINs are less effective in killing mosquitoes (even in experimental settings where holes and insecticide content are independent), so the curves for holed LLINs are consistently to the right of those for intact LLINs for killing effects, while holes make the human host more available to mosquitoes. At target insecticide content, there is little difference in effects between intact LLINs and LLINs with substantial holed area on sensitive mosquitoes (Fig. 5), but damage to LLINs is more important when the mosquitoes have acquired pyrethroid resistance.

The analysis of counterfactuals suggests that increasing use of LLINs—regardless of their physical or insecticidal condition—would be the most promising way to achieve greater impact in most of the study settings (Table 6 and Table 7). The direct effect of preventing chemical or physical decay accounts for a much smaller proportion of the loss over time in effect on VC. For susceptible mosquitoes, 65–96% of the residual transmission as measured by VC could be prevented by achieving 100% usage of nets. In all countries, the credible intervals did not overlap zero. In contrast, 5–62% of residual VC could be prevented by maximizing physical integrity of nets, − 50–80% could be prevented by maximizing insecticidal activity and − 3–72% could be prevented by minimizing attrition of the LLINs. The credible interval for these estimates overlapped zero in most cases except for physical integrity in Malawi and Zambia, insecticidal activity in Benin and attrition in Angola, Kenya and Malawi. Similar trends in the analysis of counterfactuals were observed for pyrethroid resistant mosquitoes (Table 6). The impact of preventing LLIN insecticide decay was larger than the impact of preventing physical decay for five out of eight LLIN brands. For Olyset Net, DuraNet and PermaNet 3.0, the direct impact of preventing physical decay was generally larger (yet the impact of preventing physical decay was not significantly different from zero for DuraNet and PermaNet 3.0 in any of the studies). However, this analysis does not allow for the indirect effect of either kind of decay on LLIN use or attrition (Fig. 1).

**Table 6:** Proportion of residual vectorial capacity of susceptible *An. gambiae s.l.* prevented by improving LLIN durability properties, by country

Country	LLIN brand	Sem.	Intact scenario	Target content scenario	Maximum use scenario	Maximum survival scenario
Angola	1	2, 4	0.05 (− 0.82 to 0.43)	0.00 (− 0.91 to 0.42)	0.82 (0.62 to 0.91)	0.73 (0.42 to 0.87)
Benin	5	1, 2	0.36 (− 0.29 to 0.66)	0.80 (0.55 to 0.90)	0.65 (0.55 to 0.74)	0.24 ( to 0.39 to 0.53)
Kenya	1, 2, 3, 4, 5, 6, 7	2, 4, 6, 8	0.11 (− 0.01 to 0.22)	− 0.52 (− 0.80 to 0.30)	0.89 (0.86 to 0.90)	0.26 (0.16 to 0.36)
Malawi	2, 3, 4, 5, 6	2, 4, 6	0.29 (0.11 to 0.42)	0.16 (− 0.04 to 0.32)	0.72 (0.67 to 0.77)	0.39 (0.24 to 0.51)
Mozambique	5, 6	2, 4, 6	0.06 (− 0.33 to 0.31)	0.07 (− 0.34 to 0.33)	0.96 (0.94 to 0.97)	0.03 (− 0.41 to 0.25)
Senegal	1, 4, 5, 6, 8	1,2, 3,4, 5, 6	0.13 (− 0.17 to 0.26)	0.09 (− 0.19 to 0.21)	0.92 (0.91 to 0.93)	0.03 (− 0.27 to 0.16)
Zambia	5, 6	2, 4	0.62 (0.23 to 0.79)	0.26 (− 0.24 to 0.52)	0.81 (0.74 to 0.86)	− 0.03 (− 0.62 to 0.31)

Numbers within parentheses are 95% credible intervals. Cells highlighted in green have credible intervals entirely above zero. LLIN brands are coded as 1 = DawaPlus 2.0, 2 = DuraNet, 3 = Interceptor, 4 = Netprotect, 5 = Olyset Net, 6 = PermaNet 2.0, 7 = PermaNet 3.0 and 8 = LifeNet. Sem. = semester (6 month period)

**Table 7:** Proportion of residual vectorial capacity of resistant *An. gambiae* s.l. prevented by improving LLIN durability properties, by country

Country	LLIN brand	Sem.	Intact scenario	Target content scenario	Maximum use scenario	Maximum survival scenario
Angola	1	2, 4	− 0.02 (− 0.51 to 0.27)	0.22 (− 0.17 to 0.45)	0.63 (0.42 to 0.76)	0.54 (0.28 to 0.69)
Benin	5	1, 2	0.24 (− 0.24 to 0.52)	0.36 (0.01 to 0.56)	0.61 (0.52 to 0.67)	0.20 (− 0.29 to 0.46)
Kenya	1, 2, 3, 4, 5, 6, 7	2, 4, 6, 8	0.07 (− 0.02 to 0.15)	0.08 (− 0.01 to 0.17)	0.80 (0.78 to 0.82)	0.21 (0.13 to 0.27)
Malawi	2, 3, 4, 5, 6	2, 4, 6	0.22 (0.10 to 0.31)	0.28 (0.18 to 0.37)	0.60 (0.57 to 0.63)	0.30 (0.19 to 0.38)
Mozambique	5, 6	2, 4, 6	0.02 (− 0.13 to 0.15)	0.17 (0.04 to 0.27)	0.83 (0.80 to 0.85)	0.03 (− 0.11 to 0.14)
Senegal	1, 4, 5, 6, 8	1, 2, 3, 4, 5, 6	0.10 (− 0.04 to 0.17)	0.08 (− 0.02 to 0.15)	0.86 (0.85 to 0.87)	0.03 (− 0.10 to 0.10)
Zambia	5, 6	2, 4	0.65 (0.46 to 0.77)	0.34 (0.14 to 0.47)	0.57 (0.50 to 0.63)	− 0.01 (− 0.30 to 0.19)

Numbers within parentheses are 95% credible intervals. Cells highlighted in green have credible intervals entirely above zero. LLIN brands are coded as 1 = DawaPlus 2.0, 2 = DuraNet, 3 = Interceptor, 4 = Netprotect, 5 = Olyset Net, 6 = PermaNet 2.0, 7 = PermaNet 3.0 and 8 = LifeNet. Sem. = semester (6 month period)

## Discussion

LLINs can lose effect over time because of attrition, non-use, physical damage, and loss of insecticidal effect, and these factors are not independent. They interact causally in ways that are not entirely obvious (Fig. 1). This paper is the first to pool data from multiple field studies of these factors to examine trends in durability across LLIN brands and countries. The primary conclusion is that while factors related to the classic three elements of LLIN durability (attrition, physical integrity, insecticidal activity) may contribute to non-use of LLINs, it is the (non-) use of LLINs that has the strongest effect on reducing VC.

The quantification of effects on VC provides a single metric for comparing the importance of these various factors. The analysis of counterfactuals suggests that increasing use of LLINs would be the most promising way to achieve greater protection from LLINs against malaria in most of the study settings. There is relatively little to be gained by further improving LLIN chemical durability because the LLINs are manufactured with active ingredient content well in excess of those at which the entomological effects saturate, and prolonged use in the field does not seem to lead to substantial loss of insecticidal effect. This conclusion holds even with levels of pyrethroid resistance observed in west African mosquito populations in 2006–2007 [21], which probably better reflect current levels of resistance across sub-Saharan Africa than do the fully susceptible mosquitoes. However, it would be useful to update the estimates of effect for resistant mosquitoes in experimental hut studies to reflect the increases in the frequency and intensity of resistance [22] to test whether this conclusion still holds under current levels of pyrethroid resistance as recent field studies indicate a substantial impact on malaria burden among users of synergist LLINs compared to standard pyrethroid only LLINs [23].

The physical durability of LLINs also appears to be unimportant in this analysis of the direct entomological effects of factors of decay, presumably because the (slowly decaying) pyrethroids still have sufficient deterrent and killing effects to counteract the effects of the physical barrier decaying. However, as physical damage to LLINs is an important driver of attrition and non-use [7, 24], then the analysis of counterfactuals understates its overall contribution to loss of effect. The lack of increase in hole area in nets in Kenya from 42 to 48 months after distribution suggests that the surviving LLINs at that stage may be a selected, well-cared-for subset. More generally, there is a need to extend this approach both to account for the causal relationships between the different factors (Fig. 1), access to alternative LLINs, whether already present in the household or available elsewhere, and selection bias resulting from disproportionate attrition of LLINs that are in poor condition (see Fig. 1).

LLIN use may be affected directly by the classical components of LLIN durability. For most of the country studies, the analysis of LLIN cohorts provides a way of separating out effects of attrition and use of LLINs, but these two factors interrelate. LLIN survivorship is an important precondition for use, but LLINs that are used less often may decay less and survive longer because they wear out more slowly than LLINs that are used more often. So, LLIN attrition, on its own, is a poor measure of programmatic success. Only LLINs that are being used can contribute to malaria transmission control, and LLINs that are present in households but are stored away and remain in good condition are useless until used. LLIN use, in addition to depending on presence, physical and chemical status of LLINs, depends on a plethora of factors including product quality, geographically specific use practices, knowledge about their benefits, and seasonally varying factors such as mosquito nuisance levels, temperature and humidity which impact the comfort level inside a LLIN [25–29]. Also, LLINs may be temporarily unavailable due to washing or travel.

These LLIN durability studies followed a cohort of newly distributed LLINs continually from distribution or shortly thereafter until the study LLINs were lost to follow up, without systematically tracking physical integrity, chemical content and use of non-cohort nets already present in the population at cohort distribution or coming into the population after cohort distribution. Therefore, studies with these designs are not informative about the influence of other (non-cohort) LLINs in the household on use. As such data on non-cohort nets were not available, in the calculations of this pooled analysis it was assumed that when present cohort LLINs were not used, no alternative (non-cohort) nets were used instead, thus potentially overestimating the gains that could be made by increasing use. Similarly, the calculations may have overestimated the gains that could be made by averting attrition of LLINs, since attrition can in reality correspond to replacement by new non-cohort nets.

One general finding from the prospective cohorts is that LLINs start early to accrue damage, and holed areas increase exponentially as they age and use declines (analyses that are not possible with data from cross-sectional surveys such as DHS, MIS and MICS, which do not generally collect data on LLIN physical integrity or chemical content). However, this does not tell us how much the accrual of holes is a consequence of use of the LLINs. Nor is it clear how much damage contributes to attrition and/or decline in use. Most studies do not cite damage as an important reason for non-use [25], though there are exceptions [30]. This could be because severely damaged LLINs are repurposed or discarded, i.e. contributing to attrition rather than non-use. Household acquisition of additional LLINs during the study periods was not systematically recorded in these studies, but some of the cohort LLINs were taken out of use because newer, non-cohort LLINs became available and this has been shown to be an important determinant of attrition rates [31–33]. Chemical decay is evidently not an important reason for either attrition or non-use, since for the LLIN brands included in this investigation, the insecticidal effects are generally retained through 3 years (Fig. 3). Accordingly, this potential effect on attrition is not included in Fig. 1.

Attrition shows complex patterns of variation between LLIN brands and countries and it is not possible to characterize products as consistently underperforming or over performing in terms of attrition. It is possible that the intense study follow-up may have resulted in some household members delaying the discarding of their LLINs, resulting in lower LLIN attrition. Attrition in Angola and Mozambique, where there were no repeated follow-up visits, is among the highest. However, important differences even for the same product were observed among countries that used similar methodology, indicating that the variation between countries in attrition is unlikely to be an artifact of differences in study design. Benin (with longitudinal follow up), had LLIN attrition as high as in Mozambique after 2 years.

The pooled analysis gives an overall impression that patterns of use of different LLIN brands vary locally. Local differences in knowledge, attitudes, climate, house architecture, socioeconomic status or availability of alternative LLINs could all contribute to such variation. In most countries, direct comparison of the use of different LLIN brands was impossible because of confounding with geographical region. For instance, in Senegal, Olyset Net and LifeNet were each distributed to separate areas, while three other products were mixed within the same villages. Olyset Net and LifeNet showed very different temporal use patterns from the other three products, which had similar use patterns amongst themselves.

The lack of information on the availability and use of alternative non-study nets might bias the results. While the current study suggests that LLIN use is possibly the predominant limiting factor of the impact of LLINs on VC, this needs to be studied in designs that allow for tracking the use of alternative nets either already present in the household upon distribution of a new LLIN cohort, or entering the household during the course of the study after the distribution of the cohort being followed.

Monitoring of all the different factors of LLIN durability remains important for national malaria control programmes. First, monitoring serves as a check to ensure that high quality LLINs are distributed in their countries. Second, LLIN monitoring is intended to guide national malaria control programmes on the optimal products for their countries as well as the optimal strategies to distribute those products. New LLIN products designed to address increasing pyrethroid resistance in vector populations are increasingly available. However, their performance under field conditions has not been thoroughly evaluated to determine their duration of effectiveness. Last, given that specific components of LLIN durability may affect LLIN use, continued monitoring—with input from social and behaviour scientists—may help to further define the most important parameters related to LLIN durability.

One advantage of this study is that it combined data from multiple countries using different LLIN brands to estimate overall trends in LLIN durability and use and how these contribute to the modeled impact of LLINs on malaria transmission as measured by VC. The large pooled sample size allowed for analysis of trends across different countries and LLIN brands that would not have been possible to do with individual country data. However, there are several caveats to this approach. First, the studies included were often of different design and utilized questionnaires that incorporated different questions or similar questions that were asked slightly differently. The data were combined with extensive consultation to ensure that they were as similar as possible. In many cases, this resulted in other explanatory variables (e.g. washing frequency and method, bed type) being dropped from the pooled analysis as they were only present in some studies. Second, modeling of the effect on VC relied on experimental hut data from 2012. Given the recent spread and intensification of pyrethroid resistance [22], updated data would help to refine the analysis, particularly for areas with high levels of pyrethroid resistance. Updated data should also include the impact of PBO or next generation LLINs which have increased efficacy against pyrethroid resistant mosquitoes. Lastly, the interrelations between components of LLIN durability and LLIN use need further elaboration to guide national malaria control programmes in developing strategies to maximize use.

## Conclusion

Both attrition and use of LLINs show substantial variation between countries, surveys, and LLIN brands, with more variation between countries than between LLIN brands. Given strong spatial variation in LLIN durability components, sometimes even among geographical sites within countries, future durability and cost-effectiveness studies aiming to compare LLIN products should randomize product-distributions within the same study communities. Low levels of use may reduce the potential impact of LLINs on transmission substantially, and variation in use may have a larger impact than variation in attrition per se. The entomological effects of chemical decay appear to be relatively small, while physical decay of LLINs may well be more important as a driver of attrition and non-use than as a direct cause of loss of effect of the LLINs. To maximize the cost-effectiveness of LLINs, emphasis should be on increasing use, though further research is needed to better understand how specific components of LLIN durability, as well as the timing and size of LLIN replenishment opportunities, may contribute to (non-) use of LLINs. Future studies should collect data on the presence, influx and use of non-cohort nets to provide important context for LLIN durability results.

## Supplementary information

**Additional file 1:** Bayesian model specification.

**Additional file 2:** Details of methods and results.

**Additional file 3:** Ethics approval and consent to participate.

## Data Availability

The datasets used and/or analysed during the current study are available from the corresponding author upon reasonable request.

## Abbreviations

BCC: Behaviour change communication
CI: Confidence interval
DHS: Demographic and Health Survey
DSS: Demographic Surveillance Site
ITN: Insecticide treated net
LLIN: Long lasting insecticidal net
Swiss TPH: Swiss Tropical and Public Health Institute
WHO: World Health Organization
CDC: Centers for Disease Control
PMI: President’s Malaria Initiative
INS: Instituto Nacional de Saúde
NMCP: National Malaria Control Programme
CREC: Centre de Recherche Entomologique de Cotonou
PNLP: Programme National de Lutte contre le Paludisme
KEMRI: Kenya Medical Research Institute
FST: Faculté des Sciences et Techniques
NMCC: National Malaria Control Centre
UCAD: Université Cheikh Anta Diop
