# Supplementary material for: Attrition, physical integrity and insecticidal activity of long-lasting insecticidal nets in sub-Saharan Africa and modelling of their impact on vectorial capacity
Source: Malar J. 2020 Aug 28;19:310. doi: 10.1186/s12936-020-03383-6 (PMC7456088; doi:10.1186/s12936-020-03383-6)
Supplement: Supplementary file 1 — Additional file 1: Bayesian model specification. [file 12936_2020_3383_MOESM1_ESM.docx]

**Additional file 1 to Briet *et al:* The durability of long-lasting insecticidal nets in sub-Saharan Africa: Bayesian Model Specification.**

This document comprises example code for key steps in the data analysis. The analysis was carried out in R (1) with regression models fitted using JAGS (2). Each of these scripts requires the libraries *R2WinBUGS* and *R2jags* to be loaded.

Scripts for the following analyses are included in this document:

[Estimation of LLIN survival from longitudinal (cohort) data 2](#_Toc40001873)

[Estimation of LLIN survival from cross-sectional survey data 3](#_Toc40001874)

[Analysis of holed surface area of nets with random effects due to the same nets being observed over several survey rounds 4](#_Toc40001875)

[Analysis of censoring of the holed area due to some nets having an unknown holed surface area above a threshold amount. 5](#_Toc40001876)

[Sampling of LLINs with correlated holed surface area and insecticide concentration 6](#_Toc40001877)

[Model for the relationship between mortality of mosquitoes exposed to LLIN netting material in cone tests and the chemical concentration in that LLIN netting material: version 1: country-specific coefficients. 7](#_Toc40001878)

[Model for the relationship between mortality of mosquitoes exposed to LLIN netting material in cone tests and the chemical concentration in that LLIN netting material: version 2: ratios in the coefficients for insecticide content for an LLIN product relative to a reference net, and assuming a common ratio across country studies 8](#_Toc40001879)

## Estimation of LLIN survival from longitudinal (cohort) data

model<-function(){

for(i in 1 : N){

S1[i] ~ dbern(p1)

S2[i] ~ dbern(res.p2[i])

res.p2[i] <- S1[i] * p2

S3[i] ~ dbern(res.p3[i])

res.p3[i] <- S2[i] * p3

S4[i] ~ dbern(res.p4[i])

res.p4[i] <- S3[i] * p4

S5[i] ~ dbern(res.p5[i])

res.p5[i] <- S4[i] * p5

S6[i] ~ dbern(res.p6[i])

res.p6[i] <- S5[i] * p6

}

p1 <- exp(logit.p1)/(1 + exp(logit.p1)) #inverse logit to get probability

p2 <- exp(logit.p2)/(1 + exp(logit.p2)) #inverse logit to get probability

p3 <- exp(logit.p3)/(1 + exp(logit.p3)) #inverse logit to get probability

p4 <- exp(logit.p4)/(1 + exp(logit.p4)) #inverse logit to get probability

p5 <- exp(logit.p5)/(1 + exp(logit.p5)) #inverse logit to get probability

p6 <- exp(logit.p6)/(1 + exp(logit.p6)) #inverse logit to get probability

pc1 <- p1

pc2 <- p1 * p2

pc3 <- p3 * pc2

pc4 <- p4 * pc3

pc5 <- p5 * pc4

pc6 <- p6 * pc5

logit.p1 ~ dnorm(0, 0.001)

logit.p2 ~ dnorm(0, 0.001)

logit.p3 ~ dnorm(0, 0.001)

logit.p4 ~ dnorm(0, 0.001)

logit.p5 ~ dnorm(0, 0.001)

logit.p6 ~ dnorm(0, 0.001)

}

Legend:

N = Number of nets, S1 = a vector of length N with values 0, 1 or NA for nets absent, present or unknown status in survey 1. S2, …, S6 as in S1 but for surveys 2 to 6.

## Estimation of LLIN survival from cross-sectional survey data

load.module("mix")

model<-function(){

for(i in 1 : N){

P[i] ~ dbetabin(a[i], b[i], T[i])

b[i] <- (p.eta[S[i]] * rho - p.eta[S[i]] - rho + 1) / rho

a[i] <- p.eta[S[i]] * (1 - rho) / rho

}

for(s in 1:2){

p.eta[s] <- exp(eta[s]) / (1 + exp(eta[s])) #inverse logit to get probability

eta[s] <- beta.0[s] #linear model on logit scale

beta.0[s] ~ dnorm(0, 0.001)

invlogit.beta.0[s] <- exp(beta.0[s]) / (1+exp(beta.0[s])) #inverse logit

}

rho ~ dunif(0,1)

}

Legend:

N = Number of households surveyed, T = Total number of marked nets present or destroyed in each household, P = Present nets in each household, S = Survey number for each observation

## Analysis of holed surface area of nets with random effects due to the same nets being observed over several survey rounds

model<-function(){

for(j in 1 : N) {

y[j] ~ dlnorm(mu[j], tau.rb[r[j], b[j]])

mu[j] <- beta.net[netcode[j]] +

beta.rb[2, 1]*round2[j] +

beta.rb[3, 1]*round3[j] +

beta.rb[4, 1]*round4[j] +

beta.rb[2, 2]*round2[j]*product2[j] +

beta.rb[3, 2]*round3[j]*product2[j] +

beta.rb[4, 2]*round4[j]*product2[j]

}

#Priors

for(m in 1 : M){

beta.net[m] ~ dnorm(beta.rb[1, netprod[m]], tau.re[netprod[m]])

}

for(r in 1 : 4){

for(b in 1 : 2){

beta.rb[r, b] ~ dnorm(0, 0.001)

tau.rb[r, b] ~ dgamma(0.01, 0.01)

}

}

for(b in 1 : 2){

tau.re[b] ~ dgamma(0.01, 0.01)

}

#Linear combinations

for(b in 1 : 2){

l.rb[1, b] <- beta.rb[1, b]

}

for(r in 2 : 4){

l.rb[r, 1] <- beta.rb[1, 1]+beta.rb[r, 1]

for(b in 2 : 2){

l.rb[r, b] <- beta.rb[1, b]+beta.rb[r, 1]+beta.rb[r, b]

}

}

for(r in 1 : 4){

for(b in 1 : 2){

tau.tot.rb[r, b] <- 1 / ((1 / tau.rb[r, b]) + (1 / tau.re[b]))

}

}

}

Legend:

The above example is for a study with four survey rounds r and two products b. N is the number of records; y is a vector of length N of the total holed surface area per net augmented with 1; r is a vector of length N with the survey round number, b is a vector of length N with the product number; round2, round3 and round4 are ‘dummy variable’ vectors of length N for survey rounds; product2 is a dummy variable vector of length N for the second product; netcode is a vector of length N of the net identifier number, M is the maximum net identifier number. The observed variable l.rb[r,b] is the estimated mean of the logarithmically transformed surface area of round r and product b, and tau.tot.rb[r,b] is the estimated precision of the logarithmically transformed surface area of round r and product b. The precision consists of the precision of the random effect per product (tau.re[b]), and the round and survey specific precision without random effects tau.rb[r,b].

## Analysis of censoring of the holed area due to some nets having an unknown holed surface area above a threshold amount.

model<-function(){

for(i in 1:N) {

is.censored[i] ~ dinterval(y[i], y.min[i])

y[i] ~ dlnorm(mu.rb[r[i],b[i]],tau.rb[r[i],b[i]])

}

#Priors

for(r in 1:3){

for(b in 1:3){

mu.rb[r,b] ~ dnorm(0, 0.001)

tau.rb[r,b] ~ dgamma(0.01, 0.01)

}

}

}

Legend:

The above model example is for a study with three survey rounds r and three products b. N is the number of records; y is the total holed surface area per net augmented with 1 and with missing values (NA) for censored observations; is.censored is a vector with values of 1 for censored observations and 0 otherwise; r is a vector with the survey round number, b is a vector with the product number; y.min is a vector with the minimum threshold holed area values for censored observations, For instance, if a net i had a recoded holed surface area of 10 cm^2^, plus an unknown amount of holed area due to seam failures, y[i]=NA, y.min[i]=10+1 and is.censored[i]=1.

## Sampling of LLINs with correlated holed surface area and insecticide concentration

model<-function(){

for(i in 1:N){

Chem[i] ~ dweib(shape,scale.trans[i])

scale.trans[i] <- (1/scale[i])^shape

scale[i] <- exp(log.scale[i])

log.scale[i] <- beta.0 + beta.1 * logHoles[i] + beta.2 * Use[i]

is.censored[i] ~ dinterval(logHoles[i], logHolesmaxvec[i,])

logHoles[i] ~ dnorm(mu.holes[Use[i] + 1], tau.holes[Use[i] + 1] * WEIGHTforHOLES[i])

Use[i] ~ dbern(p.use) #In case any nets have missing data for use

}

#Priors:

shape ~ dgamma(1.0, 0.0001) # slowly decreasing on positive reals

beta.0 ~ dnorm(0, 0.001)

beta.1 ~ dnorm(0, 0.001)

beta.2 ~ dnorm(0, 0.001)

mu.holes[1] ~ dnorm(0, 0.001)

mu.holes[2] ~ dnorm(0, 0.001)

tau.holes[1] ~ dgamma(1.0, 0.01)

tau.holes[2] ~ dgamma(1.0, 0.01)

logit.p.use ~ dnorm(0, 0.001)

p.use <- exp(logit.p.use)/(1 + exp(logit.p.use)) #inverse logit to get probability

#Joint prediction of holes and chemical content in a used net:

logHole.pred.used ~ dnorm(mu.holes[2], tau.holes[2])

log.scale.pred.used <- beta.0 + beta.1 * logHole.pred.used + beta.2 * 1

scale.pred.used <- exp(log.scale.pred.used)

scale.trans.pred.used <- (1 / scale.pred.used)^shape

Chem.pred.used ~ dweib(shape,scale.trans.pred.used)

mean.Chem.pred.used <-scale.pred.used*exp(loggam(1+1/shape))

}

Legend:

N = Number of nets, Chem = active ingredient in mg/m^2^, logHoles = the natural log of the total holed surface of the net calculated with the assumption that individual holes are elliptical shaped, augmented with 1, is.censored = a vector with values of 1 for censored observations and 0 otherwise, logHolesmaxvec = an N by 2 matrix with the first column containing the logHoles values, and the second column containing the natural log of the total holed surface of the net calculated with the assumption that individual holes are elliptical shaped, augmented with 11, Use = a vector with values 1 if the net was used the night prior to the survey, and 0 otherwise, and, WEIGHTforHOLES = a vector with values of 1, except for in a few records, where there were two measurements per net of holed surface area, and each received half weight (0.5).

## Model for the relationship between mortality of mosquitoes exposed to LLIN netting material in cone tests and the chemical concentration in that LLIN netting material: version 1: country-specific coefficients.

model<-function(){

for(i in 1:N){

D[i] ~ dbinom(p[i], T[i])

logit(p[i]) <- beta.0[country[i], roundnumber[i]] + beta.1[country[i],NetType[i]] * log(CHEM[i]+1)

}

for(i in 1:M){

Dc[i] ~ dbinom(pc[i], Tc[i])

logit(pc[i]) <- beta.0[countrycontrol[i], roundnumbercontrol[i]]

}

#Priors

for(k in 1:NumCountries){

for(l in 1:numrounds){

beta.0[k,l] ~ dnorm(mu.beta.0[k], tau.beta.0[k])

}

mu.beta.0[k] ~ dnorm(0, 0.001)

tau.beta.0[k] ~ dgamma(0.01, 0.01)

for(j in 1:NumNetTypes){

beta.1[k,j] ~ dnorm(0,0.001)

#Predictions

for(q in 1:Q){

logit(p.pred[q,j,k]) <- mu.beta.0[k] + beta.1[k,j] * log(CHEM.pred[q]+1)

}

}

for(j in 2:NumNetTypes){

ratio.beta.1[k,j] <-beta.1[k,j]/beta.1[k,1]

}

}

}

Legend:

N = the number of observations on LLIN samples,

M = the number of observations on untreated netting samples (controls),

D = the number of dead mosquitoes 24 hours after exposure to LLIN samples in cone tests,

Dc = the number dead mosquitoes 24 hours after exposure to control samples in cone tests,

T = the number of mosquitoes exposed to LLIN samples in cone tests,

Tc = the number mosquitoes exposed to control samples in cone tests,

CHEM = the insecticide concentration expressed in mg/m2,

country = the number of the country for the treatment observations,

countrycontrol = the number of the country for the control observations,

NumCountries = the total number of countries that have data for the two nets,

NetType = the number of the net product, where netType 1 is the reference net,

NumNetTypes = the total number of net products (typically 2),

roundnumber = the number of the survey round for the treatment observations,

roundnumbercontrol = the number of the survey round for the control observations,

numrounds = the total number of survey rounds,

CHEM.pred = a list of chemical concentrations for which predictions are required,

Q = the number of predictions required

## Model for the relationship between mortality of mosquitoes exposed to LLIN netting material in cone tests and the chemical concentration in that LLIN netting material: version 2: ratios in the coefficients for insecticide content for an LLIN product relative to a reference net, and assuming a common ratio across country studies

model<-function(){

for(i in 1:N){

D[i] ~ dbinom(p[i], T[i])

logit(p[i]) <- beta.0[country[i],roundnumber[i]] + beta.1.ReferenceNet[country[i]] * ratio.beta.1[NetType[i]] * log(CHEM[i]+1)

}

for(i in 1:M){

Dc[i] ~ dbinom(pc[i], Tc[i])

logit(pc[i]) <- beta.0[countrycontrol[i], roundnumbercontrol[i]]

}

#Priors

for(k in 1:NumCountries){

for(l in 1:numrounds){

beta.0[k,l] ~ dnorm(mu.beta.0[k], tau.beta.0[k])

}

mu.beta.0[k] ~ dnorm(0, 0.001)

tau.beta.0[k] ~ dgamma(0.01, 0.01)

beta.1.ReferenceNet[k] ~ dnorm(0,0.001)

for(j in 1:NumNetTypes){

beta.1[k,j] ~ dnorm(0,0.001)

#Predictions

for(q in 1:Q){

logit(p.pred[q,j,k]) <- mu.beta.0[k] + beta.1.ReferenceNet[k] * ratio.beta.1[j]*log(CHEM.pred[q]+1)

}

}

}

ratio.beta.1[1] <- 1

for(j in 2:NumNetTypes){

ratio.beta.1[j] ~ dnorm(0,0.001)

#ratio.beta.1[k,j] <-beta.1[k,j]/beta.1[k,1]

}

}

Legend:

see model version 1

**References**

1. R Core Development Team. R: A language and environment for statistical computing. Vienna, Austria: R Foundation for Statistical Computing; 2006.

2. Plummer M, editor JAGS: A Program for Analysis of Bayesian Graphical Models Using Gibbs Sampling2003; Vienna, Austria.
