# Supplementary material for: Attrition, physical integrity and insecticidal activity of long-lasting insecticidal nets in sub-Saharan Africa and modelling of their impact on vectorial capacity
Source: Malar J. 2020 Aug 28;19:310. doi: 10.1186/s12936-020-03383-6 (PMC7456088; doi:10.1186/s12936-020-03383-6)
Supplement: Supplementary file 2 — Additional file 2: Details of methods and results. [file 12936_2020_3383_MOESM2_ESM.docx]

##### Additional file 2

Contents

[Analysis of attrition, use, physical and chemical decay by country and net brand 2](#_Toc40013464)

[Attrition 2](#_Toc40013465)

[Use 2](#_Toc40013466)

[Physical integrity 3](#_Toc40013467)

[Active ingredient 3](#_Toc40013468)

[Tables of estimates of survival, use, physical integrity and active ingredient content 4](#_Toc40013469)

[Figures of estimates of durability 11](#_Toc40013470)

[Calibration of active ingredient lethality for different net brands. 16](#_Toc40013471)

[Introduction 16](#_Toc40013472)

[Methods 16](#_Toc40013473)

[Results 16](#_Toc40013474)

[Discussion 17](#_Toc40013475)

[Figures 18](#_Toc40013476)

[Parameterisation of LLIN effects as functions of their physical integrity and chemical content 24](#_Toc40013477)

[Introduction 24](#_Toc40013478)

[Methods 24](#_Toc40013479)

[Deterrence from entering huts 24](#_Toc40013480)

[Probabilities of attacking, pre-prandial and post-prandial killing for PermaNet 2.0 24](#_Toc40013481)

[Scaling insecticide coefficients for LLIN products other than PermaNet 2.0 25](#_Toc40013482)

[Results 25](#_Toc40013483)

[Tables and figures of parameters used in the modelling of vectorial capacity 26](#_Toc40013484)

[Simulation of effects on vectorial capacity 30](#_Toc40013485)

[Methods 30](#_Toc40013486)

[Simulation of insecticide content and holed area 30](#_Toc40013487)

[Vectorial capacity 30](#_Toc40013488)

[Proportion of vectorial capacity averted 30](#_Toc40013489)

[Sensitivity of the vectorial capacity averted to the different components of LLIN durability 30](#_Toc40013490)

[Results 31](#_Toc40013491)

[Discussion 32](#_Toc40013492)

[Tables of the potential effect of averting LLIN decay 34](#_Toc40013493)

[Figures illustrating the calculation of effects on vectorial capacity 45](#_Toc40013494)

[Figures illustrating the potential overall effects of averting LLIN decay 46](#_Toc40013495)

[Figures illustrating the potential effects of averting specific components of LLIN decay 48](#_Toc40013496)

[References 56](#_Toc40013497)

# Analysis of attrition, use, physical and chemical decay by country and net brand

This section contains the results of the durability analysis disaggregated for each net brand, separated by country. The patterns shown in the tables and figures are described in the narrative in each section.

Attrition

Figure S1 and Table S1 give the estimates of survivorship (1-attrition), disaggregated by country and net brand. These results illustrate the considerable variation between countries in attrition of the same product. For example in Angola, after two years (four semesters), only 58.8% (95% credible interval (CI): 48.6–68.5) of PermaNet 2.0 nets remained, whereas in Senegal, survivorship for this product was 97.2% (95% CI: 95.2–97.9) after two years. From two years onwards, there were differences in survivorship among products in Kenya, Malawi and Senegal. In Kenya and Malawi, survivorship was higher for Olyset than for PermaNet 2.0; in Mozambique, there was no difference between the two products, and in Senegal survivorship was lower for Olyset than for PermaNet 2.0. After three years, NetProtect had higher survivorship than both PermaNet 2.0 and Olyset in Kenya and Malawi, but there was no difference in Senegal. While NetProtect was among the best surviving nets in Kenya at the seventh semester (after 3.5 years) it showed a sharper decline than the other products between the seventh and eighth semesters.

## Use

Figure S2 and Table S2 illustrate the use of present (extant) LLINs, by net brand, for seven countries. There was a large variation in use among countries and among surveys. For example, use of present NetProtect nets varied from 1.3% (6^th^ semester) in Senegal to 98.6% (3^rd^ semester) in Malawi. Within countries, different products had similar use patterns over the surveys, with as notable examples LifeNet and Olyset in Senegal, which had patterns noticeably different from the other products. While use of present LLINs varied between follow up surveys, and declined for some products over time, such a decline was not apparent for four out of the seven country studies. The large credible interval in the 6^th^ survey for LifeNet in Senegal was due to a small number of records (13) with non-missing values for use the previous night.

Figure S3 and Table S3 illustrate the proportion of LLINs of the distributed cohorts that were used the previous night, by net brand, for the seven countries. This was obtained by multiplying the proportion of use of present LLINs (Figure S1 and Table S1) by the proportion of surviving nets of the cohort (Figure S3 and Table S3 in the appendix). This outcome variable allows, within countries with randomized LLIN allocation, comparing different products in how much they contribute to LLIN use over time. The results appear to be poorly consistent between country studies. For example. Interceptor had higher use than NetProtect, PermaNet 2.0 and Olyset in Kenya, but in Malawi, Interceptor had lower use than NetProtect, and had similar use to Olyset and PermaNet 2.0. Similarly, DuraNet performed better than NetProtect, PermaNet 2.0 and Olyset in Kenya, but had lower use than NetProtect, and similar use to Olyset and PermaNet 2.0 in Malawi. NetProtect had the highest use in Malawi, but was among the products with lowest use in Kenya and Senegal. PermaNet 2.0 had lower use than NetProtect in Malawi in several surveys, but had similar or slightly higher use in Kenya and Senegal. Also, PermaNet 2.0 had somewhat lower use than Dawaplus 2.0 in Kenya, but use of the two products was very similar Senegal.

The comparison of survivorship (Table S1) and use (Table S2), shows that these are not necessarily positively correlated. For instance, in Kenya, NetProtect and Olyset had relatively high survivorship in the 6^th^ and 7^th^ semesters, but with use taken into account, these products were among the low performers. In Senegal, survivorship was over 70% in the 5^th^ and 6^th^ semesters, but due to low use behaviour, the proportion nets in use was below 30% in three out of five products.

## Physical integrity

The holed areas of LLINs that had been reportedly used the night before sampling were assessed In exploratory analysis, it was found that the log-normal distribution (a Gaussian distribution after natural logarithmic transformation) fitted well to the data, as compared to other statistical distributions. Results are thus described in terms of the log-normal distribution. Figure S4 and Table S4 give the estimated means of the logarithmically transformed total holed surface area per LLIN used the previous night, by net product, for each country and each survey. The overall patterns are summarised in the main text. Within countries, different products had similar means over the surveys, but exceptions were LifeNet and Olyset in Senegal, which had patterns noticeably different from the other products, but this could be due to the distinct geographical areas of testing. Similarly Olyset had higher levels of damage than the other products in Malawi. In Kenya, where assignment was randomized, the mean for Olyset did not appear to be very different from the other products.

## Active ingredient

Figure S5 and Table S5 give the estimated means of insecticide content in nets that were used the previous night, by net product, for each country and each survey for which data were available. There was generally little decrease over time in the measured levels of the active insecticidal ingredients, with rather stable differences between LLIN brands in average content of active ingredients, indicating chemical stability. The differences in average content between LLIN brands are largely because of the different active agents (see Table 5 in the main text).

## Tables of estimates of survival, use, physical integrity and active ingredient content

**Table S1: Percent survival of LLINs**

| Country | Semester | Dawaplus 2.0 | DuraNet | Interceptor | NetProtect | Olyset | PermaNet 2.0 | PermaNet 3.0 | LifeNet |
| --- | --- | --- | --- | --- | --- | --- | --- | --- | --- |
| Angola | 2 | 90.2 (85.0-94.3) |  |  |  |  |  |  |  |
|  | 4 | 74.9 (68.2-81.3) |  |  |  |  |  |  |  |
| Benin | 1 |  |  |  |  | 98.9 (98.4-99.4) |  |  |  |
|  | 2 |  |  |  |  | 89.5 (87.9-91.1) |  |  |  |
|  | 3 |  |  |  |  | 81.0 (78.9-83.0) |  |  |  |
|  | 4 |  |  |  |  | 68.6 (65.9-71.2) |  |  |  |
| Kenya | 1 | 100 (99.9-100) | 100 (100-100) | 100 (99.9-100) | 100 (100.0-100) | 99.9 (99.1-100) | 99.6 (99.0-100) | 100 (99.9-100) |  |
|  | 2 | 99.2 (98.2-99.8) | 99.6 (99.0-100) | 99.5 (98.8-99.9) | 100 (99.9-100) | 99.7 (98.8-100) | 98.5 (97.3-99.3) | 99.8 (99.3-100) |  |
|  | 3 | 97.3 (95.7-98.6) | 99.6 (99.0-100) | 99.5 (98.8-99.9) | 99.8 (99.1-100) | 97.2 (95.5-98.6) | 97.6 (96.3-98.8) | 99.8 (99.3-100) |  |
|  | 4 | 94.8 (92.7-96.7) | 97.1 (95.6-98.3) | 99.1 (98.2-99.7) | 99.0 (97.9-99.7) | 97.2 (95.5-98.6) | 97.1 (95.5-98.4) | 99.1 (98.1-99.8) |  |
|  | 5 | 91.6 (88.9-94.1) | 97.1 (95.6-98.3) | 97.7 (96.2-98.8) | 99.0 (97.9-99.7) | 93.4 (90.8-95.7) | 89.0 (85.8-92.0) | 96.8 (95.0-98.2) |  |
|  | 6 | 87.3 (84.0-90.3) | 96.3 (94.7-97.8) | 91.4 (87.8-94.5) | 97.6 (95.9-98.9) | 91.2 (88.2-93.9) | 81.1 (77.1-84.8) | 95.1 (92.9-96.9) |  |
|  | 7 | 77.4 (72.7-81.8) | 94.4 (92.1-96.2) | 72.4 (67.2-77.6) | 91.0 (87.6-94.0) | 85.9 (82.1-89.5) | 71.0 (66.1-75.7) | 91.2 (88.3-93.8) |  |
|  | 8 | 66.7 (61.2-72.0) | 89.3 (86.1-92.0) | 50.4 (44.7-55.9) | 68.5 (62.9-74.2) | 81.6 (77.1-86.0) | 58.9 (52.7-64.8) | 88.5 (85.0-91.6) |  |
| Malawi | 1 |  | 100 (99.9-100) | 99.9 (99.1-100) | 99.8 (98.1-100) | 99.6 (98.9-100) | 100 (100.0-100) |  |  |
|  | 2 |  | 98.4 (97.0-99.5) | 99.4 (98.4-99.9) | 99.5 (97.7-100) | 99.5 (98.7-99.9) | 100 (99.7-100) |  |  |
|  | 3 |  | 96.4 (94.4-98.1) | 95.3 (92.9-97.4) | 99.5 (97.7-100) | 98.0 (96.3-99.1) | 97.4 (95.2-99.0) |  |  |
|  | 4 |  | 95.5 (93.5-97.3) | 88.9 (85.6-91.9) | 95.2 (93.1-96.9) | 95.0 (92.8-96.9) | 94.6 (91.9-96.8) |  |  |
|  | 5 |  | 87.8 (84.4-90.9) | 86.2 (82.7-89.4) | 93.8 (91.4-95.8) | 89.8 (86.7-92.5) | 82.5 (78.1-86.5) |  |  |
|  | 6 |  | 65.0 (59.5-70.1) | 57.9 (52.2-63.2) | 81.7 (77.4-85.6) | 70.4 (65.7-74.8) | 55.8 (50.3-61.5) |  |  |
| Mozambique | 2 |  |  |  |  | 98 (94.3-100) | 100 (99.6-100) |  |  |
|  | 4 |  |  |  |  | 94.7 (88.7-98.5) | 95.2 (90.8-98) |  |  |
|  | 6 |  |  |  |  | 84.6 (74.7-92.2) | 77.2 (69.6-84.1) |  |  |
| Senegal | 1 | 100 (99.8-100) |  |  | 100 (99.9-100) | 100 (99.9-100) | 99.8 (99.5-100) |  | 100 (99.8-100) |
|  | 2 | 98.7 (97.4-99.6) |  |  | 99.4 (98.4-99.9) | 99.4 (98.7-99.8) | 99.4 (98.6-99.9) |  | 99.4 (98.1-100) |
|  | 3 | 98.2 (96.6-99.3) |  |  | 98.4 (96.9-99.5) | 98.2 (97.0-99.2) | 98.5 (97.2-99.4) |  | 98.2 (96.3-99.4) |
|  | 4 | 97.5 (95.7-98.9) |  |  | 98.4 (96.9-99.4) | 92.3 (89.9-94.5) | 98.5 (97.2-99.4) |  | 98.2 (96.3-99.4) |
|  | 5 | 97.4 (95.5-98.8) |  |  | 94.7 (90.0-97.6) | 82.7 (79.2-86.0) | 97.8 (95.7-99.0) |  | 98.1 (96.0-99.4) |
|  | 6 | 97.4 (95.2-98.8) |  |  | 94.6 (89.9-97.6) | 76.0 (71.3-80.2) | 97.8 (95.5-99.0) |  | 98.0 (95.3-99.4) |
| Zambia | 2 |  |  |  |  | 100 (99.9-100) | 100 (100.0-100) |  |  |
|  | 3 |  |  |  |  | 89.8 (86.7-92.6) | 94.2 (91.6-96.5) |  |  |
|  | 4 |  |  |  |  | 77.3 (72.8-81.5) | 81.8 (77.7-85.5) |  |  |
|  | 5 |  |  |  |  | 57.3 (51.9-62.7) | 66.2 (60.5-71.5) |  |  |

Figures between brackets are 95% credible intervals from Bayesian models, except for Angola and Mozambique, for which exact confidence intervals were calculated.

**Table S2: Percent of those study nets present that were in use**

| Country | Semester | Dawaplus 2.0 | DuraNet | Interceptor | NetProtect | Olyset | PermaNet 2.0 | PermaNet 3.0 | LifeNet |
| --- | --- | --- | --- | --- | --- | --- | --- | --- | --- |
| Angola | 2 | 74.7 (65.4-83.0) |  |  |  |  |  |  |  |
|  | 4 | 56.4 (47.6-65.2) |  |  |  |  |  |  |  |
| Benin | 1 |  |  |  |  | 91.4 (87.1-95.0) |  |  |  |
|  | 2 |  |  |  |  | 86.4 (81.3-90.8) |  |  |  |
|  | 3 |  |  |  |  | 80.0 (74.1-85.5) |  |  |  |
|  | 4 |  |  |  |  | 79.3 (73.1-85.0) |  |  |  |
| Kenya | 1 | 73.2 (68.4-77.5) | 74.6 (70.0-78.5) | 81.0 (76.6-85.0) | 57.1 (52.2-61.9) | 68.6 (63.6-73.6) | 52.0 (46.4-57.1) | 64.0 (58.3-69.4) |  |
|  | 2 | 76.2 (71.2-80.8) | 82.4 (78.7-85.8) | 87.5 (84.3-90.4) | 55.9 (50.9-61.1) | 55.6 (49.8-61.1) | 60.0 (54.4-65.6) | 76.8 (71.9-81.6) |  |
|  | 3 | 85.4 (81.4-88.9) | 85.5 (82.2-88.6) | 90.8 (88.2-93.3) | 67.7 (62.7-72.3) | 59.7 (53.7-65.3) | 76.1 (71.4-80.5) | 78.9 (74.4-83.0) |  |
|  | 4 | 82.4 (78.2-86.4) | 88.5 (85.6-91.1) | 96.7 (95.2-98.0) | 74.7 (70.1-79.2) | 48.4 (42.1-54.6) | 78.1 (73.6-82.1) | 89.9 (86.7-92.6) |  |
|  | 5 | 79.4 (74.3-83.9) | 88.0 (84.9-90.8) | 93.5 (90.9-95.6) | 42.7 (36.9-48.7) | 45.5 (38.9-52.2) | 62.3 (55.7-68.6) | 87.0 (83.3-90.3) |  |
|  | 6 | 59.4 (52.4-66.1) | 78.6 (73.7-82.9) | 75.6 (69.4-81.2) | 54.4 (48.2-60.6) | 41.5 (34.8-48.3) | 81.0 (75.9-85.7) | 82.2 (77.1-86.7) |  |
|  | 7 | 61.5 (53.3-69.5) | 70.9 (64.7-76.7) | 91.7 (87.8-94.9) | 41.1 (34.7-47.8) | 38.3 (31.0-46.4) | 48.5 (40.9-56.1) | 63.8 (56.5-70.3) |  |
|  | 8 | 73.9 (65.8-80.8) | 69.7 (63.0-75.9) | 89.1 (83.4-93.6) | 45.0 (35.8-54.2) | 24.7 (17.8-32.5) | 69.7 (60.7-78.0) | 82.5 (76.8-87.6) |  |
| Malawi | 1 |  | 85.3 (82.2-88.3) | 71.4 (66.7-76.1) | 88.0 (84.2-91.2) | 73.9 (68.8-78.1) | 62.0 (56.1-67.7) |  |  |
|  | 2 |  | 77.0 (72.4-81.5) | 80.5 (75.7-84.8) | 83.4 (78.9-87.5) | 78.1 (73.2-82.7) | 73.5 (67.6-79.0) |  |  |
|  | 3 |  | 95.3 (92.2-97.5) | 97.9 (96.1-99.1) | 98.6 (97.1-99.5) | 94.6 (91.6-96.8) | 96.2 (93.9-97.9) |  |  |
|  | 4 |  | 87.4 (82.9-91.1) | 90.3 (86.2-93.6) | 95.6 (93.3-97.3) | 76.8 (71.7-81.7) | 75.6 (70.0-80.8) |  |  |
|  | 5 |  | 85.6 (81.3-89.7) | 89.8 (85.6-93.2) | 97.2 (95.2-98.5) | 85.5 (80.8-89.5) | 89.0 (84.4-92.8) |  |  |
|  | 6 |  | 82.3 (76.1-87.9) | 85.9 (80.0-90.9) | 91.3 (87.2-94.7) | 73.3 (67.2-78.9) | 76.4 (68.3-83.8) |  |  |
| Mozambique | 2 |  |  |  |  | 47.0 (33.7-60.8) | 49 (39.4-59) |  |  |
|  | 4 |  |  |  |  | 26.7 (16.2-38.8) | 40.0 (31.0-49.6) |  |  |
|  | 6 |  |  |  |  | 33.9 (21.2-47.8) | 43.0 (32.8-53.7) |  |  |
| Senegal | 1 | 62.9 (57.2-68.1) |  |  | 47.6 (41.1-54.8) | 84.3 (80.5-87.7) | 67.1 (61.9-71.9) |  | 21.7 (16.3-28.3) |
|  | 2 | 50.3 (43.5-56.5) |  |  | 34.5 (27.5-42.2) | 56.2 (52.1-60.3) | 50.4 (44.6-56.3) |  | 53.3 (45.4-60.8) |
|  | 3 | 60.5 (53.7-67.6) |  |  | 37.8 (30.0-45.8) | 87.1 (84.1-89.8) | 55.5 (49.2-61.9) |  | 45.9 (37.9-53.8) |
|  | 4 | 61.5 (53.2-68.8) |  |  | 25.3 (17.7-34.1) | 59.4 (54.6-64.2) | 52.8 (45.2-60.5) |  | 24.3 (14.5-36.2) |
|  | 5 | 19.6 (12.2-28.2) |  |  | 4.3 (1.5-8.9) | 92.0 (88.6-94.9) | 17.7 (11.2-25.7) |  | 9.2 (1.1-26.0) |
|  | 6 | 6.3 (2.5-11.6) |  |  | 1.3 (0.2-3.6) | 76.2 (69.3-82.5) | 6.5 (3.1-11.2) |  | 37.4 (12.7-65.3) |
| Zambia | 2 |  |  |  |  | 92.8 (88.7-95.2) | 94.5 (90.2-97.0) |  |  |
|  | 3 |  |  |  |  | 69.0 (63.2-74.8) | 85.9 (81.5-90.1) |  |  |
|  | 4 |  |  |  |  | 90.9 (86.8-94.4) | 88.7 (84.0-92.8) |  |  |
|  | 5 |  |  |  |  | 78.7 (70.4-85.8) | 87.2 (81.4-92.3) |  |  |

Figures between brackets are 95% credible intervals

Table S3: Percent of the original net cohort in use

| Country | Semester | Dawaplus 2.0 | DuraNet | Interceptor | NetProtect | Olyset | PermaNet 2.0 | PermaNet 3.0 | LifeNet |
| --- | --- | --- | --- | --- | --- | --- | --- | --- | --- |
| Angola | 2 | 59.9 (50.0-69.6) |  |  |  |  |  |  |  |
|  | 4 | 33.2 (26.0-40.9) |  |  |  |  |  |  |  |
| Benin | 1 |  |  |  |  | 90.5 (86.3-94.1) |  |  |  |
|  | 2 |  |  |  |  | 77.4 (72.7-81.6) |  |  |  |
|  | 3 |  |  |  |  | 64.9 (59.9-69.6) |  |  |  |
|  | 4 |  |  |  |  | 54.5 (49.8-59.0) |  |  |  |
| Kenya | 1 | 73.2 (68.4-77.5) | 74.6 (70.0-78.5) | 81.0 (76.6-85.0) | 57.1 (52.2-61.9) | 68.5 (63.4-73.5) | 51.8 (46.2-56.9) | 64.0 (58.3-69.4) |  |
|  | 2 | 75.5 (70.6-80.2) | 82.1 (78.5-85.6) | 87.1 (83.9-90.0) | 55.9 (50.9-61.0) | 55.4 (49.7-61.0) | 59.1 (53.5-64.6) | 76.7 (71.8-81.4) |  |
|  | 3 | 83.1 (79.0-86.7) | 85.2 (82.0-88.3) | 90.4 (87.7-92.8) | 67.5 (62.6-72.1) | 58.0 (52.1-63.5) | 74.3 (69.6-78.5) | 78.8 (74.2-82.9) |  |
|  | 4 | 78.1 (73.8-82.2) | 85.9 (82.8-88.8) | 95.9 (94.2-97.3) | 74.0 (69.3-78.4) | 47.0 (41.0-53.1) | 75.8 (71.4-80.1) | 89.1 (85.8-91.9) |  |
|  | 5 | 72.7 (67.6-77.4) | 85.5 (82.2-88.4) | 91.4 (88.5-93.7) | 42.3 (36.6-48.3) | 42.5 (36.4-48.9) | 55.5 (49.4-61.4) | 84.3 (80.4-87.7) |  |
|  | 6 | 51.8 (45.4-58.0) | 75.7 (70.9-80.0) | 69.1 (63.1-75.0) | 53.1 (47.0-59.2) | 37.8 (31.6-44.2) | 65.7 (60.4-70.7) | 78.2 (73.1-82.9) |  |
|  | 7 | 47.6 (40.9-54.4) | 66.9 (60.9-72.3) | 66.5 (60.9-71.9) | 37.4 (31.5-43.7) | 32.9 (26.5-39.9) | 34.4 (28.8-40.6) | 58.1 (51.1-64.5) |  |
|  | 8 | 49.3 (42.7-55.6) | 62.2 (56.1-68.1) | 44.9 (39.1-50.6) | 30.8 (24.3-37.9) | 20.1 (14.5-26.9) | 41.1 (34.5-47.8) | 73.0 (67.2-78.4) |  |
| Malawi | 1 |  | 85.3 (82.2-88.3) | 71.3 (66.6-76.1) | 87.8 (84.0-91.1) | 73.6 (68.6-77.8) | 62.0 (56.1-67.7) |  |  |
|  | 2 |  | 75.8 (71.1-80.4) | 80.0 (75.3-84.3) | 83.0 (78.4-87.2) | 77.7 (72.8-82.3) | 73.5 (67.5-79.0) |  |  |
|  | 3 |  | 91.8 (88.4-94.7) | 93.3 (90.4-95.8) | 98.0 (95.8-99.4) | 92.7 (89.6-95.3) | 93.6 (90.7-96.1) |  |  |
|  | 4 |  | 83.5 (78.9-87.3) | 80.3 (75.8-84.5) | 91.0 (88.1-93.4) | 73.0 (68.0-78.1) | 71.6 (66.2-76.8) |  |  |
|  | 5 |  | 75.2 (70.4-79.9) | 77.4 (72.7-81.7) | 91.1 (88.5-93.5) | 76.8 (72.0-81.3) | 73.4 (68.1-78.5) |  |  |
|  | 6 |  | 53.5 (47.4-59.3) | 49.7 (44.0-55.3) | 74.6 (69.5-79.2) | 51.6 (46.2-56.7) | 42.7 (36.7-48.9) |  |  |
| Mozambique | 2 |  |  |  |  | 46.1 (32.9-59.7) | 49.0 (39.4-59.0) |  |  |
|  | 4 |  |  |  |  | 23.9 (14.3-34.9) | 36.0 (27.6-44.9) |  |  |
|  | 6 |  |  |  |  | 24.6 (14.9-35.8) | 27.9 (20.6-35.9) |  |  |
| Senegal | 1 | 62.9 (57.2-68.1) |  |  | 47.6 (41.1-54.8) | 84.3 (80.5-87.7) | 67.0 (61.8-71.8) |  | 21.7 (16.3-28.3) |
|  | 2 | 49.6 (42.8-55.7) |  |  | 34.2 (27.3-41.9) | 55.7 (51.7-59.8) | 50.1 (44.3-56.0) |  | 52.6 (44.7-60.1) |
|  | 3 | 59.3 (52.5-66.3) |  |  | 37.0 (29.3-44.9) | 84.1 (80.9-87.1) | 54.3 (48.0-60.7) |  | 43.9 (36.2-51.7) |
|  | 4 | 58.1 (50.1-65.4) |  |  | 24.8 (17.4-33.3) | 53.9 (49.3-58.5) | 51.4 (43.9-58.9) |  | 23.3 (13.8-34.7) |
|  | 5 | 18.2 (11.2-26.2) |  |  | 3.7 (1.3-7.6) | 73.4 (68.9-77.6) | 16.4 (10.4-24.0) |  | 8.8 (1.0-24.9) |
|  | 6 | 5.7 (2.3-10.6) |  |  | 1.1 (0.2-3.1) | 55.3 (49.2-61.3) | 6.0 (2.7-10.3) |  | 35.3 (11.9-62.4) |
| Zambia | 2 |  |  |  |  | 92.8 (88.7-95.2) | 94.5 (90.2-97.0) |  |  |
|  | 3 |  |  |  |  | 61.9 (56.2-67.7) | 80.9 (76.2-85.4) |  |  |
|  | 4 |  |  |  |  | 70.3 (65.1-75.0) | 72.5 (67.3-77.6) |  |  |
|  | 5 |  |  |  |  | 45.1 (38.9-51.2) | 57.8 (51.6-63.6) |  |  |

Figures between brackets are 95% credible intervals

Table S4: Mean of log-transformed holed area (in cm^2^) in used LLINs by semester (half year)

| Country | Semester | DawaPlus 2.0 | DuraNet | Interceptor | Netprotect | Olyset | PermaNet 2.0 | PermaNet 3.0 | LifeNet |
| --- | --- | --- | --- | --- | --- | --- | --- | --- | --- |
| Angola | 2 | 3.3 (2.8-3.8) |  |  |  |  |  |  |  |
|  | 4 | 3.7 (3.2-4.1) |  |  |  |  |  |  |  |
| Benin | 1 |  |  |  |  | 2.0 (1.7-2.4) |  |  |  |
|  | 2 |  |  |  |  | 3.5 (3.1-3.8) |  |  |  |
|  | 3 |  |  |  |  | 4.0 (3.6-4.4) |  |  |  |
|  | 4 |  |  |  |  | 4.1 (3.7-4.4) |  |  |  |
| Kenya | 1 | 2.4 (0.5-4.5) | 0.1 (-0.1-0.3) | 1.4 (0.4-2.3) | 1.3 (-0.3-2.9) | 1.7 (0.6-2.8) | 2.5 (0.7-4.5) | 0.7 (0.2-1.2) |  |
|  | 2 | 1.5 (0.7-2.3) | 1.1 (0.3-1.9) | 2.3 (0.9-3.8) | 2.4 (0.6-4.3) | 1.3 (0.2-2.5) | 1.2 (0.1-2.4) | 1.5 (0.5-2.5) |  |
|  | 3 | 1.8 (1.0-2.7) | 2.0 (1.2-2.9) | 2.0 (1.1-2.8) | 2.2 (1.1-3.3) | 3.0 (2.0-4.1) | 3.3 (2.0-4.7) | 2.2 (1.1-3.4) |  |
|  | 4 | 2.1 (1.2-3.2) | 1.2 (0.7-1.7) | 2.4 (1.6-3.3) | 2.7 (1.9-3.5) | 3.9 (2.7-5.1) | 3.1 (1.7-4.5) | 1.4 (0.5-2.2) |  |
|  | 5 | 3.9 (2.2-5.9) | 1.4 (0.6-2.1) | 3.1 (1.9-4.2) | 2.8 (1.7-4.0) | 4.5 (2.4-6.8) | 3.7 (2.4-5.1) | 2.4 (1.5-3.3) |  |
|  | 6 | 4.7 (3.2-6.7) | 2.5 (1.7-3.4) | 3.6 (2.6-4.5) | 4.7 (3.7-5.8) | 4.7 (3.9-5.5) | 5.1 (3.9-6.5) | 3.6 (2.8-4.5) |  |
|  | 7 | 5.3 (3.8-7.3) | 3.4 (2.6-4.2) | 5.7 (4.4-7.2) | 5.4 (4.3-6.6) | 5.5 (4.2-6.9) | 5.6 (4.0-7.6) | 4.6 (3.3-6.0) |  |
|  | 8 | 6.4 (4.6-9.1) | 3.3 (2.3-4.3) | 4.8 (3.8-5.9) | 4.6 (3.3-5.9) | 4.8 (3.7-5.9) | 3.9 (2.6-5.3) | 3.3 (2.6-4.1) |  |
| Malawi | 1 |  | 1.1 (0.1-2.0) | 0.4 (-0.1-1.0) | 0.7 (0.1-1.3) | 2.2 (1.1-3.2) | 0.7 (-0.1-1.6) |  |  |
|  | 2 |  | 1.7 (1.0-2.4) | 2.1 (1.3-3.0) | 1.6 (0.8-2.5) | 2.9 (1.4-4.3) | 1.7 (0.2-3.3) |  |  |
|  | 3 |  | 1.9 (1.0-2.9) | 3.2 (2.1-4.4) | 3.3 (2.1-4.6) | 4.5 (3.2-5.7) | 1.7 (0.7-2.7) |  |  |
|  | 4 |  | 2.3 (1.5-3.0) | 3.4 (2.4-4.3) | 3.1 (2.4-3.8) | 5.8 (4.4-7.3) | 2.7 (1.5-3.8) |  |  |
|  | 5 |  | 2.6 (1.7-3.5) | 4.6 (3.7-5.4) | 3.5 (2.7-4.2) | 5.6 (4.7-6.5) | 3.9 (3.1-4.7) |  |  |
|  | 6 |  | 3.3 (2.8-3.8) | 4.6 (4.1-5.1) | 4.1 (3.6-4.5) | 5.8 (5.3-6.3) | 5.3 (4.6-5.9) |  |  |
| Mozambique | 2 |  |  |  |  | 4.8 (4.0-5.7) | 1.6 (1.1-2.2) |  |  |
|  | 4 |  |  |  |  | 5.3 (3.9-6.7) | 3.3 (2.7-3.9) |  |  |
|  | 6 |  |  |  |  | 7.1 (5.8-8.4) | 5.0 (4.3-5.6) |  |  |
| Senegal | 1 | 0.2 (0.1-0.4) |  |  | 0.1 (0.0-0.2) | 0.7 (0.6-0.8) | 0.2 (0.1-0.3) |  | 0.4 (0.1-0.7) |
|  | 2 | 0.5 (0.3-0.8) |  |  | 0.3 (0.1-0.5) | 1.4 (1.2-1.7) | 0.5 (0.3-0.7) |  | 0.1 (0.0-0.2) |
|  | 3 | 1.3 (0.9-1.6) |  |  | 0.6 (0.3-0.9) | 1.6 (1.4-1.8) | 1.1 (0.8-1.4) |  | 0.1 (0.0-0.2) |
|  | 4 | 1.8 (1.3-2.2) |  |  | 1.1 (0.5-1.7) | 3.5 (3.2-3.8) | 1.5 (1.0-1.9) |  | 0.3 (0.0-0.6) |
|  | 5 | 1.5 (0.5-2.6) |  |  | 0.4 (-0.5-1.3) | 3.0 (2.6-3.3) | 1.6 (0.6-2.7) |  | 0.6 (-2.4-3.3) |
|  | 6 | 0.9 (-0.5-2.5) |  |  | 1.9 (-6.4-9.6) | 4.1 (3.6-4.5) | 3.3 (1.1-5.5) |  | 1.7 (-1.2-4.6) |
| Zambia | 2 |  |  |  |  | 2.4 (2.2-2.6) | 2.1 (1.9-2.3) |  |  |
|  | 3 |  |  |  |  | 3.2 (3.0-3.4) | 2.9 (2.7-3.1) |  |  |
|  | 4 |  |  |  |  | 3.9 (3.7-4.2) | 3.9 (3.7-4.2) |  |  |
|  | 5 |  |  |  |  | 4.5 (4.3-4.8) | 4.3 (4.0-4.7) |  |  |

Figures between brackets are 95% credible intervals

Table S5: Mean of log-transformed insecticide content (in mg/m^2^) in used LLINs by semester (half year)

| Country | Semester | DawaPlus 2.0 | DuraNet | Interceptor | Netprotect | Olyset | PermaNet 2.0 | PermaNet 3.0 | LifeNet |
| --- | --- | --- | --- | --- | --- | --- | --- | --- | --- |
| Angola | 2 | 3.9 (3.3-4.5) |  |  |  |  |  |  |  |
|  | 4 | 3.4 (2.8-4.1) |  |  |  |  |  |  |  |
| Benin* | 1 |  |  |  |  | 5.7 (5.4-5.9) |  |  |  |
|  | 2 |  |  |  |  | 4.5 (3.8-5.4) |  |  |  |
| Kenya | 2 | 3.9 (3.4-4.5) | 5.0 (4.8-5.2) | 4.9 (4.5-5.4) | 3.8 (3.6-4.0) | 6.8 (6.7-6.9) | 3.3 (2.9-3.7) | 3.3 (2.5-4.1) |  |
|  | 4 | 3.5 (2.5-4.5) | 4.9 (4.6-5.1) | 4.6 (3.8-5.4) | 3.8 (3.7-3.9) | 6.7 (6.6-6.8) | 3.0 (2.2-3.7) | 3.1 (1.3-4.9) |  |
|  | 6 | 3.4 (2.4-4.4) | 4.8 (4.7-4.9) | 4.5 (3.7-5.3) | 4.4 (3.8-5.0) | 6.9 (6.7-7.0) | 2.9 (2.3-3.5) | 3.3 (2.7-3.9) |  |
|  | 8 | 3.2 (2.0-4.4) | 4.5 (4.3-4.6) | 4.4 (4.0-4.9) | 4.2 (3.9-4.6) | 6.6 (6.2-6.9) | 2.8 (1.4-4.2) | 3.2 (2.2-4.1) |  |
| Malawi | 2 |  | 5.4 (5.1-5.8) | 4.6 (4.0-5.2) | 3.8 (3.7-4.0) | 6.9 (6.8-7.0) | 3.5 (2.9-4.1) |  |  |
|  | 4 |  | 5.1 (4.8-5.5) | 3.7 (2.6-4.8) | 3.4 (3.2-3.6) | 6.7 (6.5-6.9) | 2.8 (1.5-4.1) |  |  |
|  | 6 |  | 5.2 (4.6-5.9) | 3.6 (2.6-4.6) | 2.6 (1.9-3.3) | 6.6 (6.4-7.0) | 2.1 (0.5-3.8) |  |  |
| Mozambique | 2 |  |  |  |  | 6.5 (6.3-6.7) | 3.3 (3.0-3.7) |  |  |
|  | 4 |  |  |  |  | 6.3 (6.0-6.7) | 2.8 (2.4-3.3) |  |  |
|  | 6 |  |  |  |  | 6.4 (6.2-6.7) | 2.1 (1.1-2.9) |  |  |
| Senegal | 1 |  |  |  | 3.9 (3.7-4.0) | 6.6 (6.4-6.8) |  |  | 5.5 (5.1-5.9) |
|  | 2 |  |  |  | 3.7 (3.5-3.9) | 6.5 (6.4-6.7) |  |  | 5.1 (4.8-5.5) |
|  | 3 | 3.8 (3.4-4.1) |  |  | 4.0 (3.5-4.5) | 6.4 (6.2-6.6) | 4.0 (3.6-4.3) |  | 5.2 (5.0-5.4) |
|  | 4 | 3.9 (3.2-4.6) |  |  | 3.2 (3.0-3.5) | 6.4 (6.3-6.6) | 3.6 (3.3-3.9) |  | 5.4 (5.2-5.6) |
|  | 5 | 3.9 (3.0-4.9) |  |  | 4.0 (3.6-4.7) | 6.3 (6.0-6.6) | 3.6 (3.2-4.2) |  | 5.3 (4.6-5.9) |
|  | 6 | 4.3 (3.5-5.5) |  |  | 3.9 (3.5-4.3) | 6.2 (6.0-6.4) | 3.6 (3.2-4.2) |  | 5.2 (4.5-6.0) |
| Zambia | 2 |  |  |  |  | 7.0 (6.9-7.0) | 3.7 (3.3-4.2) |  |  |
|  | 5 |  |  |  |  | 6.8 (6.7-6.9) | 3.0 (2.2-3.7) |  |  |

Figures between brackets are 95% credible intervals *Olyset nets from Benin were tested using a different assay using gas chromatography (GC) [1]. For this analysis, the measurements were rescaled (multiplied by 52.66) assuming the insecticide content in new Olyset nets was the same in Benin and Mozambique.

Table S6: Percentage of vectorial capacity of susceptible An. gambiae s.l. averted by LLINs

| Country | Semester | DawaPlus 2.0 | DuraNet | Interceptor | Netprotect | Olyset | PermaNet 2.0 | PermaNet 3.0 | LifeNet |
| --- | --- | --- | --- | --- | --- | --- | --- | --- | --- |
| Angola | 2 | 97.3 (94.6-98.7) |  |  |  |  |  |  |  |
|  | 4 | 84.5 (77.4-90.1) |  |  |  |  |  |  |  |
| Benin | 1 |  |  |  |  | 99.8 (99.7-99.9) |  |  |  |
|  | 2 |  |  |  |  | 94.9 (93.5-96.0) |  |  |  |
| Kenya | 2 | 99.5 (99.1-99.7) | 99.6 (99.3-99.7) | 99.9 (99.9-100) | 97.3 (96.2-98.2) | 97.0 (95.4-98.0) | 97.6 (96.5-98.5) | 97.0 (95.4-98.1) |  |
|  | 4 | 99.5 (99.3-99.7) | 99.8 (99.6-99.9) | 100 (100-100) | 99.5 (99.2-99.7) | 92.8 (89.7-95.1) | 98.6 (98.0-99.0) | 99.5 (99.1-99.7) |  |
|  | 6 | 93.7 (90.8-95.7) | 99.2 (98.6-99.5) | 98.4 (97.6-99.0) | 95.9 (93.6-97.3) | 87.4 (82.1-91.3) | 95.8 (94.4-97.0) | 97.5 (96.2-98.4) |  |
|  | 8 | 93.2 (89.8-95.5) | 97.6 (95.9-98.5) | 91.6 (88.1-94.1) | 82.6 (74.8-88.6) | 63.9 (50.7-73.3) | 85.0 (79.3-89.6) | 96.4 (94.5-97.6) |  |
| Malawi | 2 |  | 98.7 (97.9-99.2) | 99.7 (99.5-99.8) | 99.9 (99.7-99.9) | 99.3 (99.0-99.6) | 99.4 (98.9-99.6) |  |  |
|  | 4 |  | 99.6 (99.3-99.8) | 98.9 (98.4-99.2) | 99.9 (99.9-100) | 97.9 (97.0-98.4) | 98.3 (97.5-98.9) |  |  |
|  | 6 |  | 93.4 (90.4-95.5) | 91.3 (87.9-93.7) | 97.4 (96.5-98.1) | 91.8 (89.4-93.9) | 77.9 (72.0-82.6) |  |  |
| Mozambique | 2 |  |  |  |  | 82.7 (69.7-90.0) | 91.0 (84.7-95.0) |  |  |
|  | 4 |  |  |  |  | 57.7 (39.3-73.0) | 83.9 (74.9-90.4) |  |  |
|  | 6 |  |  |  |  | 56.3 (39.7-71.5) | 63.5 (51.5-73.0) |  |  |
| Senegal | 1 |  |  |  | 95.0 (92.0-97.1) | 99.3 (99.1-99.5) |  |  | 59.9 (50.4-68.1) |
|  | 2 |  |  |  | 87.3 (81.2-92.4) | 95.6 (94.4-96.6) |  |  | 94.1 (90.6-96.6) |
|  | 3 | 97.5 (96.2-98.6) |  |  | 89.1 (83.0-93.8) | 99.0 (98.8-99.3) | 96.5 (94.4-98.0) |  | 89.8 (83.9-93.7) |
|  | 4 | 97.2 (95.4-98.4) |  |  | 76.9 (65.3-86.6) | 89.7 (87.6-91.6) | 95.6 (93.0-97.5) |  | 64.9 (46.7-80.5) |
|  | 5 | 62.0 (45.1-76.0) |  |  | 21.2 (9.2-38.2) | 96.9 (96.2-97.6) | 62.3 (46.6-76.7) |  | 30.9 (5.0-67.9) |
|  | 6 | 27.4 (12.3-47.2) |  |  | 7.2 (1.2-18.6) | 88.0 (84.9-90.6) | 29.2 (15.3-44.8) |  | 78.4 (46.3-97.1) |
| Zambia | 2 |  |  |  |  | 99.9 (99.9-100) | 100 (100-100) |  |  |
|  | 4 |  |  |  |  | 99.8 (99.7-99.9) | 99.7 (99.4-99.8) |  |  |

Figures between round brackets are 95% credible intervals.

Table S7: Percentage of vectorial capacity of resistant *An. gambiae* s.l. averted by LLINs

| Country | Sem-ester | DawaPlus 2.0 | DuraNet | Interceptor | Netprotect | Olyset | PermaNet 2.0 | PermaNet 3.0 | LifeNet |
| --- | --- | --- | --- | --- | --- | --- | --- | --- | --- |
| Angola | 2 | 84.0 (76.1-90.1) |  |  |  |  |  |  |  |
|  | 4 | 54.9 (45.8-64.4) |  |  |  |  |  |  |  |
| Benin | 1 |  |  |  |  | 96.3 (93.9-97.8) |  |  |  |
|  | 2 |  |  |  |  | 76.0 (71.9-79.8) |  |  |  |
| Kenya | 2 | 94.5 (92.3-96.3) | 97.5 (96.3-98.5) | 97.8 (97.0-98.5) | 79.3 (74.6-83.2) | 71.8 (66.0-77.2) | 81.8 (77.2-85.9) | 92.2 (88.9-94.8) |  |
|  | 4 | 94.0 (92.0-95.8) | 98.6 (98.0-99.2) | 99.0 (98.7-99.2) | 92.7 (90.4-94.8) | 56.8 (50.2-62.8) | 87.4 (84.6-90.0) | 97.7 (96.5-98.6) |  |
|  | 6 | 71.6 (65.4-77.5) | 95.6 (93.7-97.1) | 84.3 (79.7-88.1) | 77.3 (71.8-82.0) | 46.5 (39.8-53.7) | 77.7 (73.5-81.8) | 92.0 (88.5-94.7) |  |
|  | 8 | 69.1 (62.0-75.5) | 88.8 (84.7-92.4) | 61.8 (55.6-67.4) | 51.9 (42.6-60.6) | 24.3 (17.3-31.4) | 54.9 (47.0-61.9) | 88.2 (84.1-91.7) |  |
| Malawi | 2 |  | 94.6 (92.4-96.5) | 94.1 (91.9-95.9) | 97.0 (95.4-98.1) | 85.4 (82.0-88.4) | 92.5 (89.1-95.1) |  |  |
|  | 4 |  | 97.6 (96.2-98.5) | 86.4 (83.4-88.8) | 96.1 (95.1-97.0) | 74.0 (70.2-77.4) | 85.6 (81.1-89.2) |  |  |
|  | 6 |  | 80.0 (74.4-84.9) | 61.1 (55.1-66.6) | 76.5 (72.6-80.0) | 54.8 (49.8-59.8) | 43.2 (37.2-48.8) |  |  |
| Mozambique | 2 |  |  |  |  | 40.3 (28.7-51.7) | 64.0 (49.9-77.0) |  |  |
|  | 4 |  |  |  |  | 20.8 (12.3-30.2) | 48.4 (33.9-62.1) |  |  |
|  | 6 |  |  |  |  | 21.5 (13.3-31.0) | 29.8 (18.9-41.1) |  |  |
| Senegal | 1 |  |  |  | 72.0 (65.1-78.6) | 84.6 (82.7-86.4) |  |  | 39.6 (31.8-47.1) |
|  | 2 |  |  |  | 55.0 (45.7-64.1) | 64.7 (60.7-68.3) |  |  | 81.0 (74.4-86.9) |
|  | 3 | 84.2 (79.2-88.8) |  |  | 60.4 (50.4-69.9) | 82.4 (80.2-84.4) | 83.2 (77.8-87.8) |  | 73.9 (65.5-81.6) |
|  | 4 | 82.8 (76.6-88.0) |  |  | 37.9 (27.8-49.2) | 50.8 (47.3-54.5) | 77.1 (69.8-83.2) |  | 44.8 (28.1-62.6) |
|  | 5 | 34.2 (23.1-47.2) |  |  | 8.2 (3.0-16.2) | 70.8 (67.6-73.7) | 34.7 (22.6-47.9) |  | 18.2 (2.6-47.2) |
|  | 6 | 13.4 (5.6-23.5) |  |  | 2.5 (0.4-6.6) | 50.3 (45.5-54.9) | 12.9 (6.4-21.8) |  | 60.3 (26.5-88.6) |
| Zambia | 2 |  |  |  |  | 95.2 (93.9-96.2) | 99.4 (99.1-99.7) |  |  |
|  | 4 |  |  |  |  | 92.1 (89.9-93.8) | 93.5 (91.3-95.2) |  |  |

Figures between round brackets are 95% credible intervals.

## Figures of estimates of durability

**
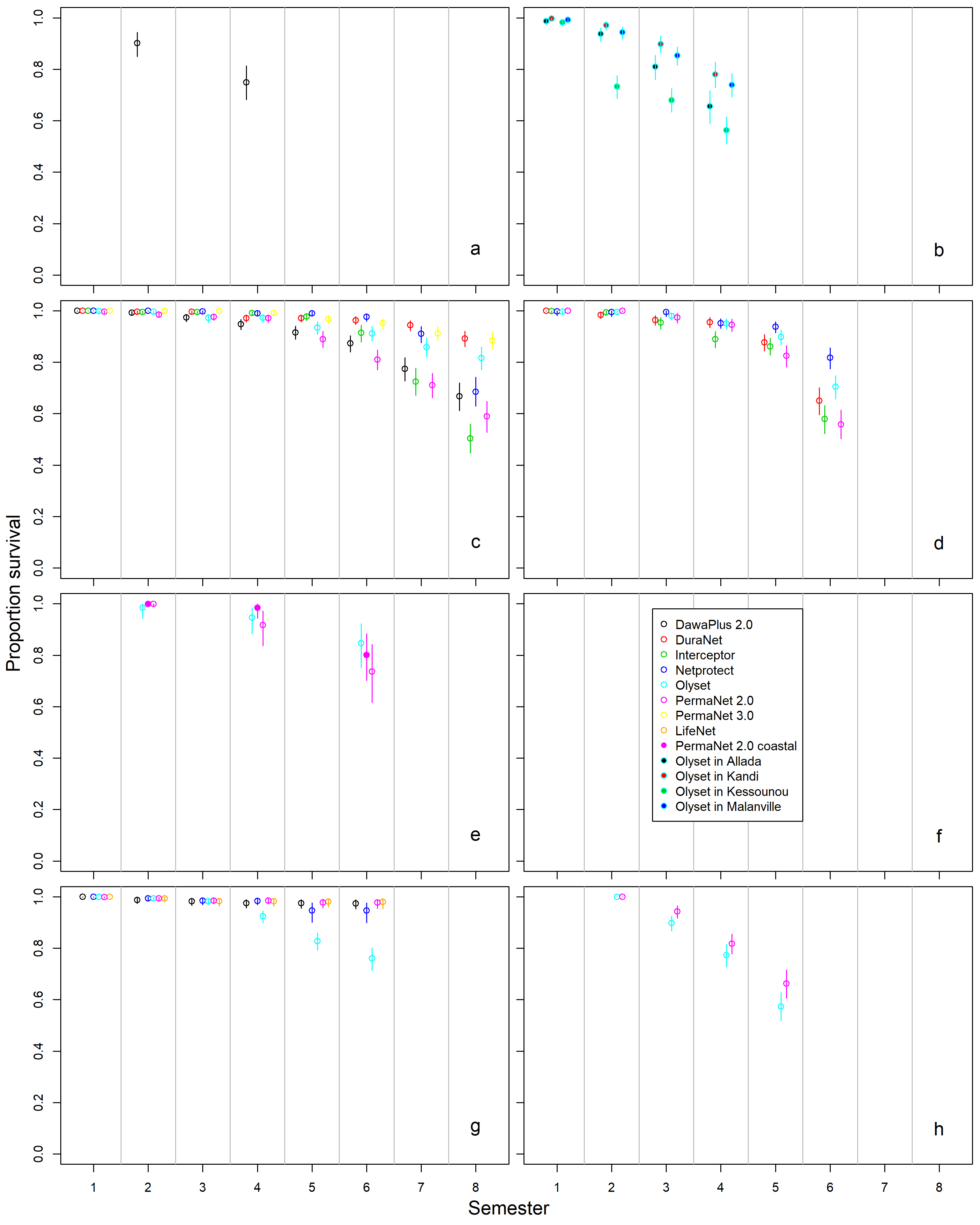
**

Figure S1. Proportion survival of LLINs by time since distribution

a Angola, b Benin, c Kenya, d Malawi, e Mozambique, f legend, g Senegal and h Zambia. Coloured vertical bars show 95% credible intervals.

**
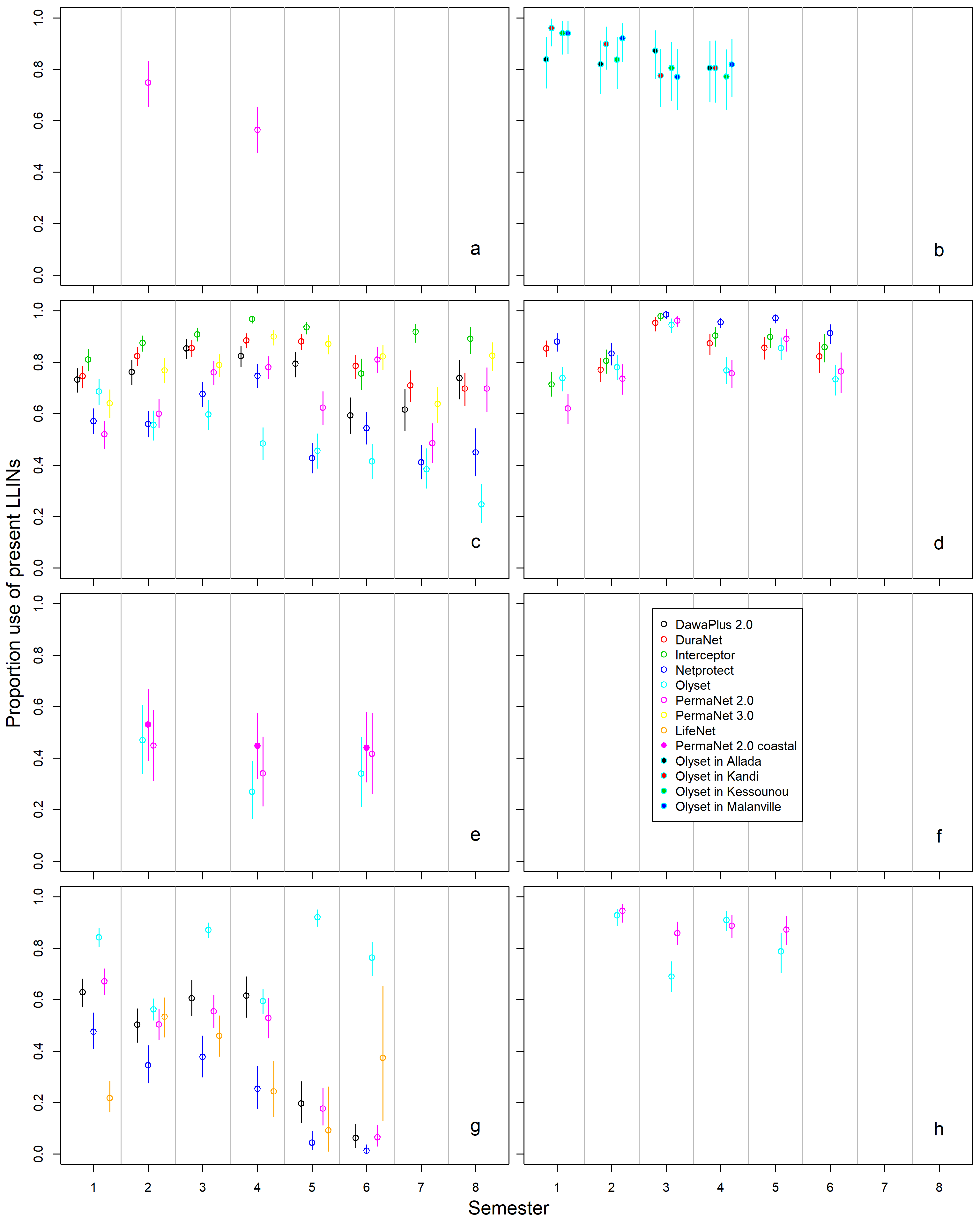
**

Figure S2. Proportion use of study LLINs present

a Angola, b Benin, c Kenya, d Malawi, e Mozambique, f legend, g Senegal and h Zambia. Coloured vertical bars show 95% credible intervals.


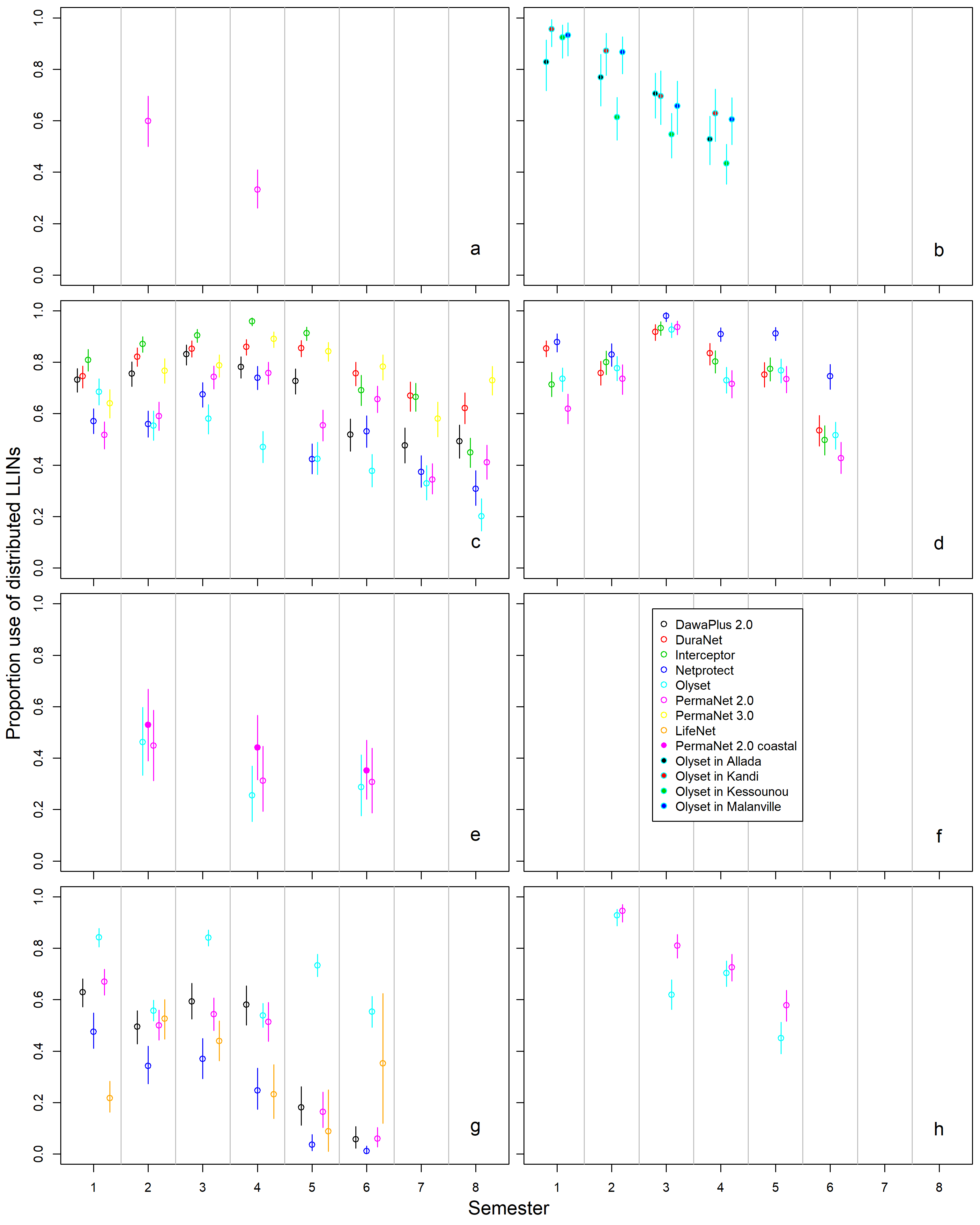


Figure S3. Proportion use of the original net cohort

a Angola, b Benin, c Kenya, d Malawi, e Mozambique, f legend, g Senegal and h Zambia. Coloured vertical bars show 95% credible intervals.


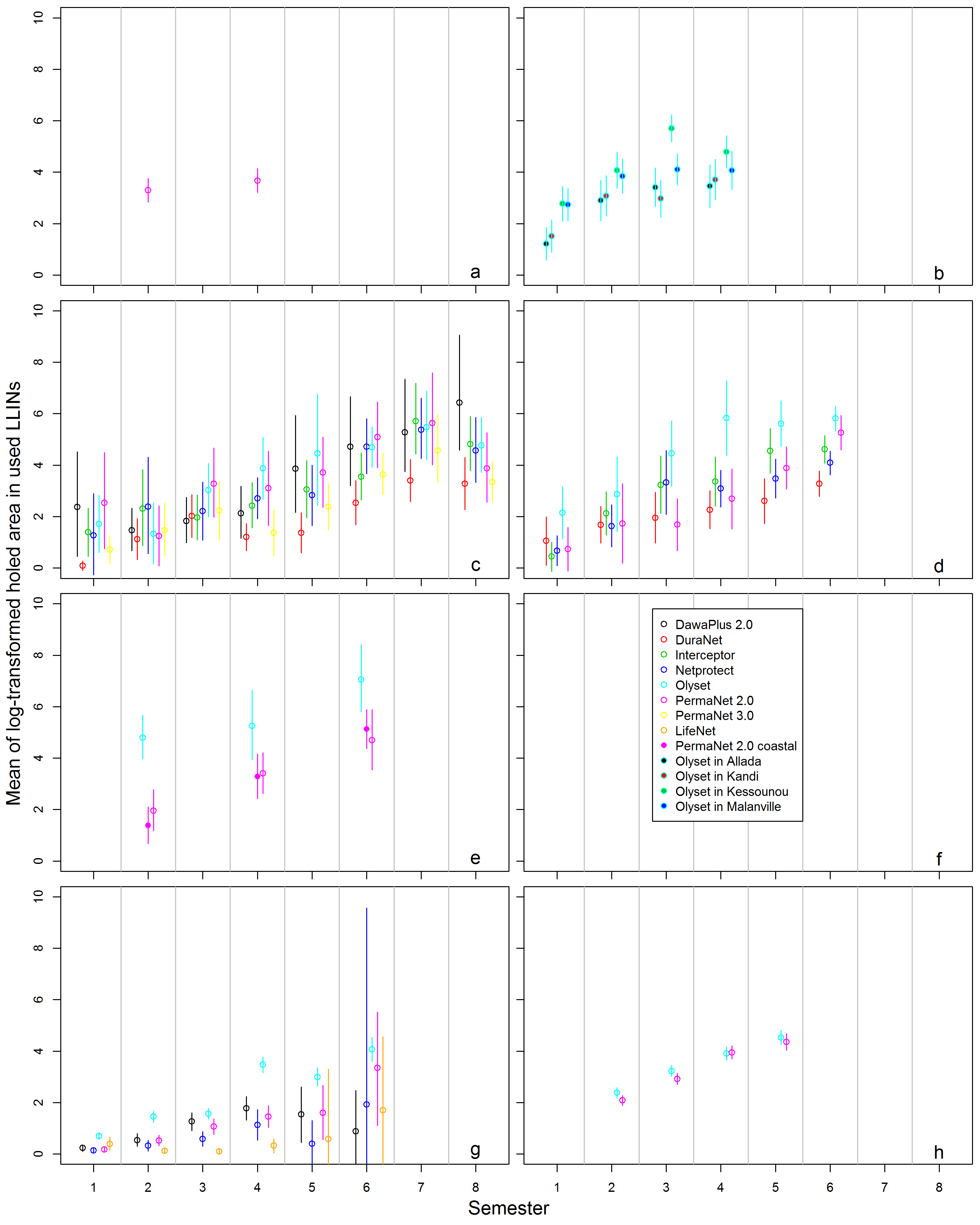


Figure S4. Mean of log-transformed holed area (in cm^2^) in used LLINs by semester (half year)

a Angola, b Benin, c Kenya, d Malawi, e Mozambique, f legend, g Senegal and h Zambia. Coloured vertical bars show 95% credible intervals.


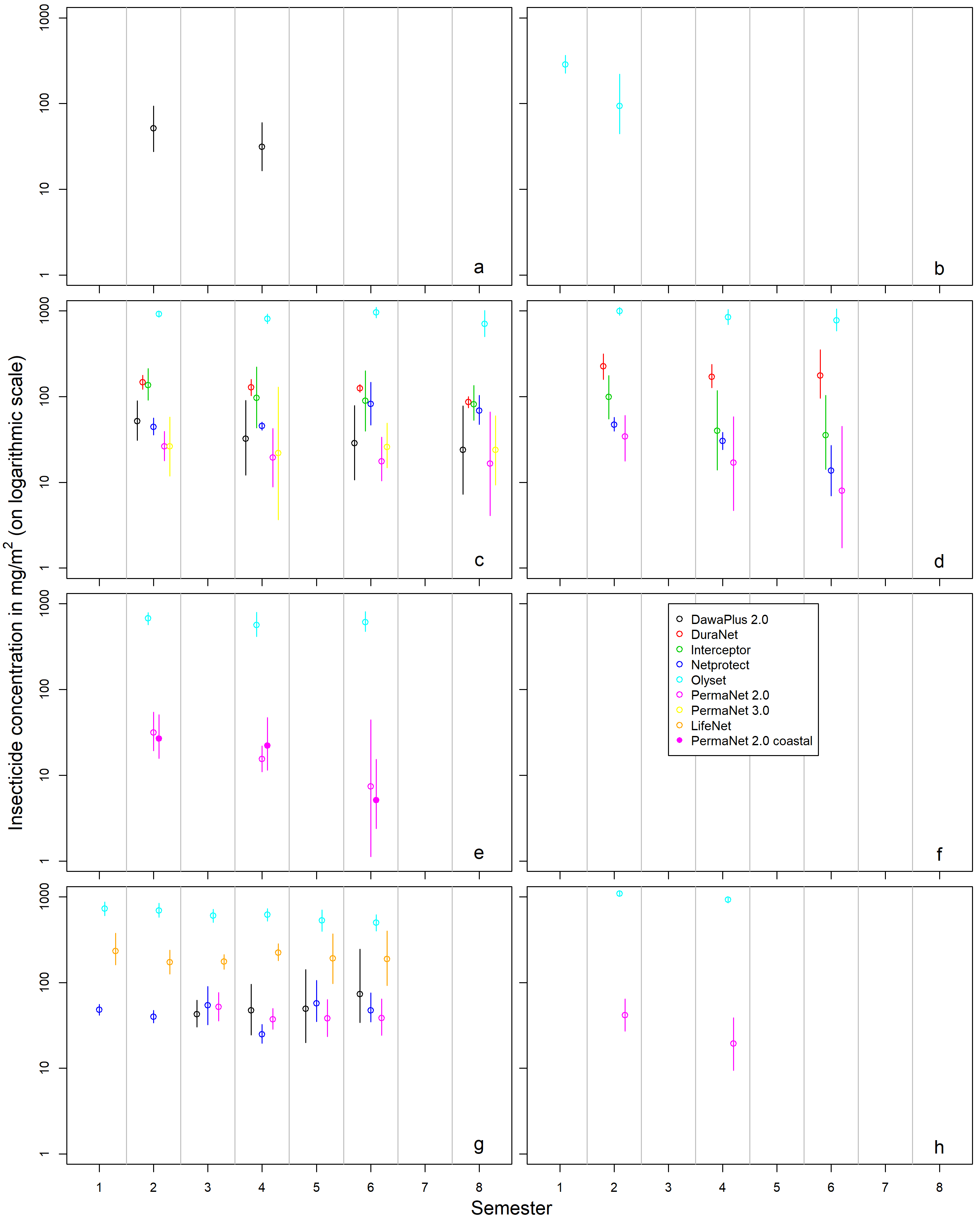


Figure S5. Mean insecticide content (in mg/m^2^) in used LLINs by semester (half year)

a Angola, b Benin, c Kenya, d Malawi, e Mozambique, f legend, g Senegal and h Zambia. Coloured vertical bars show 95% credible intervals.

# Calibration of active ingredient lethality for different net brands.

## Introduction

This section describes the regression models that were fitted to the data for each net brand in each country to relate the proportion of dead mosquitoes in the cone bioassays to the active ingredient content. It further describes the models used to translate the active ingredient content for each net brand in each country onto a scale of lethality in cone bioassays, and to map this onto the corresponding equivalent active ingredient content for a standard net brand (PermaNet 2.0).

## Methods

Logistic regression models were used to model the lethality in the cone bioassays as a function of the log-transformed measurements of active ingredient content. The number of dead mosquitoes out of the number exposed to netting samples in cone tests was regressed against the logarithmically transformed insecticide content (expressed in mg/m^2^) of the tested sample in logistic regressions. Data from control arms (with mosquitoes exposed to untreated netting material) were also included (except for the Zambia study, for which no control data was available, and where the controls from the other four country studies were used as data). Each LLIN sample was matched to a single control based on the date of the assay, but fewer controls were tested than LLIN samples and there was therefore some duplication of controls.

Regressions were carried out in a Bayesian framework with the software JAGS, called from the software platform R [2]. The intercept (mostly governed by the data from controls) was assumed to vary with the survey round number, and was modelled as a Gaussian random effect (with the means Gaussian distributed with common parameters per country study). Models allowing different effects of content in each country were used to fit the relationships shown in Figure S6 to Figure S10 for each of the country studies separately. A model with a common slope across countries for each net brand was used to estimate the ratio of the insecticide coefficient relative to that of PermaNet 2.0 for the analysis of impacts on vectorial capacity (see main paper Figure S5). The ratio of the slope-coefficients of a given net brand X and PermaNet 2.0 thus represents the log odds of a unit increase in log content for net brand X compared to the log odds of a unit increase in log content for PermaNet 2.0. If the ratio is e.g. 0.5, net brand X kills mosquitoes with a probability of half that of PermaNet 2.0 for a given content.

## Results

The relationships between the proportion of dead mosquitoes exposed to netting material in cone bioassays and the chemical content of the netting material for the five selected country studies are shown in scatter plots in Figure S6 to Figure S10. There is considerable variation around the fitted lines, but the fitted relationships are roughly similar for most of the net brands. Table S8 provides the fitted parameters for intercept and slope for each of the net products in the selected country studies, as well as the ratio of the slope parameter relative to PermaNet 2.0.

The ratio of the slope parameter indicated the change in log odds relative to a unit increase in log content of net brand X relative to the log content for PermaNet 2.0. The ratio of the slope parameter was surprisingly similar across all five country studies for Olyset, ranging from 0.29 to 0.38. For DawaPlus, values of 0.89 and 0.91 for Kenya and Senegal were also somewhat similar. For Interceptor, the ratios were 0.55 for Kenya and 0.75 for Malawi, and for DuraNet ratios were 0.98 and 0.72, for Kenya and Malawi, respectively. Netprotect had ratios of 0.66 for Kenya, 0.91 for Malawi, and 0.79 for Senegal. In the model that combined all country studies in a single ratio per LLIN product yielded values that were very close to unity for DawaPlus 2.0, DuraNet, LifeNet and PermaNet 3.0 top panels. Ratios were lower than unity for Interceptor, Netprotect and Olyset, but larger than two for PermaNet 3.0 side panels upper and lower levels.

## Discussion

Differences between net brands in the relationship between active ingredient content and lethality in the cone bioassays are to be expected because of the different active ingredients and the different impregnation technologies. Although the relationships between the proportion of dead mosquitoes in cone bioassays with the insecticide content varies considerably among country studies for the same net product (regression coefficients for e.g. PermaNet 2.0 varying between 1.34 and 2.70, a two-fold difference), the ratio between the coefficients of net products and that of PermaNet 2.0 is more constant across country studies, with a maximum 1.38-fold difference. This justifies the use of these ratios to scale effects of insecticide relative to those of for PermaNet 2.0 for modeling the effects of the nets on mosquito feeding and survival.

Olyset nets stand out as requiring more insecticide content (expressed in mg/m^2^) than the other net products. This is consistent with the higher discriminating dose used to determine susceptibility [3] for permethrin than for deltamethrin or for alpha-cypermethrin. Also, in Olyset nets, insecticide is incorporated inside the filaments, and much of the insecticide may not be available at the surface for mosquitoes to make contact with.

For PermaNet 3.0 in the Kenya country study (Figure S6), disaggregated data were available for lower side panels, upper side panels and top panels, and these are shown in panels g, h and i, respectively. PermaNet 3.0 top panels have a higher deltamethrin target content than PermaNet 3.0 side panels, and also contain piperonyl butoxide (PBO). The fitted relationships are very similar between upper side and lower side panels, and these are both very different from the fitted relationship for top panels (panel i). It is noteworthy that the PermaNet 3.0 side panels seem to be more lethal (with a steeper fitted slope) than PermaNet 2.0 (Figure S6 panel a), or any of the other deltamethrin or alpha-cypermethrin containing nets. In the simulations of entomological effects of PermaNet 3.0, the insecticide content in the lower-side panels (and the corresponding scaling factors) were therefore used.

These results allow scaling of effects of active ingredient content for all net products in all country studies, except for Benin, where a different measurement technique was used for the active ingredient content of Olyset nets. These measurements were converted to units of mg/m^2^, by assuming the permethrin content in new nets to be the same as in Mozambique.

## Figures


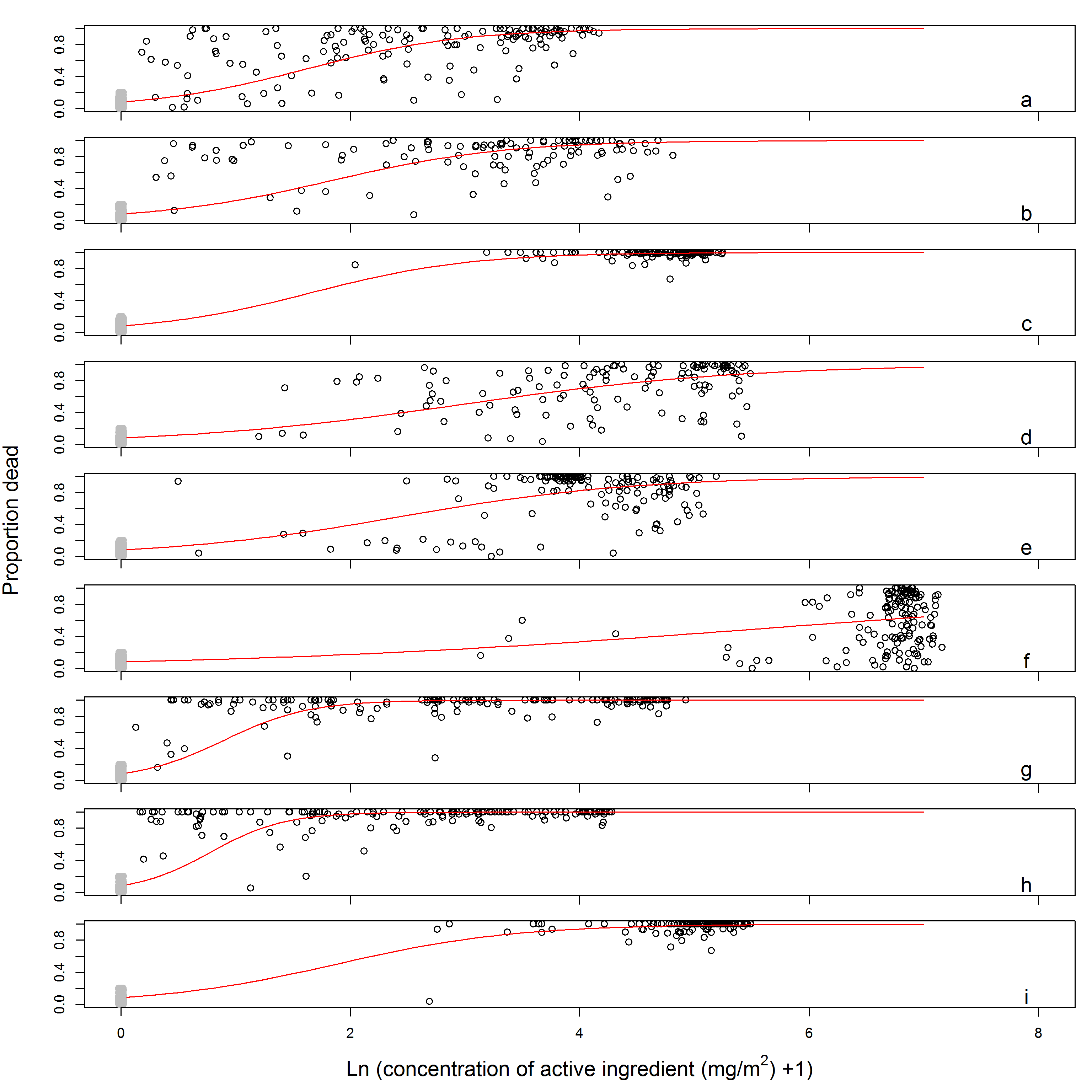
**Figure S6. Kenya relationship between proportion dead and active ingredient content in cone bioassays**

Legend: Net products **a** PermaNet 2.0; **b** DawaPlus 2.0; **c** DuraNet **d** Interceptor; **e** Netprotect; **f** Olyset; **g** PermaNet 3.0 upper sample of side panels; **h** PermaNet 3.0 lower sample of side panels; **i** PermaNet 3.0 sample of top panels. The red line indicates the fitted relationship, the black circles the measurements. The grey circles (jittered on the horizontal axis) give the proportion dead in control assays with untreated netting material.


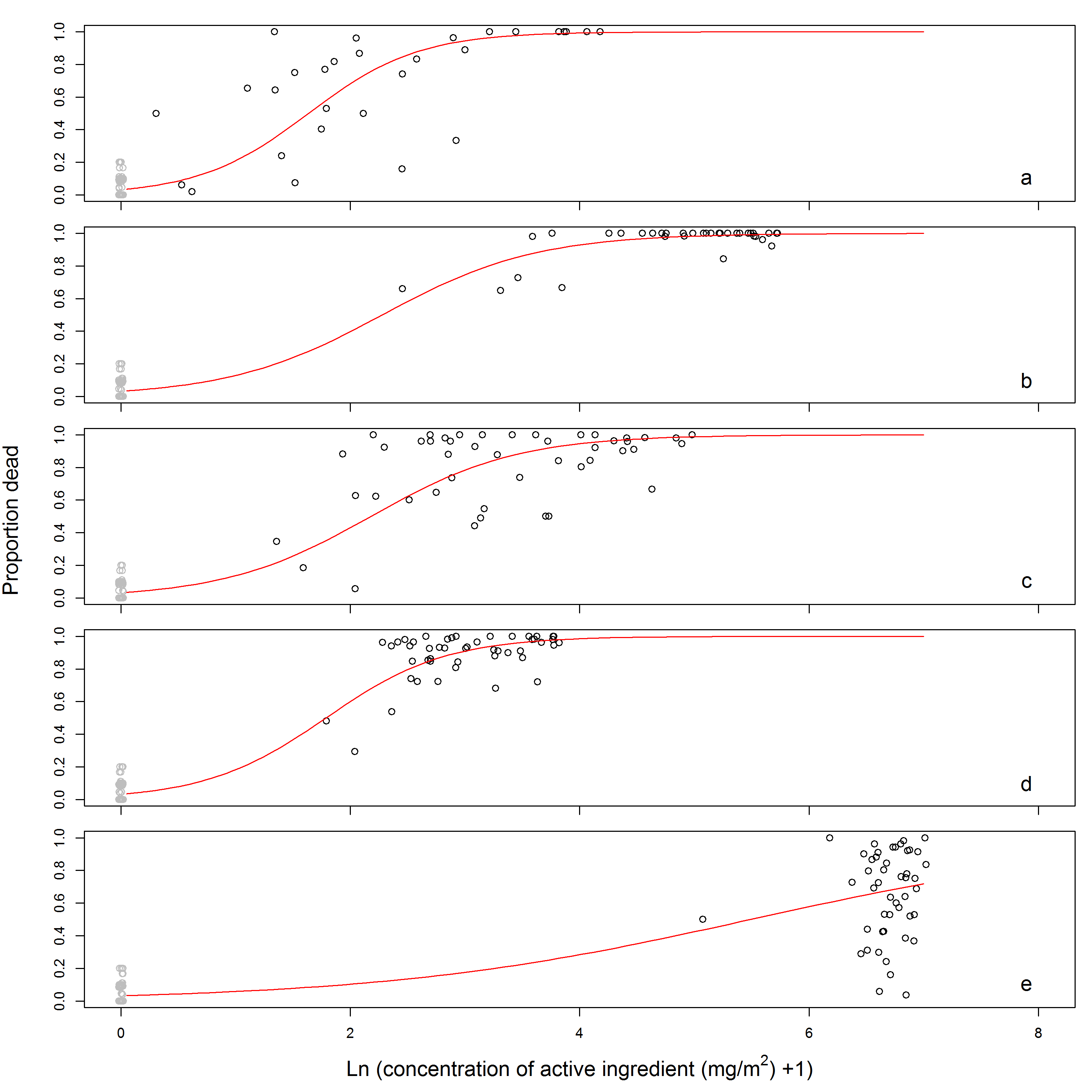


**Figure S7. Malawi relationship between proportion dead and active ingredient content in cone bioassays**

Legend: Net products **a** PermaNet 2.0; **b** DuraNet; **c** Interceptor; **d** Netprotect; **e** Olyset. The red line indicates the fitted relationship, the black circles the measurements. The grey circles (jittered on the horizontal axis) give the proportion dead in control assays with untreated netting material.


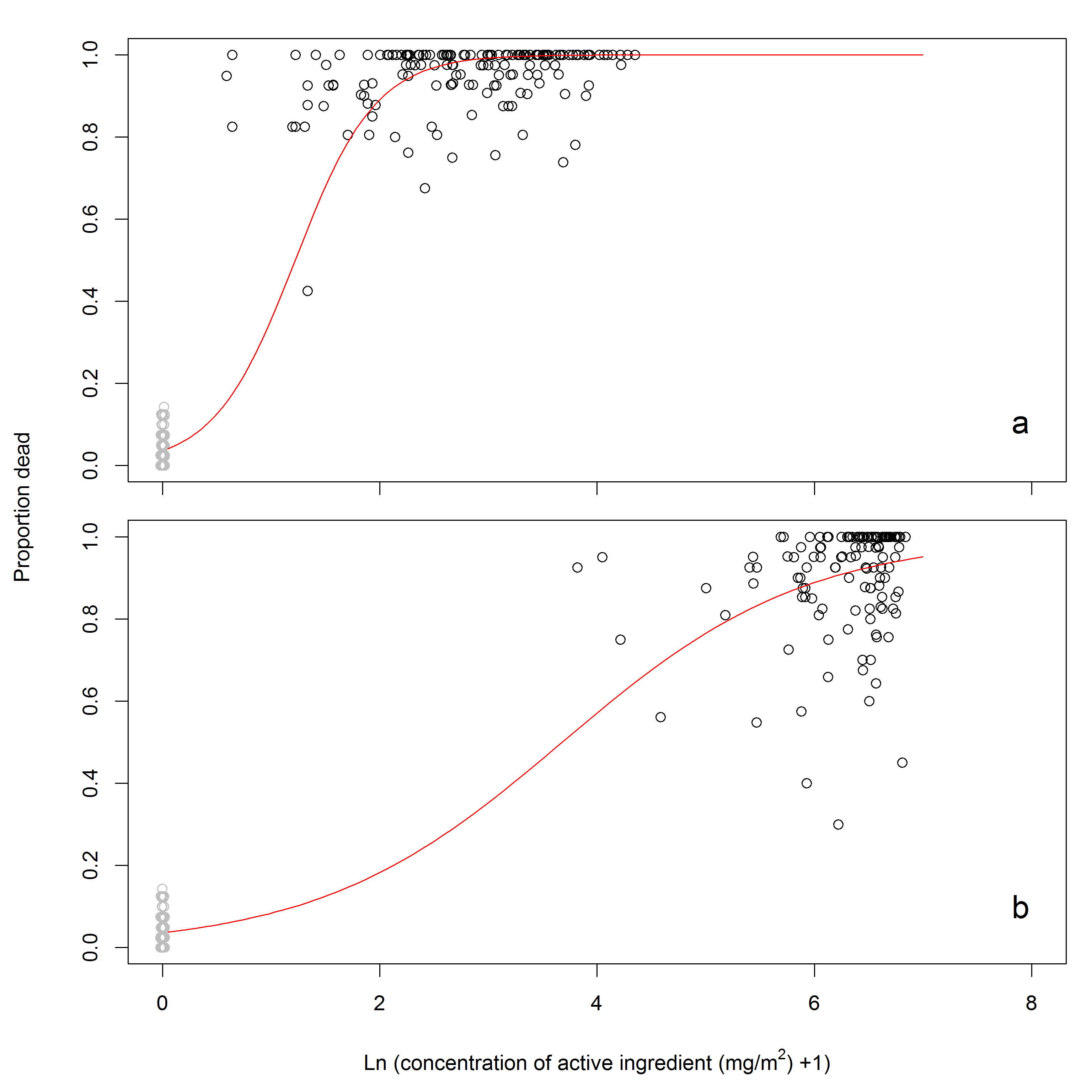


**Figure S8. Mozambique relationship between proportion dead and active ingredient content in cone bioassays**

Legend: Net products **a** PermaNet 2.0; **b** Olyset. The red line indicates the fitted relationship, the black circles the measurements. The grey circles (jittered on the horizontal axis) give the proportion dead in control assays with untreated netting material.


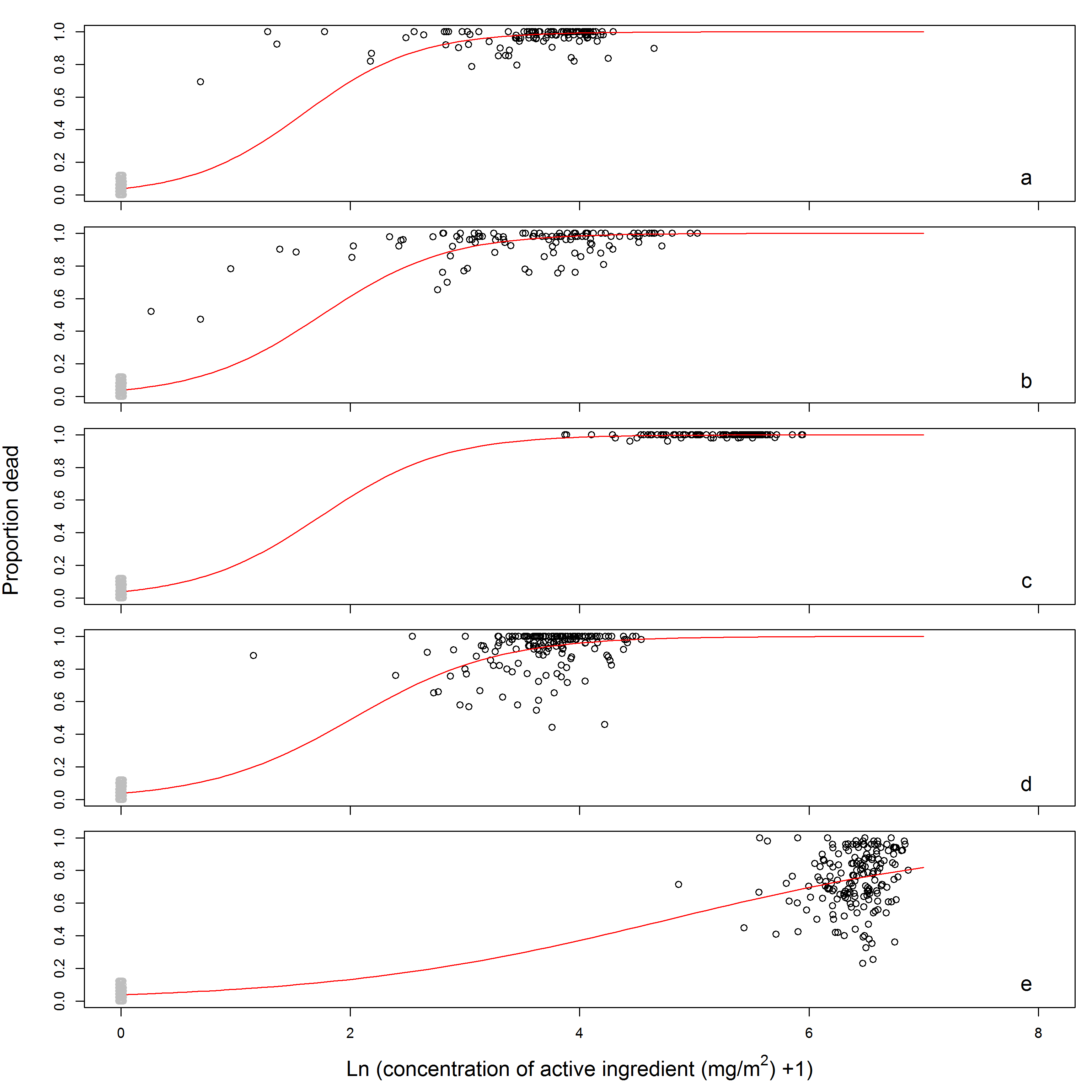


**Figure S9. Senegal relationship between proportion dead and active ingredient content in cone bioassays**

Legend: Net products **a** PermaNet 2.0; **b** DawaPlus 2.0; **c** LifeNet; **d** Netprotect; **e** Olyset. The red line indicates the fitted relationship, the black circles the measurements. The grey circles (jittered on the horizontal axis) give the proportion dead in control assays with untreated netting material.


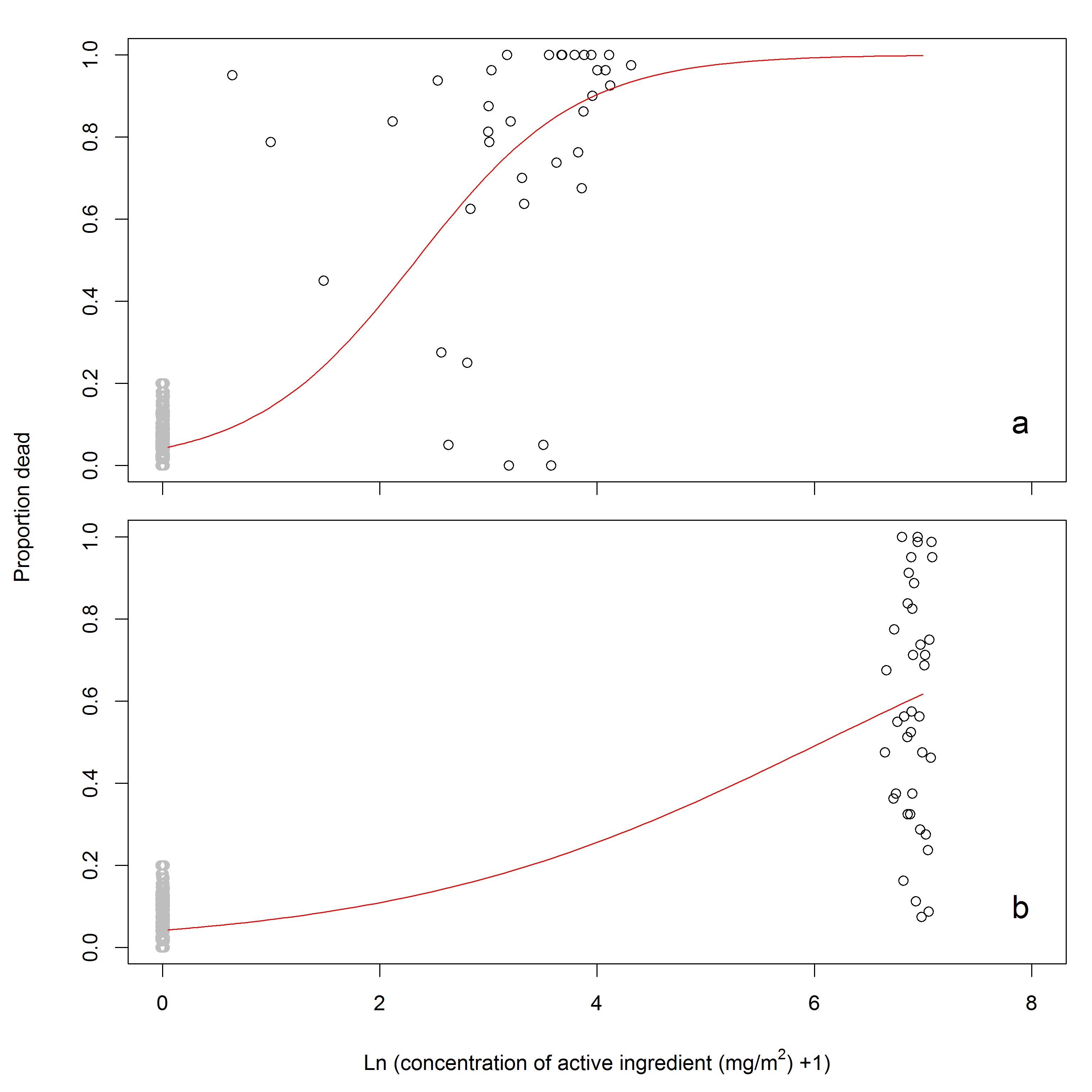


**Figure S10. Zambia relationship between proportion dead and active ingredient content in cone bioassays**

Legend: Net products **a** PermaNet 2.0; **b** Olyset. The red line indicates the fitted relationship, the black circles the measurements. The grey circles (jittered on the horizontal axis) give the proportion dead in control assays of Kenya, Malawi, Mozambique and Senegal with untreated netting material.

**Table S8: Fitted parameter values (median and 95% credible intervals) for the modeled relationship between 24 hour mortality in cone bioassays and insecticide content in the netting material**

|  |  | Country study |  |  |  |  |  |
| --- | --- | --- | --- | --- | --- | --- | --- |
| Parameter | Net product | Kenya | Malawi | Mozambique | Senegal | Zambia | Combined model |
| Intercept | all products | -2.41 (-3.01 – -1.77) | -3.41 (-6.56 – -0.16) | -3.29 (-4.66 – -1.83) | -3.24 (-3.65 – -2.80) | -3.13 (-31.14 – 28.61) |  |
| Slope | PermaNet 2.0 | 1.48 (1.45 – 1.51) | 2.09 (1.99 – 2.17) | 2.70 (2.61 – 2.78) | 2.03 (1.98 – 2.09) | 1.34 (1.32 – 1.36) |  |
|  | DawaPlus 2.0 | 1.31 (1.28 – 1.34) |  |  | 1.85 (1.80 – 1.89) |  |  |
|  | DuraNet | 1.45 (1.41 – 1.49) | 1.50 (1.44 – 1.56) |  |  |  |  |
|  | Interceptor | 0.81 (0.79 – 0.83) | 1.57 (1.51 – 1.62) |  |  |  |  |
|  | Netprotect | 0.98 (0.97 – 1.00) | 1.91 (1.85 – 1.96) |  | 1.60 (1.57 – 1.62) |  |  |
|  | Olyset | 0.43 (0.42 – 0.44) | 0.62 (0.60 – 0.64) | 0.89 (0.87 – 0.92) | 0.68 (0.67 – 0.69) | 0.52 (0.51 – 0.52) |  |
|  | PermaNet 3.0 lower side | 2.70 (2.54 – 2.78) |  |  |  |  |  |
|  | PermaNet 3.0 upper side | 3.09 (2.91 – 3.20) |  |  |  |  |  |
|  | PermaNet 3.0 top | 1.28 (1.25 – 1.33) |  |  |  |  |  |
|  | LifeNet |  |  |  | 1.87 (1.72 – 1.94) |  |  |
| Slope Ratio | PermaNet 2.0 | 1 | 1 | 1 | 1 | 1 |  |
|  | DawaPlus 2.0 | 0.89 (0.86 – 0.91) |  |  | 0.91 (0.88 – 0.94) |  | 1.00 (0.98 – 1.01) |
|  | DuraNet | 0.98 (0.94 – 1.02) | 0.72 (0.68 – 0.76) |  |  |  | 1.04 (1.02 – 1.07) |
|  | Interceptor | 0.55 (0.53 – 0.56) | 0.75 (0.72 – 0.78) |  |  |  | 0.68 (0.67 – 0.69) |
|  | Netprotect | 0.66 (0.65 – 0.68) | 0.91 (0.88 – 0.95) |  | 0.79 (0.76 – 0.81) |  | 0.83 (0.82 – 0.84) |
|  | Olyset | 0.29 (0.28 – 0.30) | 0.30 (0.29 – 0.31) | 0.33 (0.32 – 0.34) | 0.33 (0.32 – 0.34) | 0.38 (0.38 – 0.39) | 0.35 (0.35 – 0.36) |
|  | PermaNet 3.0 lower side | 1.82 (1.72 – 1.89) |  |  |  |  | 2.32 (2.24 – 2.40) |
|  | PermaNet 3.0 upper side | 2.09 (1.98 – 2.17) |  |  |  |  | 2.09 (2.02 – 2.16) |
|  | PermaNet 3.0 top | 0.87 (0.84 – 0.90) |  |  |  |  | 1.02 (0.99 – 1.05) |
|  | LifeNet |  |  |  | 0.92 (0.85 – 0.96) |  | 0.99 (0.94 – 1.05) |

# Parameterisation of LLIN effects as functions of their physical integrity and chemical content

## Introduction

For the simulation of the impact of LLIN-use, the entomological model requires parameterization of the effects of LLINs on (i) the proportion of mosquitoes averted, which can be split into two components: the proportion entering houses (as a fraction is possibly deterred from entering by chemicals emanating from the houses), which is purely a function of insecticide content, (ii) the proportion attacking out of those that entered houses (a function of chemical content and holed surface area of the LLIN) and (iii) the proportion killed before feeding (pre-prandial killing), and proportion killed after feeding (post-prandial killing). This document describes functions estimated from experimental hut data used to model these proportions.

## Methods

### Deterrence from entering huts

The effects of new and 20 times washed PermaNet 2.0 LLINs on deterrence from entering houses was previously estimated with experimental huts on voluntarily entering *An. gambiae* s.l. mosquitoes in several locations [4]. Deterrence from entry was estimated as one minus the ratio of the total number of mosquitoes found inside huts with an LLIN and the number found inside huts with an untreated net.

The proportion entering as a function of logarithmically transformed insecticide content was estimated in a Bayesian binomial regression model with logit link function, and a fixed intercept of logit(0.99) (assuming that 99% of mosquitoes enter houses without any emanating insecticide), instead of an assumption of 100% entering without insecticide, which would give problems with logit transformation. The intercept was fixed, because there were only two data points available. The insecticide content was logarithmically transformed because it was assumed that the effect saturates with increasing insecticide content.

For the susceptible *An. gambiae* s.l. population Zeneti (predominantly *An. gambiae* s.s.) in Tanzania, the estimated regression coefficient was -0.844 (95% CI -0.878 – -0.810). This relationship is shown in the main paper and in Figure S9. For *An. gambiae* s.l. mosquitoes in Akron, Benin, with a strong resistance profile, the estimated regression coefficient was -0.986 (95% CI -1.054 – -0.916).

### Probabilities of attacking, pre-prandial and post-prandial killing for PermaNet 2.0

Data from Randriamaherijaona and colleagues [5] were used to estimate the probabilities of attacking, pre-prandial and post-prandial killing of PermaNet 2.0 on the susceptible *An. gambiae* s.s. Kisumu strain and on the resistant *An. gambiae* s.l. in Akron, depending on holed surface area and insecticide content. For the latter, only two content were available: untreated nets and new PermaNet 2.0 nets (presumed to have the product target deltamethrin content of 55 mg/m^2^).

The effects of logarithmically transformed insecticide content and logarithmically transformed holed surface area on the outcomes were estimated in a Bayesian binomial regression model with logit link function and included an interaction term for holed surface area and insecticide content. For the estimation of the post-prandial killing effect, the intercept was fixed at logit(0.01). Results of the logistic regression models are shown in Table S9, and the relationships are plotted in Figure S12 to Figure S14. Figure S15 illustrates the overall insecticidal effect [6], Figure S16 illustrates the personal protection, and Figure S17, Figure S18 and Figure S19 show the proportional impact on VC in hypothetical situations where 80%, 40%, or 20% of the population, respectively, use identical LLINs.

###

### Scaling insecticide coefficients for LLIN products other than PermaNet 2.0

For different LLIN brands, the log-insecticide content was multiplied with scaling factors dependent on the relationship between insecticide content and mortality in cone. In the simulations with PermaNet 3.0, the insecticide content in the lower-side panels (and the corresponding scaling factors) were used.

## Results

The parameters of the logistic regression models are given in Table S9, and the fitted regressions are plotted in Figure S11 to Figure S19 and in the main paper as Figure 6, in each case separately for pyrethroid susceptible and pyrethroid resistant mosquitoes. Figure S15 shows the overall insecticidal effect [6] which exhibits a peak at an insecticide content of about 150 mg/m^2^, after which the effect declines. This is because the deterrence from hut entry (Figure S11) increasingly prevents mosquitoes from being killed with increasing content. Figure S16 illustrates the personal protection, and Figure S17, Figure S18 and Figure S19 the proportional impact on VC in hypothetical situations where 80%, 40% or 20% of the population, respectively, use identical LLINs. At 80% coverage, the plot of impact on VC mostly resembles that of the plot of personal protection, while at 20% coverage, the plot of impact on VC is more similar to that of the overall insecticidal effect.

## Tables and figures of parameters used in the modelling of vectorial capacity

####

#### Table S9: LLIN effect parameter values

| Parameter | Source of value | Value for susceptible *Anopheles gambiae* (Zeneti) | Value for resistant *Anopheles gambiae* (Akron) |
| --- | --- | --- | --- |
| $\beta_{0,ent}$ | Assumed | logit(0.99) | logit(0.99) |
| $\beta_{1,ent}$ | Estimated by MCMC | -0.844 | -0.986 |
| $\beta_{0,att}$ | Estimated by MCMC | -2.555 | -4.069 |
| $\beta_{1,att}$ | Estimated by MCMC | 0.379 | 0.541 |
| $\beta_{2,att}$ | Estimated by MCMC | 1.807 | 0.807 |
| $\beta_{3,att}$ | Estimated by MCMC | -0.078 | -0.101 |
| $\beta_{0,B\mu}$ | Estimated by MCMC | -1.823 | -4.840 |
| $\beta_{1,B\mu}$ | Estimated by MCMC | -0.235 | -0.128 |
| $\beta_{2,B\mu}$ | Estimated by MCMC | 2.245 | 2.325 |
| $\beta_{3,B\mu}$ | Estimated by MCMC | -0.094 | -0.113 |
| $\beta_{0,C\mu}$ | Assumed | logit(0.01) | logit(0.01) |
| $\beta_{1,C\mu}$ | Estimated by MCMC | -0.370 | -0.114 |
| $\beta_{2,C\mu}$ | Estimated by MCMC | 2.816 | 1.303 |
| $\beta_{3,C\mu}$ | Estimated by MCMC | 0 | 0 |
| $H_{max}$ | Total surface area of LLIN (cm^2^) | 192000 | 192000 |

Legend: see main paper

####
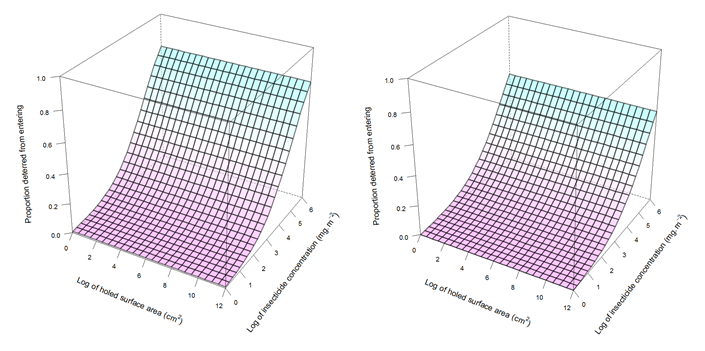
Figure S11.The proportion of *An. gambiae* s.l. being deterred from entering huts depending on the insecticide content in the PermaNet 2.0 LLIN in the hut.


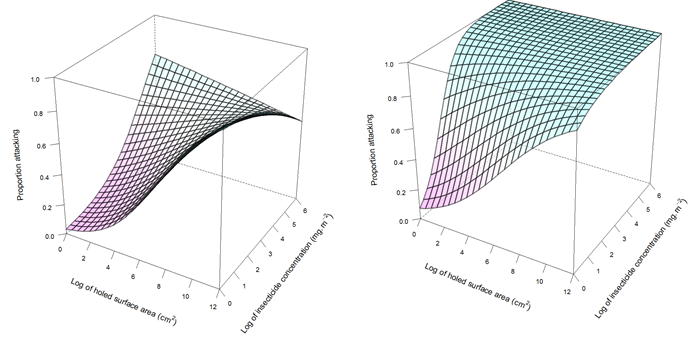
**a**) resistant *An. gambiae* s.l. Akron population; **b**) susceptible *An. gambiae* s.l. Zeneti population. The deterrence is independent of the holed surface area in the LLIN, (shown as a 3D plot for consistency with subsequent figures

#### Figure S12. The proportion of *An. gambiae* s.l. attacking (resulting in feeding and / or dying) inside huts with PermaNet 2.0 LLIN depending on the insecticide content and holed surface area in the LLIN.

**
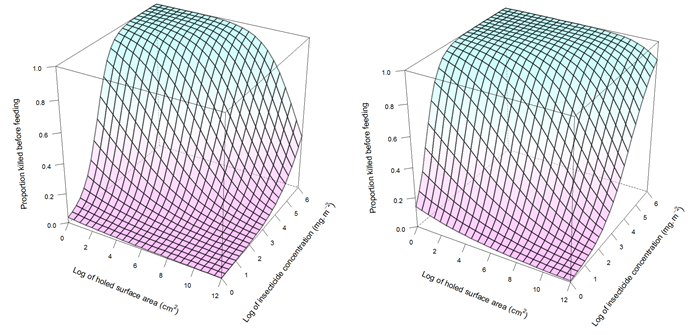
a**) resistant *An. gambiae* s.l. Akron population; **b**) susceptible *An. gambiae* s.l. Zeneti population.

#### Figure S13. The proportion of *An. gambiae* s.l. being killed before feeding depending on the insecticide content and holed surface area in the PermaNet 2.0 LLIN

**a**) resistant *An. gambiae* s.l. Akron population; **b**) susceptible *An. gambiae* s.l. Zeneti population.


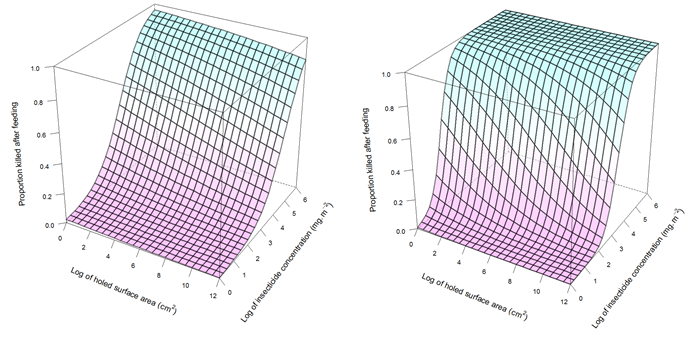
F**igure S14. The proportion of An. gambiae s.l. being killed after feeding depending on the insecticide content and holed surface area in the PermaNet 2.0 LLIN**


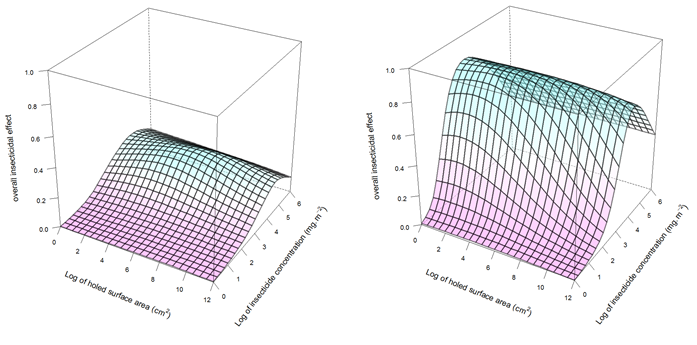
**a**) resistant *An. gambiae* s.l. Akron population; **b**) susceptible *An. gambiae* s.l. Zeneti population.

Figure S15. The overall insecticidal effect of a PermaNet 2.0 LLIN on *An. gambiae* s.l. depending on the insecticide content and holed surface area


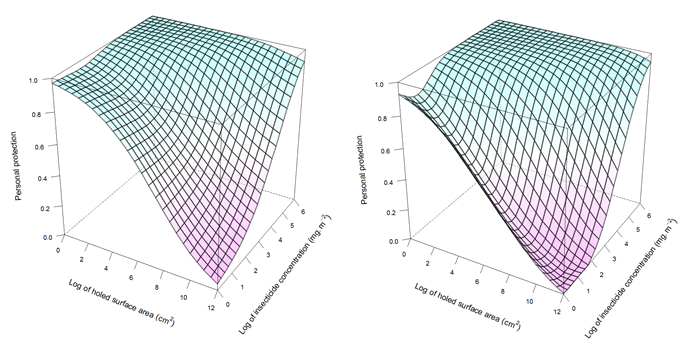
**a**) resistant *An. gambiae* s.l. Akron population; **b**) susceptible *An. gambiae* s.l. Zeneti population.

#### Figure S16. The personal protection provided by a PermaNet 2.0 LLIN against *An. gambiae* s.l. bites depending on the insecticide content and holed surface area

**a**) resistant *An. gambiae* s.l. Akron population; **b**) susceptible *An. gambiae* s.l. Zeneti population.

**
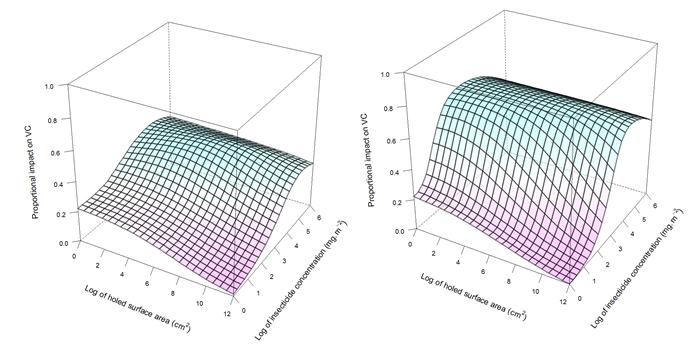
****Figure S17. The proportional impact on vectorial capacity of An. gambiae s.l. depending on the insecticide content and holed surface area in PermaNet 2.0 LLINs in a population with a use coverage of 80% of identical LLINs**

**a**) resistant *An. gambiae* s.l. Akron population; **b**) susceptible *An. gambiae* s.l. Zeneti population.


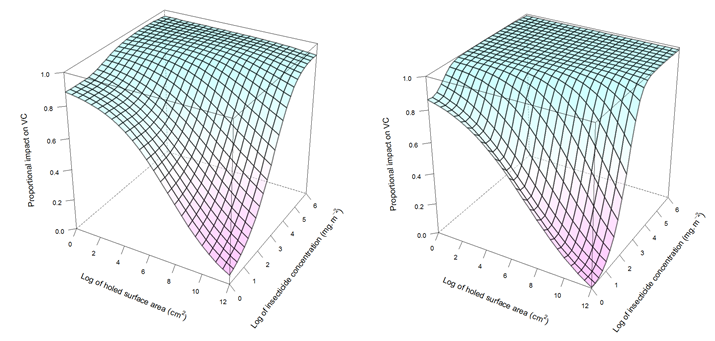


Figure S18. The proportional impact on vectorial capacity of *An. gambiae* s.l. depending on the insecticide content and holed surface area in PermaNet 2.0 LLINs in a population with a use coverage of 40% of identical LLINs


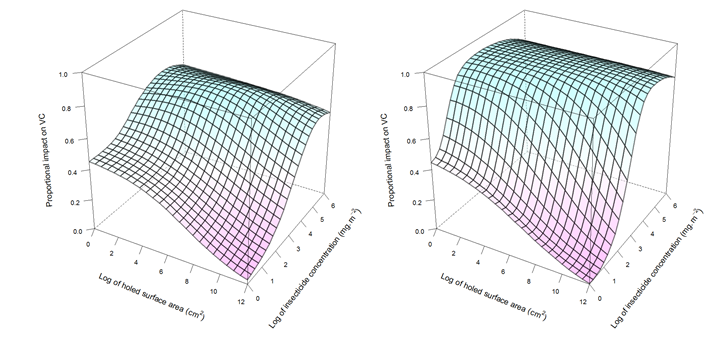
**a**) resistant *An. gambiae* s.l. Akron population; **b**) susceptible *An. gambiae* s.l. Zeneti population.

Figure S19. The proportional impact on vectorial capacity of *An. gambiae* s.l. depending on the insecticide content and holed surface area in PermaNet 2.0 LLINs in a population with a use coverage of 20% of identical LLINs

**a**) resistant *An. gambiae* s.l. Akron population; **b**) susceptible *An. gambiae* s.l. Zeneti population.

# Simulation of effects on vectorial capacity

## Methods

### Simulation of insecticide content and holed area

For each LLIN brand and for each survey, a Weibull statistical distribution model was fitted to the chemical content in the LLINs, where the logarithmically transformed scale parameter of the Weibull distribution depended linearly on the logarithmically transformed holed surface area in the LLIN as well as on whether the LLIN was used in the night prior to the interview or not. In the same model, the log-holed surface area in the LLIN was modelled to be normally distributed with the mean and precision depending on the use status. If applicable, the holed surface area was censored for those observations where LLINs had more holes than measured (e.g. when seam failures were recorded or where parts of panels of LLINs were missing). The use-status was modelled using a Bernoulli distribution. The latter was done to estimate the use status for observations of LLINs where use-status was missing. Also in the same model, a prediction was made of holed surface area in used LLINs, and, conditional on the holed surface area, a prediction was made of the insecticide content. Models were fitted and predictions were made using five chains with 2,000 iterations each (and a burn-in of 10 iterations). The algorithm thus yielded, for each of the LLIN brand and survey combinations, 10,000 samples of LLINs with the properties of LLINs that had been used the night prior to the interview and with a chemical content that was correlated to the holed area in the LLIN. The model is provided in the Supplementary Information: Bayesian Model Specification

### Vectorial capacity

For each LLIN brand, survey, and country, a human population of 1,000 individuals was simulated, and the proportion of these individuals using an LLIN was sampled randomly without replacement from the joint posterior distribution of the estimates of net survival and use (see Supplementary Information Bayesian model specification). The simulated nets were assigned physical and chemical status sampled randomly without replacement from joint posterior distributions of the estimates of physical and chemical status for the nets used in the night prior to the interview (see main text). These values of use, physical and chemical status, were used to parameterise the entomological model [7] which was then implemented in the R software platform, using previously published parameters for model of the feeding cycle for *Anopheles gambiae* s.s. in the absence of intervention[8, 9].

### Proportion of vectorial capacity averted

The vectorial capacity (VC) in these scenarios was compared to the VC in a scenario without any LLINs, and the LLIN impact on VC was calculated as the proportion of VC in the absence of LLINs that was averted by LLINs. The calculations were carried out for each survey round, and for each net brand in each country. Figure S20 illustrates the method of calculating the proportion of residual VC that can be averted in scenarios graphically for Angola, with the hypothetical scenario that all LLINs present in the population are being used. In panel **a**, the averted VC is shown as calculated with the observed LLIN durability properties. In panel **b**, a similar quantity is calculated for a scenario where all LLINs present in the population are being used. Finally, in panel **c**, the proportion of the residual proportion (one minus the values in panel **a**) that is additionally averted by the alternative hypothetical scenario is shown. In this example, the observed LLIN population averts about 84% of VC after one year, and about 54% after two years. If all LLINs were being used, this would be about 95% and 81%, respectively. Thus, about 67% [100-100*(100-95)/(100-84)] and 57%, respectively, of residual VC could be averted if all LLINs that are present in the population are being used.

It should be noted that it might not be possible to prevent all VC with LLINs, as even with 100% use-coverage with new intact LLINs, there might be residual VC due to outdoor biting.

### Sensitivity of the vectorial capacity averted to the different components of LLIN durability

The effect on vectorial capacity was calculated for a series of counterfactual scenarios in which different components of net decay (attrition, use, insecticide decay and decay of physical integrity) were precluded

(i) to estimate the importance of attrition, survival of nets was set to unity in the counterfactual. Other properties were as in the field surveys.

(ii) to estimate the importance of non-use, the counterfactual the proportion of LLINs that were used was set to unity in the counterfactual, while the condition of the LLINs was that of LLINs actually used the night prior to the survey.

(iii) to estimate of the importance of decay in insecticide content, all LLINs in the counterfactual had target insecticide content (simulating ‘chemically everlasting’ LLINs).

(iv) to estimate the importance of physical decay the counterfactual comprised only intact nets.

## Results

The proportion of the VC that was averted by study LLINs by LLIN brand, semester, and country are shown in Figure and Table S7 for susceptible *An. gambiae* s.l. and in Figure and Table S6 for resistant *An. gambiae* s.l. Similar plots for hypothetical scenarios where LLINs were intact (but otherwise had the same properties as the LLINs in the studies), had their target insecticide content (idem), were all being used (idem), or did not attrite (idem) are shown for resistant *An. gambiae* s.l. in Figure S23 to Figure S26. Most country studies showed that the impact on VC declined over time, with some exceptions: notably LifeNet in Senegal increased impact between semesters 1 and 2, and also between semesters 5 and 6. The most frequently tested LLIN brands were Olyset and PermaNet 2.0. PermaNet 2.0 outperformed Olyset in Kenya, Malawi, Mozambique, Senegal and Zambia, but the ranking was reversed in semesters 5 and 6 in Senegal. There were large differences for the same LLIN brands between country studies: e.g. after one year, Olyset averted 95, 85, 76, 72, 65 and 40% of VC in Zambia, Malawi, Benin, Kenya, Senegal and Mozambique, respectively.

Tables 6 and 7 in the main paper summarise for susceptible and resistant *An. gambiae* s.l., respectively, over all LLIN brands and surveys, for each country study, the proportion of the residual VC (that remains when the study-observed LLINs are being used) that was averted in the scenarios where LLINs were intact (but otherwise had the same properties as the LLINs in the studies), contained their target insecticide content (idem), were all being used (idem), or did not attrite (idem). With the hypothetical scenario where all existing LLINs were used, generally the greatest proportion of the residual VC could be averted, with averages over all LLIN brands and surveys ranging from 57% in Zambia to 86% in Senegal, assuming that *An. gambiae* s.l. was relatively resistant. Only in Zambia, the proportion averted was slightly larger with the hypothetical scenario where all LLINs were physically intact. For the other country studies, rendering LLINs intact did little to reduce residual VC. Preventing the insecticide from decaying improved LLIN impact more than preventing physical damage in all country studies except Zambia, but the impact was much lower than with maximizing use. Preventing attrition (maximizing LLIN survival) showed some impact in Angola, Kenya and Malawi, but the impact was less than if use were maximized. When *An. gambiae* s.l. was presumed to be susceptible, this generally increased the proportion of the residual VC that could be averted by maximizing use, maximizing survival, or rendering nets intact, compared to the scenarios with resistant mosquitoes, with a few exceptions (a slight reduction for the ‘intact’ and maximum survival scenarios in Zambia). However, for the target content scenarios, the proportion of residual VC that was prevented with susceptible vectors was smaller in Angola, Kenya, Malawi, Mozambique and Zambia, while slightly higher in Senegal, and much higher in Benin (even outperforming the maximum use scenario).

The disaggregated results (by LLIN brand and by survey) of impact of hypothetical improvements to LLINs are shown in Figure S27 to Figure S30 for resistant *An. gambiae* s.l. and in Table S10 to Table S19 for both susceptible and resistant *An. gambiae* s.l.. There were no common temporal trends in the proportion of residual transmission averted by preventing physical damage (Figure S27), or by preventing insecticide decay (Figure S28). However, the proportion of residual VC averted by maximizing use generally reduced with time, with some exceptions in Senegal (all LLIN brands except Olyset) and Zambia (Olyset, with resistant *An. gambiae* s.l. only) (Figure S29). In contrast, preventing attrition showed increasing trends in impact in Angola, Benin, Kenya, Malawi and Mozambique (Figure S30).

Although 95% credible intervals (CIs) of additional impact estimates of preventing physical damage in individual LLIN brands at specific surveys mostly included zero, preventing physical damage (Figure S27) showed significant impact on VC with resistant mosquitoes for Olyset in Benin (at 12 months), in Malawi (at 12, 24 and 36 months), Senegal (at 6, 12, 18, 24, 30 and 36 months) and Zambia (at 12 and 24 months); for PermaNet 2.0 in Kenya (at 24 months), Malawi (at 36 months) and Zambia (at 24 months); for Interceptor in Kenya and Malawi (both at 24 months); and Netprotect in Malawi (at 24 and 36 months). With susceptible mosquitoes, this was no longer the case for Olyset in Malawi and Senegal at 12 months, but included PermaNet 2.0 in Kenya at 36 months.

Preventing insecticide decay (Figure S28) showed significant improvements for Olyset in Benin (at 6 months for susceptible mosquitoes and at 12 months for both susceptible and resistant mosquitoes) and in Senegal (at 18 months); for PermaNet 2.0 in Kenya (at 24 and 36 months for both susceptible and resistant mosquitoes, and at 48 months for resistant mosquitoes), in Malawi (at 24 and 36 months), in Mozambique (at 36 months for resistant mosquitoes) and Zambia (at 24 months); for Interceptor in Kenya (at 24, 36 and 48 months for resistant mosquitoes, but only at 24 months for susceptible mosquitoes) and in Malawi (at 12, 24 and 36 months for resistant mosquitoes, but only at 24 and 36 months for susceptible mosquitoes); for Netprotect in Malawi (at 24 and 36 months for resistant mosquitoes, but only at 36 months for susceptible mosquitoes); for DawaPlus 2.0 in Kenya, but only for resistant mosquitoes (at 24, 36 and 48 months).

In Kenya, with susceptible mosquitoes, the effect of preventing insecticide decay was significantly negative for DuraNet at 24 and 32 months and PermaNet 3.0 (all instances). With resistant mosquitoes, although not significant in individual surveys, in Kenya, with data combined over all surveys (at 12, 24, 36 and 48 months), the effect of preventing insecticide decay was also significantly negative for DuraNet and PermaNet 3.0. This can be explained by the relatively high insecticide content in these LLINs, combined with a scaling factor above unity (Table 5 in the main paper). At target insecticide content, these LLINs have less impact on VC than at intermediate content due to the modelled effect on deterrence from hut entry which prevents mosquitoes from being killed with increasing content. This is illustrated in Figure S11 and Figure S12. Because of this negative effect, preventing physical decay in DuraNet and PermaNet 3.0 LLINs was more beneficial than preventing insecticide decay in these LLINs. Also for Olyset, preventing physical decay was more beneficial than preventing insecticide decay, except in Benin at 6 months and Kenya at 12 months. With resistant mosquitoes, for all other LLINs (DawaPlus 2.0, Interceptor, LifeNet, Netprotect and PermaNet 2.0), preventing insecticide decay was more beneficial than preventing physical decay. With susceptible mosquitoes however, for these LLINs, the relative ranking varied strongly depending on the survey.

Maximizing use (Figure S29) showed significant improvements for all instances except Interceptor in Kenya at 48 months and Interceptor at 36 months (only for susceptible mosquitoes). Preventing attrition (maximizing survival, in Figure S30) showed significant improvements for Olyset in Benin (at 12 months), in Malawi (at 36 months), and in Senegal (at 30 and 36 months); for PermaNet 2.0 in Kenya (at 36 and 48 months), in Malawi (at 36 months); for PermaNet 3.0 in Kenya (at 48 months); for Interceptor in Kenya (at 48 months) and in Malawi (at 24 and 36 months); for Netprotect in Kenya (at 48 months) and in Malawi (at 24 and 36 months); for DawaPlus 2.0 in Angola (at 12 and 24 months) and Kenya (at 48 months); for DuraNet in Malawi (at 36 months).

## Discussion

A discussion of the main aspects of the estimation of vectorial capacity is provided in the main paper. This text elaborates on methodological issues that are only briefly summarised there. Specifically:

**Analysis of individual nets as isolated units**: The LLIN durability country studies generally followed individual LLINs from distribution, or shortly thereafter, until lost to follow up, and were “LLIN centric”: There is little^[[1]](#footnote-1)^ or no information available on the condition (in terms of holes and insecticide content) and use of other, non-study nets in the population, and how these interacted with the use of study LLINs. Alongside study LLINs, non-study nets have effect on the VC (in a transmission setting, the effect of LLINs on VC depends non-linearly on the LLIN use-coverage, i.e. the proportion of the population that uses an LLIN), and ideally, the impact on VC should be estimated over time in a realistic setting with repeated mass-distributions. However, since there is little information on the actual use and condition of non-study nets, it was not possible to include these effects in the models. Non-study nets are likely to influence the attrition and use of study nets. For example, in the Kenya country study, the use of study nets was increasing during the first two years post distribution, possibly because other nets were still in use and in the process of being replaced by study nets. At the end of the study period, study LLIN survival and use declined, probably at least in part due to them being replaced by new non-study nets being distributed.

**Parameterization of counterfactual without attrition:** Ideally, a counterfactual without attrition should be modeled where the population access coverage is kept at 100%,. This is theoretically achievable if BCC campaigns could convince everyone to retain their LLINs, while keeping use levels unchanged. Because of the phenomenon that the average condition of remaining LLINs may improve over time as the worst LLINs attrite, simply keeping the average LLIN condition the same as that measured in the remaining LLIN population would overestimate the impact of eliminating attrition. Instead, the condition of the otherwise attrited LLINs should be modelled to be worsening over time. However, there were no data available on the condition of these attrited LLINs. If repeated measures of insecticide and holes in LLINs were available, these could be used to estimate the condition of attrited LLINs by extrapolation of decay curves (with a censoring approach). Benin and Zambia country studies measured holes repeatedly in the same LLINs, and it might be possible to establish physical decay curves for individual LLINs, and extrapolate these. The other country studies measured holes in LLINs retrieved from the population. None of the studies had repeated measures of insecticide content, and this data is therefore only available cross-sectionally. If attrition is independent of insecticide content, this should not cause a bias. However, if LLINs with low insecticide content are more likely to attrite than other LLINs, this will cause a bias overestimating insecticide content. Probably people do not throw their LLIN away because of low insecticide content (which is difficult to assess in the household), but insecticide content may be correlated to holed area (for a given LLIN age), and there is evidence that attrition is correlated with holed area.

**Limitations of the no-holes counterfactual**: For an estimate of the importance of decay of physical integrity, for comparison, a hypothetical scenario was modeled where all LLINs were intact, simulating ‘physically indestructible’ LLINs. On the one hand, this over-estimates the impact of preventing attrition, because the physical (and chemical) condition of the LLINs that would otherwise have been discarded is generally worse than that of the surviving LLINs. Because of this, the average LLIN condition of remaining LLINs may actually improve over time as the worst LLINs attrite. On the other hand, this does not allow for the secondary effects of physical damage on use and attrition (see main paper). The relative importance of maximizing use and LLIN survival was less for resistant mosquitoes than for susceptible mosquitoes, but these remained the hypothetical LLIN modifications with the highest impact. Perhaps counter intuitively, with increasing insecticide resistance, the proportion of residual VC that could be averted by maximising physical integrity reduced (except for in the Zambia study at 12 months).

## Tables of the potential effect of averting LLIN decay

**Table S10: Proportion of residual vectorial capacity prevented by improving LLIN durability properties in Angola,**

| Scenario | Semester | DawaPlus 2.0 | |
| --- | --- | --- | --- |
|  |  | Susceptible *An. gambiae* s.l. | Resistant *An. gambiae* s.l. |
| Intact | 2 | 0.13 (-1.56-0.68) | 0.00 (-0.94-0.48) |
|  | 4 | 0.06 (-0.69-0.49) | -0.02 (-0.39-0.24) |
|  | mean | 0.05 (-0.82-0.43) | -0.02 (-0.51-0.27) |
|  |  |  |  |
| Target content | 2 | 0.10 (-1.60-0.69) | 0.28 (-0.41-0.66) |
|  | 4 | -0.03 (-0.92-0.45) | 0.18 (-0.15-0.44) |
|  | mean | 0.00 (-0.91-0.42) | 0.22 (-0.17-0.45) |
|  |  |  |  |
| Maximum use | 2 | 0.84 (0.46-0.95) | 0.69 (0.33-0.88) |
|  | 4 | 0.81 (0.57-0.91) | 0.58 (0.36-0.73) |
|  | mean | 0.82 (0.62-0.91) | 0.63 (0.42-0.76) |
|  |  |  |  |
| Maximum survival | 2 | 0.71 (0.10-0.91) | 0.56 (0.06-0.78) |
|  | 4 | 0.77 (0.53-0.90) | 0.54 (0.33-0.69) |
|  | mean | 0.73 (0.42-0.87) | 0.54 (0.28-0.69) |

Figures between brackets are 95% credible intervals. Cells highlighted in green have credible intervals entirely above zero.

#### Table S11: Proportion of residual VC prevented by improving LLIN durability properties in Benin

| Scenario | Semester | Olyset | |
| --- | --- | --- | --- |
|  |  | Susceptible *An. gambiae* s.l. | Resistant *An. gambiae* s.l. |
| Intact | 1 | 0.08 (-1.19-0.63) | 0.05 (-0.93-0.56) |
|  | 2 | 0.65 (0.44-0.78) | 0.45 (0.23-0.60) |
|  | mean | 0.36 (-0.29-0.66) | 0.24 (-0.24-0.52) |
|  |  |  |  |
| Target content | 1 | 0.73 (0.24-0.90) | 0.35 (-0.35-0.70) |
|  | 2 | 0.87 (0.79-0.92) | 0.37 (0.18-0.52) |
|  | mean | 0.80 (0.55-0.90) | 0.36 (0.01-0.56) |
|  |  |  |  |
| Maximum use | 1 | 0.85 (0.72-0.92) | 0.82 (0.71-0.90) |
|  | 2 | 0.46 (0.31-0.60) | 0.39 (0.27-0.50) |
|  | mean | 0.65 (0.55-0.74) | 0.61 (0.52-0.67) |
|  |  |  |  |
| Maximum survival | 1 | 0.14 (-1.17-0.64) | 0.12 (-0.85-0.58) |
|  | 2 | 0.37 (0.09-0.56) | 0.30 (0.08-0.47) |
|  | mean | 0.24 (-0.39-0.53) | 0.20 (-0.29-0.46) |

Figures between brackets are 95% credible intervals. Cells highlighted in green have credible intervals entirely above zero. Cells highlighted in dark green has credible intervals that are entirely above zero in the analysis assuming LLIN efficacy against susceptible *An. gambiae* s.l., but had encompassed zero in the analysis assuming LLIN efficacy against resistant *An. gambiae* s.l.

#### Table S12: Proportion of residual vectorial capacity of susceptible *An. gambiae* s.l. prevented by improving LLIN durability properties in Kenya

| Scenario | Se-mester | DawaPlus 2.0 | DuraNet | Interceptor | Netprotect | Olyset | PermaNet 2.0 | PermaNet 3.0 | mean of all net brands |
| --- | --- | --- | --- | --- | --- | --- | --- | --- | --- |
| Intact | 2 | 0.05 (-0.98-0.56) | -0.02 (-1.06-0.52) | 0.15 (-0.91-0.61) | 0.05 (-0.65-0.46) | -0.02 (-0.83-0.44) | -0.01 (-0.83-0.45) | 0.00 (-0.91-0.46) | -0.02 (-0.34-0.22) |
|  | 4 | 0.17 (-0.75-0.58) | -0.01 (-1.08-0.53) | 0.61 (0.13-0.83) | 0.03 (-0.86-0.51) | 0.22 (-0.37-0.56) | 0.49 (0.10-0.72) | 0.24 (-0.57-0.64) | 0.22 (-0.05-0.40) |
|  | 6 | 0.19 (-0.41-0.57) | -0.01 (-0.98-0.52) | 0.34 (-0.34-0.68) | 0.12 (-0.57-0.53) | 0.19 (-0.37-0.55) | 0.42 (0.05-0.65) | 0.19 (-0.55-0.60) | 0.17 (-0.06-0.35) |
|  | 8 | 0.17 (-0.57-0.54) | 0.03 (-1.07-0.55) | 0.16 (-0.43-0.49) | 0.06 (-0.61-0.50) | 0.11 (-0.41-0.43) | 0.17 (-0.35-0.50) | 0.17 (-0.58-0.57) | 0.09 (-0.15-0.27) |
|  | mean | 0.11 (-0.25-0.37) | -0.05 (-0.52-0.28) | 0.28 (-0.08-0.50) | 0.03 (-0.31-0.30) | 0.11 (-0.19-0.31) | 0.25 (-0.02-0.43) | 0.12 (-0.26-0.37) | 0.11 (-0.01-0.22) |
|  |  |  |  |  |  |  |  |  |  |
| Target conc. | 2 | -0.08 (-1.33-0.47) | -0.72 (-2.46-0.16) | 0.09 (-1.15-0.58) | -0.01 (-0.77-0.41) | 0.03 (-0.78-0.46) | 0.01 (-0.84-0.46) | -2.76 (-5.45--1.10) | -0.56 (-1.10--0.18) |
|  | 4 | 0.07 (-0.84-0.54) | -1.01 (-3.09--0.01) | 0.68 (0.25-0.87) | -0.04 (-1.02-0.46) | 0.09 (-0.51-0.47) | 0.64 (0.34-0.81) | -4.30 (-9.25--1.55) | -0.59 (-1.34--0.12) |
|  | 6 | 0.26 (-0.33-0.59) | -0.85 (-2.76-0.05) | 0.43 (-0.17-0.72) | 0.17 (-0.55-0.56) | 0.04 (-0.60-0.41) | 0.66 (0.43-0.81) | -3.10 (-6.39--1.17) | -0.39 (-0.95-0.00) |
|  | 8 | 0.17 (-0.57-0.56) | -1.06 (-3.18--0.03) | 0.26 (-0.25-0.56) | 0.10 (-0.63-0.48) | 0.07 (-0.45-0.42) | 0.42 (-0.01-0.66) | -2.88 (-5.86--1.24) | -0.46 (-0.94--0.10) |
|  | mean | 0.07 (-0.36-0.35) | -0.98 (-1.87--0.44) | 0.32 (0.00-0.55) | 0.02 (-0.34-0.29) | 0.02 (-0.26-0.26) | 0.41 (0.17-0.58) | -3.37 (-5.02--2.16) | -0.52 (-0.80--0.30) |
|  |  |  |  |  |  |  |  |  |  |
| Max use | 2 | 1.00 (0.99-1.00) | 1.00 (1.00-1.00) | 0.99 (0.98-1.00) | 1.00 (1.00-1.00) | 1.00 (1.00-1.00) | 1.00 (1.00-1.00) | 1.00 (1.00-1.00) | 1.00 (1.00-1.00) |
|  | 4 | 0.93 (0.86-0.96) | 0.97 (0.93-0.99) | 0.70 (0.40-0.83) | 1.00 (0.99-1.00) | 0.99 (0.98-0.99) | 0.87 (0.80-0.92) | 0.93 (0.85-0.97) | 0.91 (0.87-0.93) |
|  | 6 | 0.93 (0.89-0.96) | 0.99 (0.97-1.00) | 0.89 (0.79-0.94) | 0.99 (0.99-1.00) | 0.98 (0.97-0.99) | 0.63 (0.42-0.75) | 0.90 (0.80-0.95) | 0.90 (0.87-0.92) |
|  | 8 | 0.69 (0.46-0.83) | 0.96 (0.92-0.98) | 0.28 (-0.25-0.56) | 0.92 (0.86-0.96) | 0.97 (0.96-0.98) | 0.61 (0.36-0.76) | 0.78 (0.57-0.88) | 0.74 (0.65-0.80) |
|  | mean | 0.88 (0.82-0.92) | 0.98 (0.96-0.99) | 0.71 (0.56-0.80) | 0.98 (0.96-0.99) | 0.98 (0.98-0.99) | 0.77 (0.70-0.83) | 0.90 (0.84-0.93) | 0.89 (0.86-0.90) |
|  |  |  |  |  |  |  |  |  |  |
| Max surv-ival | 2 | 0.05 (-0.99-0.55) | 0.05 (-0.92-0.51) | 0.11 (-1.05-0.60) | 0.01 (-0.74-0.41) | 0.04 (-0.71-0.47) | 0.07 (-0.69-0.48) | -0.01 (-0.86-0.45) | -0.01 (-0.30-0.21) |
|  | 4 | 0.42 (-0.24-0.71) | 0.35 (-0.40-0.68) | 0.23 (-0.46-0.60) | 0.08 (-0.78-0.53) | 0.07 (-0.59-0.46) | 0.17 (-0.38-0.50) | 0.14 (-0.77-0.57) | 0.17 (-0.09-0.34) |
|  | 6 | 0.38 (-0.10-0.66) | 0.27 (-0.47-0.63) | 0.44 (-0.15-0.70) | 0.07 (-0.66-0.51) | 0.18 (-0.41-0.52) | 0.63 (0.39-0.77) | 0.34 (-0.33-0.65) | 0.30 (0.10-0.45) |
|  | 8 | 0.81 (0.62-0.91) | 0.49 (-0.16-0.77) | 0.97 (0.94-0.98) | 0.59 (0.16-0.79) | 0.20 (-0.24-0.50) | 0.79 (0.61-0.89) | 0.58 (0.14-0.78) | 0.62 (0.49-0.71) |
|  | mean | 0.39 (0.08-0.59) | 0.25 (-0.09-0.48) | 0.41 (0.08-0.61) | 0.16 (-0.17-0.39) | 0.10 (-0.18-0.31) | 0.39 (0.15-0.55) | 0.23 (-0.09-0.46) | 0.26 (0.16-0.36) |

Figures between brackets are 95% credible intervals. Cells highlighted in green have credible intervals entirely above zero. Cells highlighted in purple have credible intervals that encompass zero, but were entirely above zero in the analysis assuming LLIN efficacy against resistant *An. gambiae* s.l. Cells highlighted in yellow have credible intervals entirely below zero, and cells highlighted in orange have credible intervals entirely below zero, but encompassed zero in the analysis assuming LLIN efficacy against resistant *An. gambiae* s.l.

#### Table S13: Proportion of residual vectorial capacity of resistant *An. gambiae* s.l. prevented by improving LLIN durability properties in Kenya

| Scen-ario | Se- mester | DawaPlus 2.0 | DuraNet | Interceptor | Netprotect | Olyset | PermaNet 2.0 | PermaNet 3.0 | Mean of all LLIN brands |
| --- | --- | --- | --- | --- | --- | --- | --- | --- | --- |
| Intact | 2 | -0.01 (-0.74-0.42) | 0.01 (-0.87-0.48) | 0.18 (-0.47-0.53) | 0.01 (-0.33-0.29) | 0.01 (-0.34-0.24) | -0.03 (-0.43-0.28) | 0.02 (-0.76-0.42) | 0.00 (-0.20-0.16) |
|  | 4 | 0.09 (-0.48-0.45) | -0.03 (-0.93-0.45) | 0.66 (0.36-0.81) | -0.02 (-0.61-0.36) | 0.12 (-0.10-0.30) | 0.33 (0.01-0.55) | 0.15 (-0.70-0.59) | 0.16 (-0.02-0.32) |
|  | 6 | 0.05 (-0.31-0.33) | -0.03 (-0.72-0.39) | 0.21 (-0.18-0.50) | 0.04 (-0.34-0.33) | 0.10 (-0.10-0.29) | 0.24 (-0.05-0.44) | 0.14 (-0.48-0.51) | 0.09 (-0.06-0.22) |
|  | 8 | 0.03 (-0.33-0.31) | -0.05 (-0.72-0.37) | 0.06 (-0.18-0.26) | 0.00 (-0.30-0.24) | 0.05 (-0.09-0.18) | 0.05 (-0.20-0.27) | 0.12 (-0.47-0.47) | 0.03 (-0.12-0.13) |
|  | mean | 0.03 (-0.21-0.21) | -0.05 (-0.42-0.20) | 0.27 (0.07-0.43) | 0.00 (-0.20-0.16) | 0.07 (-0.05-0.17) | 0.14 (-0.02-0.27) | 0.07 (-0.26-0.31) | 0.07 (-0.02-0.15) |
|  |  |  |  |  |  |  |  |  |  |
| Target conc. | 2 | 0.26 (-0.25-0.57) | -0.23 (-1.25-0.34) | 0.33 (-0.20-0.63) | 0.17 (-0.15-0.38) | 0.01 (-0.32-0.24) | 0.27 (-0.06-0.49) | -0.55 (-1.65-0.07) | 0.01 (-0.20-0.18) |
|  | 4 | 0.47 (0.10-0.68) | -0.29 (-1.48-0.26) | 0.76 (0.58-0.86) | 0.21 (-0.27-0.50) | 0.03 (-0.19-0.22) | 0.64 (0.46-0.76) | -0.44 (-1.73-0.30) | 0.17 (-0.06-0.35) |
|  | 6 | 0.39 (0.12-0.58) | -0.31 (-1.17-0.21) | 0.46 (0.17-0.65) | 0.11 (-0.24-0.37) | 0.01 (-0.18-0.17) | 0.60 (0.44-0.72) | -0.38 (-1.28-0.20) | 0.11 (-0.07-0.27) |
|  | 8 | 0.37 (0.10-0.55) | -0.29 (-1.11-0.22) | 0.30 (0.10-0.47) | 0.07 (-0.22-0.31) | 0.02 (-0.12-0.14) | 0.37 (0.12-0.53) | -0.32 (-1.12-0.18) | 0.07 (-0.10-0.20) |
|  | mean | 0.36 (0.18-0.49) | -0.32 (-0.73--0.02) | 0.45 (0.29-0.56) | 0.13 (-0.04-0.27) | 0.02 (-0.10-0.12) | 0.47 (0.35-0.56) | -0.46 (-0.92--0.10) | 0.08 (-0.01-0.17) |
|  |  |  |  |  |  |  |  |  |  |
| Max use | 2 | 0.99 (0.98-0.99) | 1.00 (1.00-1.00) | 0.92 (0.87-0.95) | 0.98 (0.97-0.98) | 0.98 (0.97-0.98) | 0.99 (0.98-1.00) | 1.00 (1.00-1.00) | 0.98 (0.97-0.98) |
|  | 4 | 0.85 (0.76-0.91) | 0.97 (0.91-0.99) | 0.49 (0.25-0.64) | 0.96 (0.94-0.97) | 0.86 (0.83-0.88) | 0.77 (0.69-0.83) | 0.94 (0.87-0.97) | 0.83 (0.79-0.86) |
|  | 6 | 0.83 (0.75-0.88) | 0.98 (0.95-0.99) | 0.74 (0.62-0.82) | 0.95 (0.93-0.97) | 0.81 (0.76-0.84) | 0.48 (0.32-0.61) | 0.88 (0.78-0.94) | 0.81 (0.77-0.83) |
|  | 8 | 0.48 (0.28-0.63) | 0.92 (0.85-0.96) | 0.14 (-0.08-0.32) | 0.74 (0.65-0.82) | 0.76 (0.71-0.80) | 0.40 (0.22-0.53) | 0.73 (0.55-0.84) | 0.59 (0.54-0.64) |
|  | mean | 0.78 (0.73-0.83) | 0.96 (0.94-0.98) | 0.57 (0.49-0.63) | 0.91 (0.88-0.93) | 0.85 (0.83-0.87) | 0.66 (0.60-0.71) | 0.88 (0.83-0.92) | 0.80 (0.78-0.82) |
|  |  |  |  |  |  |  |  |  |  |
| Max surv-ival | 2 | 0.07 (-0.57-0.43) | 0.04 (-0.72-0.49) | 0.05 (-0.61-0.44) | -0.01 (-0.34-0.26) | 0.01 (-0.33-0.25) | 0.04 (-0.39-0.32) | 0.01 (-0.75-0.41) | 0.01 (-0.18-0.16) |
|  | 4 | 0.30 (-0.16-0.58) | 0.32 (-0.32-0.65) | 0.16 (-0.22-0.43) | 0.05 (-0.45-0.39) | 0.03 (-0.19-0.20) | 0.11 (-0.23-0.35) | 0.10 (-0.71-0.52) | 0.13 (-0.04-0.27) |
|  | 6 | 0.24 (-0.07-0.45) | 0.22 (-0.39-0.56) | 0.27 (-0.11-0.53) | 0.06 (-0.30-0.30) | 0.07 (-0.12-0.23) | 0.48 (0.29-0.61) | 0.28 (-0.26-0.59) | 0.22 (0.09-0.33) |
|  | 8 | 0.63 (0.40-0.78) | 0.38 (-0.08-0.64) | 0.85 (0.77-0.90) | 0.36 (0.07-0.55) | 0.07 (-0.07-0.21) | 0.60 (0.42-0.73) | 0.51 (0.13-0.73) | 0.48 (0.37-0.55) |
|  | mean | 0.29 (0.10-0.45) | 0.21 (-0.10-0.43) | 0.32 (0.13-0.47) | 0.10 (-0.07-0.26) | 0.04 (-0.08-0.14) | 0.30 (0.16-0.41) | 0.21 (-0.11-0.40) | 0.21 (0.13-0.27) |

Figures between brackets are 95% credible intervals. Cells highlighted in green have credible intervals entirely above zero; cells highlighted in yellow have credible intervals entirely below zero.

#### Table S14: Proportion of residual vectorial capacity of susceptible *An. gambiae* s.l. prevented by improving LLIN durability properties in Malawi

| Scenario | Semester | DuraNet | Interceptor | Netprotect | Olyset | PermaNet 2.0 | mean of all net brands |
| --- | --- | --- | --- | --- | --- | --- | --- |
| Intact | 2 | -0.01 (-0.94-0.47) | 0.20 (-0.91-0.66) | 0.03 (-1.49-0.62) | 0.45 (-0.17-0.73) | 0.11 (-1.04-0.61) | 0.10 (-0.33-0.38) |
|  | 4 | 0.02 (-1.19-0.60) | 0.62 (0.28-0.80) | 0.57 (0.03-0.81) | 0.70 (0.45-0.84) | 0.30 (-0.34-0.67) | 0.42 (0.13-0.60) |
|  | 6 | 0.01 (-0.73-0.43) | 0.25 (-0.22-0.54) | 0.69 (0.43-0.82) | 0.50 (0.21-0.70) | 0.47 (0.21-0.65) | 0.37 (0.20-0.51) |
|  | Mean | -0.03 (-0.54-0.31) | 0.34 (-0.06-0.56) | 0.41 (-0.10-0.65) | 0.53 (0.32-0.67) | 0.27 (-0.14-0.52) | 0.29 (0.11-0.42) |
|  |  |  |  |  |  |  |  |
| Target content | 2 | -0.20 (-1.24-0.36) | 0.17 (-0.90-0.65) | 0.00 (-1.81-0.62) | 0.04 (-0.80-0.49) | 0.03 (-1.28-0.59) | -0.05 (-0.52-0.27) |
|  | 4 | -0.57 (-2.60-0.31) | 0.79 (0.58-0.90) | 0.50 (-0.04-0.77) | 0.14 (-0.38-0.48) | 0.55 (0.10-0.79) | 0.25 (-0.17-0.48) |
|  | 6 | -0.36 (-1.31-0.17) | 0.50 (0.17-0.70) | 0.80 (0.63-0.89) | 0.13 (-0.27-0.42) | 0.63 (0.42-0.77) | 0.33 (0.10-0.48) |
|  | Mean | -0.44 (-1.22-0.02) | 0.47 (0.07-0.67) | 0.42 (-0.19-0.66) | 0.09 (-0.23-0.33) | 0.38 (-0.04-0.62) | 0.16 (-0.04-0.32) |
|  |  |  |  |  |  |  |  |
| Maximum use | 2 | 1.00 (0.99-1.00) | 0.99 (0.97-0.99) | 1.00 (1.00-1.00) | 0.95 (0.92-0.97) | 1.00 (0.99-1.00) | 0.99 (0.98-0.99) |
|  | 4 | 0.95 (0.85-0.98) | 0.49 (0.21-0.69) | 0.57 (0.21-0.77) | 0.80 (0.71-0.87) | 0.90 (0.82-0.95) | 0.73 (0.64-0.80) |
|  | 6 | 0.57 (0.24-0.76) | 0.36 (-0.01-0.58) | 0.34 (0.01-0.57) | 0.65 (0.48-0.75) | 0.38 (0.14-0.56) | 0.45 (0.32-0.55) |
|  | Mean | 0.84 (0.72-0.90) | 0.61 (0.45-0.71) | 0.63 (0.47-0.75) | 0.80 (0.74-0.84) | 0.76 (0.68-0.82) | 0.72 (0.67-0.77) |
|  |  |  |  |  |  |  |  |
| Maximum survival | 2 | 0.14 (-0.74-0.56) | 0.02 (-1.09-0.56) | 0.02 (-1.38-0.63) | 0.04 (-0.74-0.48) | 0.03 (-1.31-0.57) | -0.01 (-0.43-0.29) |
|  | 4 | 0.47 (-0.38-0.80) | 0.57 (0.25-0.74) | 0.59 (0.17-0.80) | 0.24 (-0.26-0.52) | 0.28 (-0.31-0.61) | 0.40 (0.19-0.55) |
|  | 6 | 0.92 (0.83-0.97) | 0.91 (0.83-0.94) | 0.67 (0.50-0.79) | 0.70 (0.52-0.81) | 0.72 (0.58-0.81) | 0.78 (0.72-0.82) |
|  | Mean | 0.49 (0.12-0.69) | 0.49 (0.11-0.70) | 0.41 (-0.05-0.64) | 0.31 (0.01-0.51) | 0.33 (-0.14-0.57) | 0.39 (0.24-0.51) |

Figures between brackets are 95% credible intervals. Cells highlighted in green have credible intervals entirely above zero. Cells highlighted in purple have credible intervals that encompass zero, but were entirely above zero in the analysis assuming LLIN efficacy against resistant *An. gambiae* s.l.

#### Table S15: Proportion of residual vectorial capacity of resistant *An. gambiae* s.l. prevented by improving LLIN durability properties in Malawi

| Scenario | Semester | DuraNet | Interceptor | Netprotect | Olyset | PermaNet 2.0 | Mean of all LLIN brands |
| --- | --- | --- | --- | --- | --- | --- | --- |
| Intact | 2 | 0.00 (-0.68-0.40) | 0.18 (-0.45-0.53) | 0.00 (-0.85-0.51) | 0.40 (0.07-0.61) | 0.07 (-0.64-0.50) | 0.10 (-0.17-0.32) |
|  | 4 | 0.01 (-0.94-0.50) | 0.48 (0.21-0.66) | 0.66 (0.42-0.79) | 0.52 (0.35-0.66) | 0.18 (-0.26-0.49) | 0.36 (0.15-0.50) |
|  | 6 | 0.01 (-0.45-0.33) | 0.10 (-0.13-0.30) | 0.46 (0.27-0.63) | 0.27 (0.10-0.41) | 0.22 (0.05-0.37) | 0.21 (0.10-0.31) |
|  | Mean | -0.01 (-0.39-0.25) | 0.24 (0.02-0.40) | 0.37 (0.07-0.56) | 0.39 (0.27-0.50) | 0.14 (-0.13-0.34) | 0.22 (0.10-0.31) |
|  |  |  |  |  |  |  |  |
| Target content | 2 | -0.08 (-0.88-0.38) | 0.44 (0.02-0.69) | 0.22 (-0.50-0.60) | 0.01 (-0.37-0.28) | 0.31 (-0.22-0.62) | 0.16 (-0.07-0.34) |
|  | 4 | -0.17 (-1.28-0.44) | 0.73 (0.59-0.82) | 0.68 (0.52-0.79) | 0.05 (-0.15-0.22) | 0.59 (0.36-0.76) | 0.37 (0.13-0.51) |
|  | 6 | -0.10 (-0.58-0.22) | 0.47 (0.30-0.60) | 0.71 (0.60-0.79) | 0.04 (-0.11-0.19) | 0.51 (0.37-0.62) | 0.32 (0.21-0.41) |
|  | Mean | -0.13 (-0.56-0.17) | 0.54 (0.39-0.65) | 0.53 (0.30-0.68) | 0.03 (-0.12-0.16) | 0.46 (0.26-0.60) | 0.28 (0.18-0.37) |
|  |  |  |  |  |  |  |  |
| Maximum use | 2 | 1.00 (0.98-1.00) | 0.92 (0.87-0.95) | 0.98 (0.97-0.99) | 0.76 (0.70-0.81) | 0.98 (0.97-0.99) | 0.93 (0.91-0.94) |
|  | 4 | 0.92 (0.80-0.97) | 0.36 (0.18-0.52) | 0.33 (0.06-0.51) | 0.53 (0.43-0.61) | 0.83 (0.74-0.88) | 0.59 (0.52-0.65) |
|  | 6 | 0.46 (0.20-0.64) | 0.19 (0.00-0.36) | 0.21 (0.02-0.37) | 0.34 (0.23-0.44) | 0.20 (0.07-0.32) | 0.28 (0.20-0.35) |
|  | Mean | 0.79 (0.70-0.86) | 0.49 (0.40-0.57) | 0.50 (0.39-0.59) | 0.54 (0.49-0.59) | 0.67 (0.62-0.71) | 0.60 (0.57-0.63) |
|  |  |  |  |  |  |  |  |
| Maximum survival | 2 | 0.11 (-0.52-0.48) | 0.05 (-0.57-0.41) | 0.02 (-0.94-0.49) | 0.02 (-0.34-0.28) | 0.02 (-0.70-0.45) | 0.02 (-0.25-0.22) |
|  | 4 | 0.43 (-0.24-0.74) | 0.42 (0.20-0.59) | 0.34 (0.08-0.53) | 0.11 (-0.10-0.28) | 0.20 (-0.18-0.49) | 0.29 (0.13-0.41) |
|  | 6 | 0.86 (0.74-0.93) | 0.74 (0.64-0.82) | 0.47 (0.33-0.57) | 0.39 (0.27-0.49) | 0.47 (0.35-0.57) | 0.58 (0.53-0.63) |
|  | Mean | 0.44 (0.16-0.65) | 0.40 (0.18-0.54) | 0.27 (-0.06-0.44) | 0.17 (0.03-0.28) | 0.22 (-0.04-0.41) | 0.30 (0.19-0.38) |

Figures between brackets are 95% credible intervals. Cells highlighted in green have credible intervals entirely above zero.

#### Table S16: Proportion of residual vectorial capacity of susceptible *An. gambiae* s.l. prevented by improving LLIN durability properties in Mozambique

| Scenario | Semester | Olyset | PermaNet 2.0 Coastal | PermaNet 2.0 Inland | Mean of all net brands and settings |
| --- | --- | --- | --- | --- | --- |
| Intact | 2 | 0.24 (-0.86-0.67) | 0.00 (-2.45-0.71) | 0.01 (-1.76-0.63) | 0.02 (-0.90-0.48) |
|  | 4 | 0.16 (-0.61-0.58) | 0.10 (-1.49-0.68) | 0.01 (-1.38-0.62) | 0.05 (-0.61-0.42) |
|  | 6 | 0.30 (-0.27-0.65) | 0.22 (-0.41-0.60) | 0.15 (-0.56-0.55) | 0.20 (-0.17-0.45) |
|  | mean | 0.20 (-0.23-0.48) | 0.04 (-0.90-0.46) | 0.01 (-0.65-0.40) | 0.06 (-0.33-0.31) |
|  |  |  |  |  |  |
| Target content | 2 | 0.11 (-0.96-0.61) | -0.03 (-2.71-0.75) | 0.06 (-1.55-0.66) | -0.05 (-1.01-0.42) |
|  | 4 | 0.11 (-0.59-0.52) | 0.10 (-1.57-0.66) | 0.02 (-1.22-0.59) | 0.02 (-0.69-0.41) |
|  | 6 | 0.10 (-0.56-0.50) | 0.46 (-0.03-0.74) | 0.40 (-0.24-0.74) | 0.30 (-0.04-0.53) |
|  | mean | 0.07 (-0.40-0.37) | 0.11 (-0.85-0.55) | 0.11 (-0.53-0.49) | 0.07 (-0.34-0.33) |
|  |  |  |  |  |  |
| Maximum use | 2 | 0.98 (0.97-0.99) | 1.00 (1.00-1.00) | 1.00 (1.00-1.00) | 0.99 (0.99-1.00) |
|  | 4 | 0.98 (0.97-0.99) | 1.00 (0.99-1.00) | 1.00 (0.99-1.00) | 0.99 (0.99-0.99) |
|  | 6 | 0.92 (0.86-0.95) | 0.87 (0.76-0.93) | 0.87 (0.73-0.93) | 0.88 (0.83-0.92) |
|  | mean | 0.96 (0.94-0.97) | 0.96 (0.92-0.97) | 0.95 (0.91-0.97) | 0.96 (0.94-0.97) |
|  |  |  |  |  |  |
| Maximum survival | 2 | 0.02 (-1.32-0.54) | -0.03 (-2.42-0.68) | -0.03 (-1.94-0.62) | -0.09 (-1.16-0.36) |
|  | 4 | 0.05 (-0.70-0.45) | 0.04 (-1.57-0.64) | 0.12 (-1.28-0.64) | 0.01 (-0.65-0.38) |
|  | 6 | 0.13 (-0.54-0.51) | 0.22 (-0.37-0.58) | 0.29 (-0.47-0.65) | 0.19 (-0.16-0.44) |
|  | mean | 0.04 (-0.52-0.34) | 0.03 (-0.95-0.43) | 0.07 (-0.64-0.45) | 0.03 (-0.41-0.25) |

Figures between brackets are 95% credible intervals. Cells highlighted in green have credible intervals entirely above zero. Cells highlighted in purple have credible intervals that encompass zero, but were entirely above zero in the analysis assuming LLIN efficacy against resistant *An. gambiae* s.l.

#### Table S17: Proportion of residual vectorial capacity of resistant *An. gambiae* s.l. prevented by improving LLIN durability properties in Mozambique

| Scenario | Semester | Olyset | PermaNet 2.0 Coastal | PermaNet 2.0 Inland | Mean of all LLIN brands and settings |
| --- | --- | --- | --- | --- | --- |
| Intact | 2 | 0.12 (-0.21-0.38) | -0.06 (-1.08-0.50) | -0.03 (-0.65-0.37) | 0.00 (-0.37-0.27) |
|  | 4 | 0.05 (-0.14-0.23) | 0.03 (-0.52-0.39) | -0.03 (-0.50-0.29) | 0.01 (-0.24-0.20) |
|  | 6 | 0.10 (-0.10-0.30) | 0.09 (-0.16-0.30) | 0.04 (-0.20-0.27) | 0.08 (-0.05-0.21) |
|  | mean | 0.09 (-0.05-0.22) | 0.01 (-0.37-0.26) | -0.01 (-0.27-0.18) | 0.02 (-0.13-0.15) |
|  |  |  |  |  |  |
| Target content | 2 | 0.03 (-0.29-0.27) | 0.18 (-0.63-0.63) | 0.19 (-0.40-0.54) | 0.12 (-0.22-0.36) |
|  | 4 | 0.03 (-0.16-0.19) | 0.32 (-0.10-0.63) | 0.15 (-0.31-0.47) | 0.16 (-0.05-0.32) |
|  | 6 | 0.03 (-0.14-0.18) | 0.34 (0.11-0.57) | 0.32 (0.04-0.56) | 0.23 (0.10-0.36) |
|  | mean | 0.03 (-0.09-0.14) | 0.27 (-0.05-0.48) | 0.21 (-0.03-0.39) | 0.17 (0.04-0.27) |
|  |  |  |  |  |  |
| Maximum use | 2 | 0.82 (0.77-0.85) | 0.99 (0.98-0.99) | 0.99 (0.99-1.00) | 0.94 (0.92-0.95) |
|  | 4 | 0.81 (0.75-0.84) | 0.95 (0.91-0.96) | 0.96 (0.91-0.98) | 0.90 (0.88-0.92) |
|  | 6 | 0.63 (0.54-0.70) | 0.67 (0.54-0.76) | 0.66 (0.50-0.78) | 0.65 (0.58-0.71) |
|  | mean | 0.75 (0.72-0.78) | 0.87 (0.82-0.90) | 0.87 (0.82-0.91) | 0.83 (0.80-0.85) |
|  |  |  |  |  |  |
| Maximum survival | 2 | 0.00 (-0.29-0.25) | -0.01 (-0.95-0.50) | 0.01 (-0.66-0.38) | -0.02 (-0.39-0.24) |
|  | 4 | 0.01 (-0.15-0.18) | 0.02 (-0.52-0.37) | 0.04 (-0.37-0.37) | 0.02 (-0.20-0.19) |
|  | 6 | 0.04 (-0.14-0.19) | 0.11 (-0.13-0.33) | 0.13 (-0.11-0.36) | 0.09 (-0.04-0.21) |
|  | mean | 0.02 (-0.11-0.13) | 0.03 (-0.33-0.26) | 0.06 (-0.19-0.25) | 0.03 (-0.11-0.14) |

Figures between brackets are 95% credible intervals. Cells highlighted in green have credible intervals entirely above zero.

#### Table S18: Proportion of residual VC of susceptible *An. gambiae* s.l. prevented by improving LLINs in Senegal

| Scenario | Semester | DawaPlus 2.0 | Netprotect | Olyset | PermaNet 2.0 | LifeNet | mean of all net brands |
| --- | --- | --- | --- | --- | --- | --- | --- |
| Intact | 1 |  | 0.00 (-1.05-0.48) | 0.78 (0.64-0.87) |  | 0.01 (-0.39-0.27) | 0.25 (-0.10-0.45) |
|  | 2 |  | 0.06 (-0.86-0.51) | 0.31 (-0.01-0.53) |  | -0.01 (-1.10-0.51) | 0.09 (-0.38-0.38) |
|  | 3 | 0.07 (-0.87-0.55) | 0.04 (-0.94-0.54) | 0.84 (0.75-0.91) | 0.01 (-0.93-0.52) | -0.01 (-0.84-0.51) | 0.16 (-0.20-0.41) |
|  | 4 | 0.24 (-0.73-0.66) | 0.02 (-1.09-0.50) | 0.67 (0.51-0.78) | 0.11 (-0.97-0.59) | 0.00 (-1.24-0.53) | 0.17 (-0.24-0.40) |
|  | 5 | 0.04 (-0.75-0.50) | 0.00 (-0.35-0.26) | 0.83 (0.71-0.89) | 0.01 (-0.81-0.48) | 0.01 (-1.27-0.60) | 0.16 (-0.16-0.37) |
|  | 6 | 0.01 (-0.43-0.29) | 0.00 (-0.15-0.13) | 0.76 (0.58-0.86) | 0.02 (-0.33-0.30) | -0.04 (-7.75-0.88) | 0.13 (-1.39-0.37) |
|  | mean | 0.05 (-0.34-0.31) | -0.02 (-0.34-0.19) | 0.69 (0.62-0.75) | 0.01 (-0.38-0.26) | -0.09 (-1.37-0.26) | 0.13 (-0.17-0.26) |
|  |  |  |  |  |  |  |  |
| Target content | 1 |  | -0.02 (-1.22-0.47) | 0.29 (-0.07-0.51) |  | 0.23 (-0.10-0.47) | 0.16 (-0.23-0.37) |
|  | 2 |  | 0.02 (-0.93-0.50) | 0.15 (-0.27-0.41) |  | 0.33 (-0.42-0.67) | 0.14 (-0.25-0.39) |
|  | 3 | -0.08 (-1.29-0.46) | 0.04 (-1.03-0.51) | 0.42 (0.17-0.59) | 0.00 (-1.08-0.51) | 0.31 (-0.32-0.67) | 0.10 (-0.27-0.34) |
|  | 4 | 0.08 (-1.15-0.58) | 0.01 (-1.04-0.52) | 0.28 (0.02-0.46) | 0.02 (-1.11-0.55) | 0.22 (-0.70-0.68) | 0.08 (-0.33-0.35) |
|  | 5 | 0.04 (-0.78-0.50) | 0.00 (-0.35-0.26) | 0.43 (0.18-0.59) | 0.00 (-0.76-0.47) | 0.07 (-1.26-0.66) | 0.08 (-0.25-0.29) |
|  | 6 | 0.00 (-0.46-0.28) | 0.00 (-0.15-0.13) | 0.40 (0.11-0.58) | 0.00 (-0.34-0.28) | 0.28 (-6.85-0.93) | 0.12 (-1.29-0.30) |
|  | mean | -0.02 (-0.48-0.26) | -0.03 (-0.34-0.18) | 0.32 (0.20-0.41) | -0.02 (-0.45-0.24) | 0.18 (-0.98-0.45) | 0.09 (-0.19-0.21) |
|  |  |  |  |  |  |  |  |
| Maximum use | 1 |  | 1.00 (1.00-1.00) | 0.78 (0.70-0.83) |  | 1.00 (1.00-1.00) | 0.93 (0.90-0.94) |
|  | 2 |  | 1.00 (1.00-1.00) | 0.98 (0.97-0.99) |  | 1.00 (1.00-1.00) | 0.99 (0.99-1.00) |
|  | 3 | 1.00 (0.99-1.00) | 1.00 (1.00-1.00) | 0.65 (0.55-0.74) | 1.00 (1.00-1.00) | 1.00 (1.00-1.00) | 0.93 (0.91-0.95) |
|  | 4 | 0.99 (0.97-0.99) | 1.00 (1.00-1.00) | 0.83 (0.78-0.87) | 1.00 (0.99-1.00) | 1.00 (1.00-1.00) | 0.96 (0.95-0.97) |
|  | 5 | 1.00 (1.00-1.00) | 1.00 (1.00-1.00) | 0.31 (0.06-0.49) | 1.00 (1.00-1.00) | 1.00 (1.00-1.00) | 0.86 (0.81-0.90) |
|  | 6 | 1.00 (1.00-1.00) | 1.00 (1.00-1.00) | 0.50 (0.33-0.63) | 1.00 (1.00-1.00) | 1.00 (1.00-1.00) | 0.90 (0.87-0.93) |
|  | mean | 1.00 (0.99-1.00) | 1.00 (1.00-1.00) | 0.67 (0.62-0.72) | 1.00 (1.00-1.00) | 1.00 (1.00-1.00) | 0.92 (0.91-0.93) |
|  |  |  |  |  |  |  |  |
| Maximum survival | 1 |  | 0.00 (-1.03-0.50) | 0.01 (-0.44-0.32) |  | 0.00 (-0.37-0.29) | -0.01 (-0.39-0.24) |
|  | 2 |  | 0.03 (-0.86-0.50) | 0.04 (-0.40-0.33) |  | 0.03 (-1.07-0.53) | 0.00 (-0.45-0.30) |
|  | 3 | 0.08 (-0.89-0.58) | 0.04 (-1.04-0.51) | 0.11 (-0.26-0.37) | 0.07 (-0.89-0.54) | 0.05 (-0.80-0.48) | 0.04 (-0.35-0.28) |
|  | 4 | 0.10 (-0.98-0.60) | 0.03 (-0.97-0.52) | 0.17 (-0.09-0.39) | 0.04 (-1.02-0.57) | 0.02 (-1.12-0.56) | 0.03 (-0.36-0.27) |
|  | 5 | 0.01 (-0.86-0.48) | 0.01 (-0.33-0.28) | 0.60 (0.44-0.71) | 0.03 (-0.80-0.46) | 0.02 (-1.28-0.59) | 0.11 (-0.21-0.32) |
|  | 6 | 0.01 (-0.43-0.31) | 0.00 (-0.15-0.14) | 0.51 (0.30-0.65) | 0.00 (-0.34-0.28) | 0.05 (-8.14-0.91) | 0.10 (-1.55-0.32) |
|  | mean | 0.02 (-0.41-0.28) | -0.01 (-0.32-0.19) | 0.23 (0.11-0.33) | 0.00 (-0.36-0.27) | -0.06 (-1.44-0.28) | 0.03 (-0.27-0.16) |

Figures between brackets are 95% credible intervals. Cells highlighted in green have credible intervals entirely above zero. Cells highlighted in purple have credible intervals that encompass zero, but were entirely above zero in the analysis assuming LLIN efficacy against resistant *An. gambiae* s.l.

#### Table S19: Proportion of residual VC of resistant *An. gambiae* s.l. prevented by improving LLINs in Senegal

| Scenario | Semester | DawaPlus 2.0 | Netprotect | Olyset | PermaNet 2.0 | LifeNet | Mean of all LLIN brands |
| --- | --- | --- | --- | --- | --- | --- | --- |
| Intact | 1 |  | -0.03 (-0.45-0.26) | 0.67 (0.54-0.75) |  | 0.01 (-0.20-0.17) | 0.21 (0.06-0.33) |
|  | 2 |  | -0.02 (-0.35-0.24) | 0.19 (0.03-0.33) |  | -0.01 (-0.64-0.38) | 0.05 (-0.19-0.21) |
|  | 3 | -0.01 (-0.60-0.37) | -0.01 (-0.48-0.29) | 0.71 (0.60-0.79) | -0.07 (-0.71-0.28) | -0.02 (-0.61-0.35) | 0.10 (-0.11-0.26) |
|  | 4 | 0.13 (-0.44-0.49) | 0.01 (-0.28-0.22) | 0.39 (0.28-0.49) | 0.02 (-0.50-0.38) | 0.00 (-0.58-0.34) | 0.09 (-0.10-0.24) |
|  | 5 | -0.01 (-0.32-0.23) | -0.01 (-0.11-0.10) | 0.61 (0.48-0.71) | -0.04 (-0.37-0.22) | 0.00 (-0.61-0.38) | 0.11 (-0.04-0.21) |
|  | 6 | -0.01 (-0.19-0.14) | 0.00 (-0.05-0.05) | 0.43 (0.29-0.57) | -0.01 (-0.15-0.12) | -0.02 (-3.22-0.76) | 0.07 (-0.56-0.24) |
|  | mean | 0.02 (-0.20-0.18) | -0.02 (-0.14-0.08) | 0.50 (0.45-0.54) | -0.03 (-0.25-0.12) | -0.04 (-0.59-0.18) | 0.10 (-0.04-0.17) |
|  |  |  |  |  |  |  |  |
| Target content | 1 |  | 0.09 (-0.32-0.38) | 0.11 (-0.05-0.26) |  | 0.06 (-0.14-0.23) | 0.08 (-0.07-0.21) |
|  | 2 |  | 0.12 (-0.20-0.36) | 0.06 (-0.08-0.21) |  | 0.11 (-0.46-0.45) | 0.09 (-0.12-0.25) |
|  | 3 | 0.24 (-0.18-0.52) | 0.10 (-0.29-0.39) | 0.17 (0.01-0.29) | 0.07 (-0.41-0.39) | 0.07 (-0.49-0.42) | 0.12 (-0.05-0.26) |
|  | 4 | 0.32 (-0.16-0.61) | 0.17 (-0.16-0.40) | 0.07 (-0.03-0.18) | 0.19 (-0.29-0.49) | 0.05 (-0.52-0.41) | 0.15 (-0.02-0.28) |
|  | 5 | 0.09 (-0.23-0.34) | 0.01 (-0.10-0.12) | 0.16 (0.03-0.28) | 0.04 (-0.26-0.29) | 0.02 (-0.58-0.42) | 0.06 (-0.10-0.17) |
|  | 6 | 0.01 (-0.16-0.18) | 0.00 (-0.05-0.06) | 0.11 (-0.03-0.23) | 0.02 (-0.11-0.16) | 0.05 (-2.39-0.78) | 0.04 (-0.46-0.20) |
|  | mean | 0.16 (-0.02-0.29) | 0.08 (-0.04-0.17) | 0.11 (0.06-0.17) | 0.07 (-0.10-0.22) | 0.03 (-0.38-0.26) | 0.08 (-0.02-0.15) |
|  |  |  |  |  |  |  |  |
| Maximum use | 1 |  | 1.00 (1.00-1.00) | 0.53 (0.46-0.59) |  | 1.00 (1.00-1.00) | 0.84 (0.82-0.86) |
|  | 2 |  | 0.99 (0.99-0.99) | 0.86 (0.83-0.88) |  | 1.00 (1.00-1.00) | 0.95 (0.94-0.96) |
|  | 3 | 0.97 (0.96-0.98) | 0.98 (0.97-0.99) | 0.41 (0.32-0.49) | 0.99 (0.98-0.99) | 1.00 (1.00-1.00) | 0.87 (0.85-0.89) |
|  | 4 | 0.90 (0.85-0.93) | 0.96 (0.95-0.97) | 0.52 (0.48-0.57) | 0.96 (0.94-0.97) | 1.00 (1.00-1.00) | 0.87 (0.85-0.88) |
|  | 5 | 0.98 (0.97-0.99) | 0.99 (0.97-0.99) | 0.16 (0.03-0.27) | 0.99 (0.98-0.99) | 1.00 (1.00-1.00) | 0.82 (0.80-0.84) |
|  | 6 | 1.00 (1.00-1.00) | 0.99 (0.99-1.00) | 0.26 (0.16-0.34) | 0.97 (0.97-0.98) | 1.00 (0.98-1.00) | 0.84 (0.82-0.86) |
|  | mean | 0.96 (0.95-0.97) | 0.99 (0.98-0.99) | 0.45 (0.42-0.49) | 0.98 (0.97-0.98) | 1.00 (1.00-1.00) | 0.86 (0.85-0.87) |
|  |  |  |  |  |  |  |  |
| Maximum survival | 1 |  | 0.01 (-0.42-0.31) | 0.00 (-0.18-0.16) |  | 0.00 (-0.19-0.17) | 0.00 (-0.16-0.12) |
|  | 2 |  | 0.03 (-0.33-0.27) | 0.01 (-0.14-0.15) |  | 0.03 (-0.62-0.38) | 0.01 (-0.21-0.18) |
|  | 3 | 0.06 (-0.47-0.40) | 0.01 (-0.41-0.31) | 0.05 (-0.11-0.19) | 0.03 (-0.44-0.37) | 0.03 (-0.51-0.38) | 0.02 (-0.16-0.17) |
|  | 4 | 0.05 (-0.50-0.42) | 0.02 (-0.28-0.25) | 0.07 (-0.04-0.17) | 0.03 (-0.44-0.35) | 0.01 (-0.56-0.36) | 0.02 (-0.16-0.17) |
|  | 5 | 0.01 (-0.31-0.28) | 0.00 (-0.10-0.11) | 0.36 (0.25-0.45) | 0.01 (-0.34-0.25) | 0.01 (-0.64-0.39) | 0.07 (-0.07-0.18) |
|  | 6 | 0.00 (-0.16-0.15) | 0.00 (-0.05-0.05) | 0.26 (0.14-0.36) | 0.00 (-0.13-0.12) | 0.01 (-2.85-0.80) | 0.05 (-0.52-0.22) |
|  | mean | 0.03 (-0.19-0.18) | 0.01 (-0.13-0.11) | 0.12 (0.06-0.18) | 0.01 (-0.17-0.15) | -0.02 (-0.53-0.21) | 0.03 (-0.10-0.10) |

Figures between brackets are 95% credible intervals. Cells highlighted in green have credible intervals entirely above zero.

#### Table S20: Proportion of residual vectorial capacity prevented by improving LLIN durability properties in Zambia

|  |  | susceptible An. gambiae s.l. | | | resistant *An. gambiae* s.l. | | |
| --- | --- | --- | --- | --- | --- | --- | --- |
| Scenario | Semester | Olyset | PermaNet 2.0 | mean of all net brands | Olyset | PermaNet 2.0 | Mean of all LLIN brands |
| Intact | 2 | 0.70 (0.25-0.88) | 0.42 (-1.02-0.82) | 0.52 (-0.18-0.80) | 0.72 (0.51-0.85) | 0.52 (-0.16-0.82) | 0.61 (0.26-0.79) |
|  | 4 | 0.75 (0.25-0.91) | 0.75 (0.33-0.92) | 0.73 (0.44-0.87) | 0.73 (0.49-0.86) | 0.69 (0.38-0.86) | 0.70 (0.51-0.82) |
|  | mean | 0.71 (0.36-0.86) | 0.56 (-0.17-0.81) | 0.62 (0.23-0.79) | 0.72 (0.57-0.82) | 0.59 (0.24-0.80) | 0.65 (0.46-0.77) |
|  |  |  |  |  |  |  |  |
| Target content | 2 | -0.03 (-1.01-0.46) | 0.37 (-1.20-0.81) | 0.13 (-0.76-0.53) | -0.05 (-0.43-0.25) | 0.58 (0.03-0.83) | 0.26 (-0.08-0.45) |
|  | 4 | 0.09 (-0.87-0.56) | 0.79 (0.40-0.93) | 0.42 (-0.05-0.69) | 0.05 (-0.34-0.33) | 0.82 (0.65-0.91) | 0.43 (0.21-0.58) |
|  | mean | 0.00 (-0.61-0.38) | 0.57 (-0.21-0.82) | 0.26 (-0.24-0.52) | 0.00 (-0.27-0.22) | 0.70 (0.42-0.84) | 0.34 (0.14-0.47) |
|  |  |  |  |  |  |  |  |
| Maximum use | 2 | 0.80 (0.66-0.88) | 0.95 (0.87-0.98) | 0.87 (0.80-0.92) | 0.48 (0.36-0.60) | 0.76 (0.62-0.86) | 0.63 (0.52-0.70) |
|  | 4 | 0.77 (0.61-0.87) | 0.75 (0.53-0.86) | 0.75 (0.63-0.83) | 0.49 (0.34-0.61) | 0.55 (0.38-0.68) | 0.52 (0.40-0.60) |
|  | mean | 0.78 (0.68-0.85) | 0.85 (0.73-0.91) | 0.81 (0.74-0.86) | 0.49 (0.39-0.57) | 0.65 (0.54-0.74) | 0.57 (0.50-0.63) |
|  |  |  |  |  |  |  |  |
| Maximum survival | 2 | 0.05 (-0.96-0.52) | 0.03 (-2.04-0.69) | -0.03 (-1.11-0.46) | 0.00 (-0.36-0.27) | 0.00 (-1.04-0.49) | 0.00 (-0.55-0.29) |
|  | 4 | 0.02 (-0.98-0.53) | 0.03 (-1.00-0.52) | -0.03 (-0.66-0.39) | 0.00 (-0.40-0.28) | 0.02 (-0.49-0.33) | -0.01 (-0.32-0.23) |
|  | mean | 0.00 (-0.67-0.40) | -0.03 (-1.06-0.46) | -0.03 (-0.62-0.31) | -0.01 (-0.25-0.22) | -0.02 (-0.56-0.31) | -0.01 (-0.30-0.19) |

Figures between brackets are 95% credible intervals. Cells highlighted in green have credible intervals entirely above zero. Cells highlighted in purple have credible intervals that encompass zero, but were entirely above zero in the analysis assuming LLIN efficacy against resistant *An. gambiae* s.l.

## Figures illustrating the calculation of effects on vectorial capacity


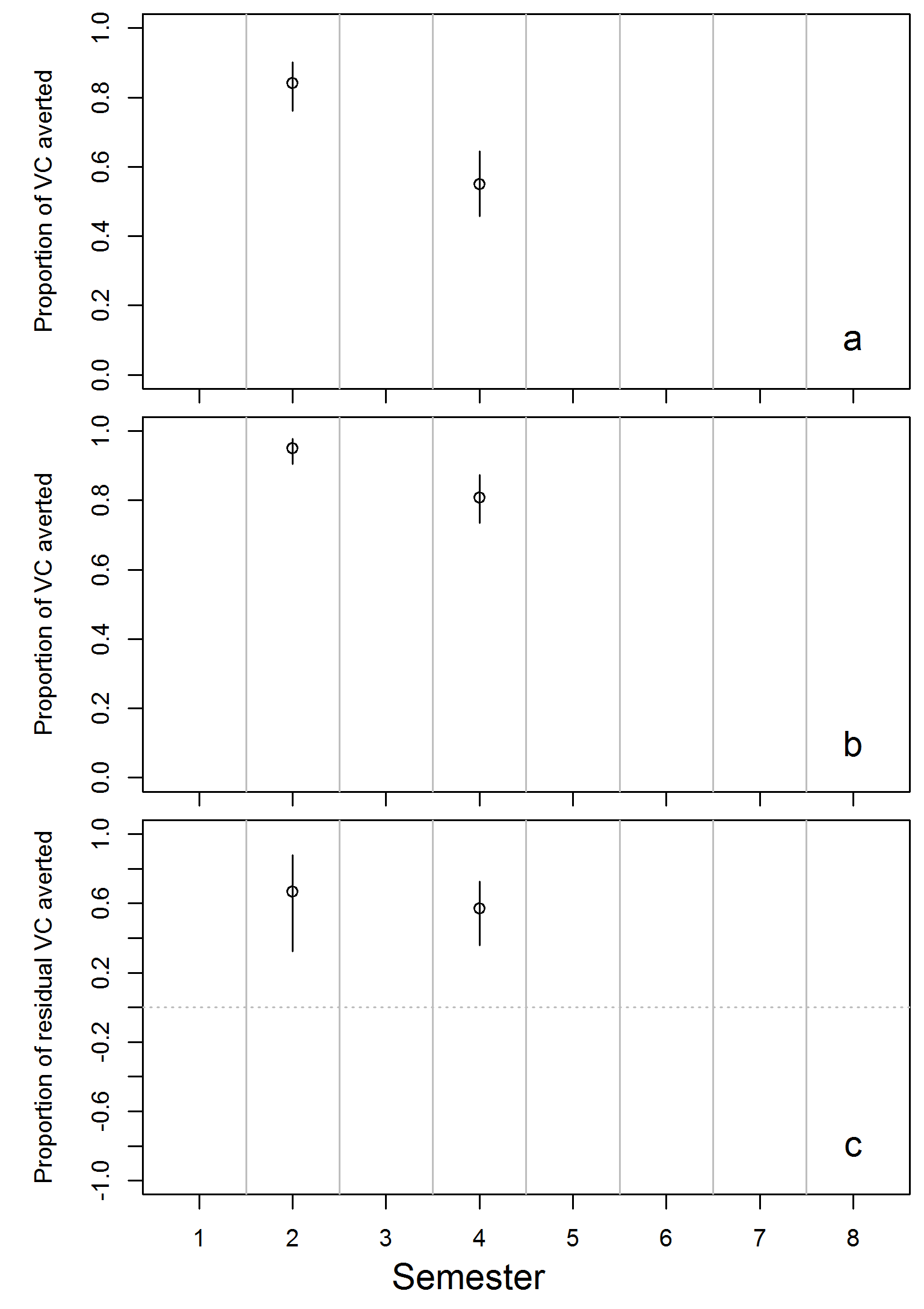


#### Figure S20. Illustration of the calculation of residual vectorial capacity (VC) averted in Angola when all LLINs are being used

**a** Proportion of VC averted by LLINs with observed durability properties, **b** Proportion of VC averted by LLINs when all LLINs are being used, **c** Proportion of residual VC that remains with LLINs with observed durability properties that is additionally averted by using all LLINs. Vertical bars show 95% credible intervals.

## Figures illustrating the potential overall effects of averting LLIN decay

#### Figure S21. Proportion of vectorial capacity of susceptible *An. gambiae* s.l. averted by LLINs by time since distribution

a Angola, b Benin, c Kenya, d Malawi, e Mozambique, f Rwanda (no data), g Senegal and h Zambia. Coloured vertical bars show 95% credible intervals.


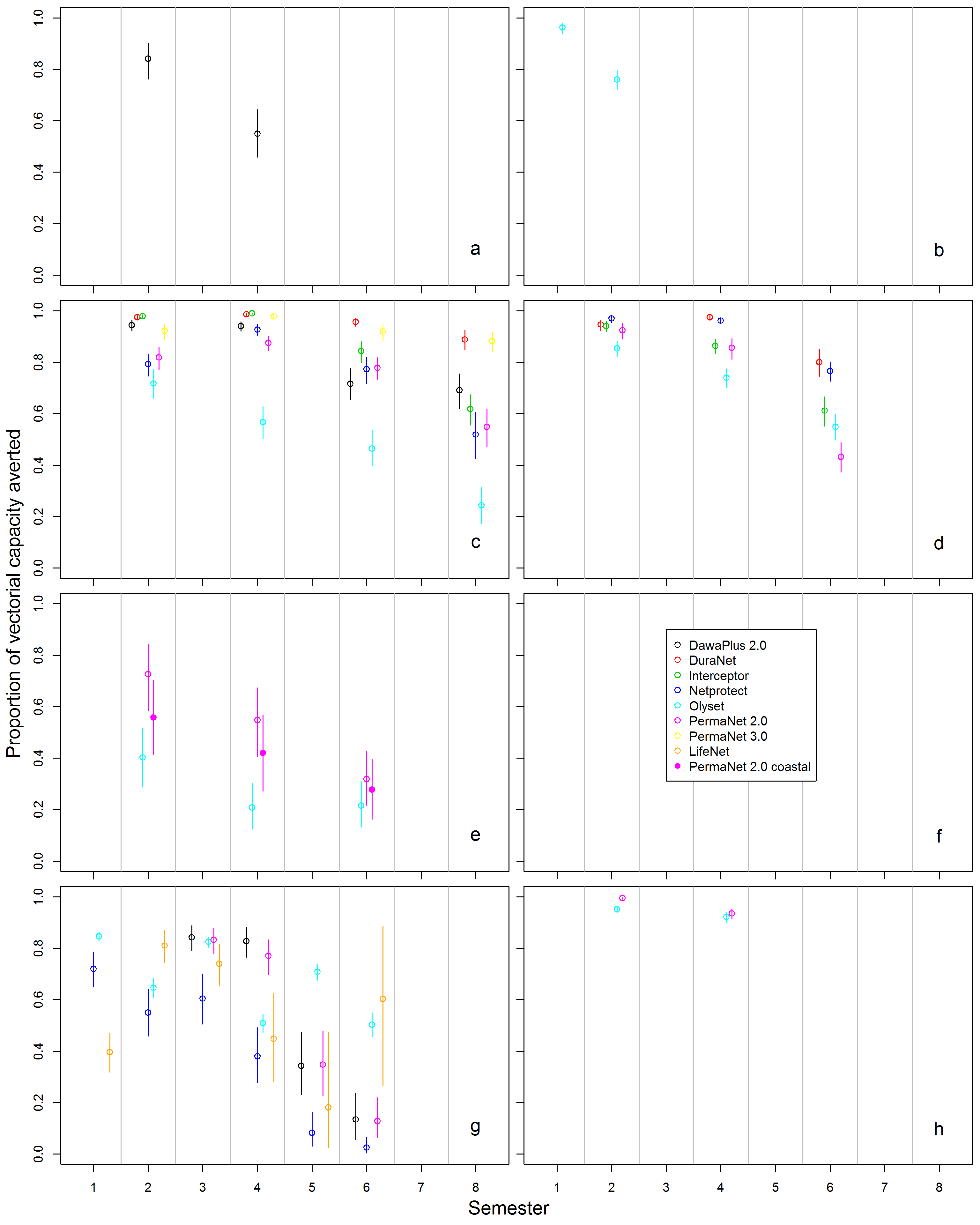


Figure S22. Proportion of vectorial capacity of resistant *An. gambiae* s.l. averted by LLINs by time since distribution

a Angola, b Benin, c Kenya, d Malawi, e Mozambique, f legend, g Senegal and h Zambia. Coloured vertical bars show 95% credible intervals.

## Figures illustrating the potential effects of averting specific components of LLIN decay


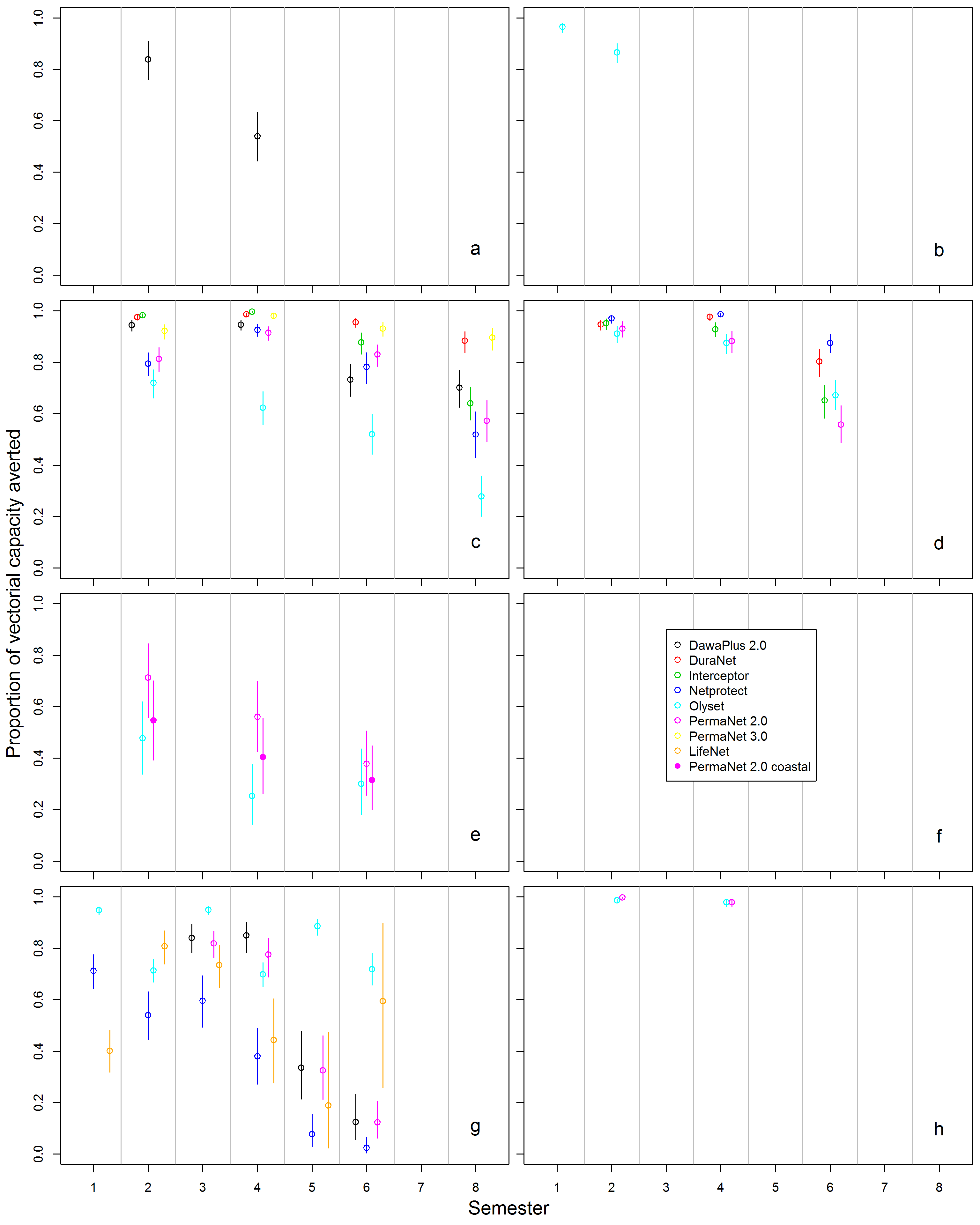


Figure S23. Proportion of vectorial capacity of resistant *An. gambiae* s.l. averted by LLINs in a scenario where all LLINs are intact

a Angola, b Benin, c Kenya, d Malawi, e Mozambique, f Rwanda (no data), g Senegal and h Zambia. Coloured vertical bars show 95% credible intervals.


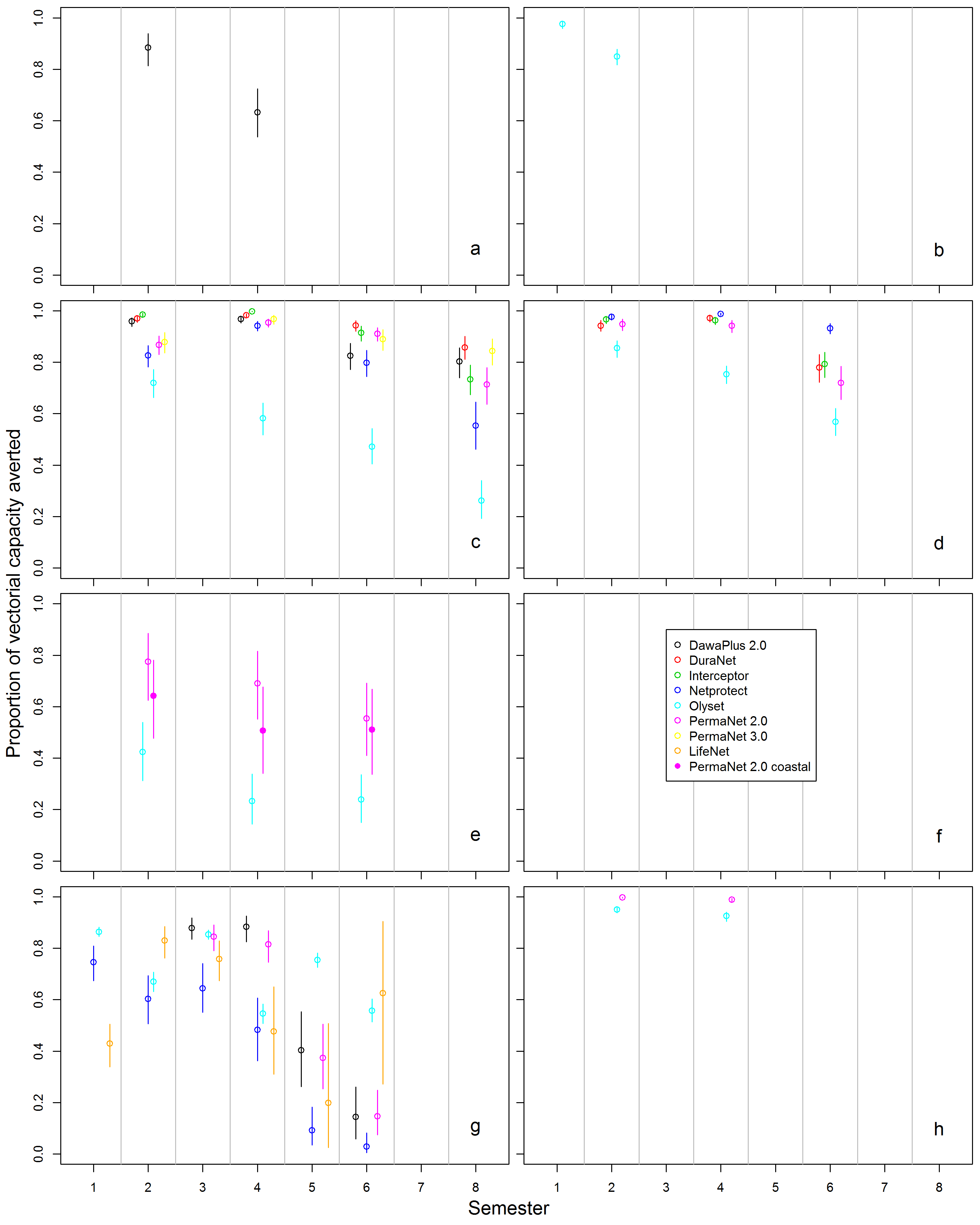


Figure S24. Proportion of vectorial capacity of resistant *An. gambiae* s.l. averted by LLINs in a scenario where all LLINs have the target content of insecticide

a Angola, b Benin, c Kenya, d Malawi, e Mozambique, f legend, g Senegal and h Zambia. Coloured vertical bars show 95% credible intervals.


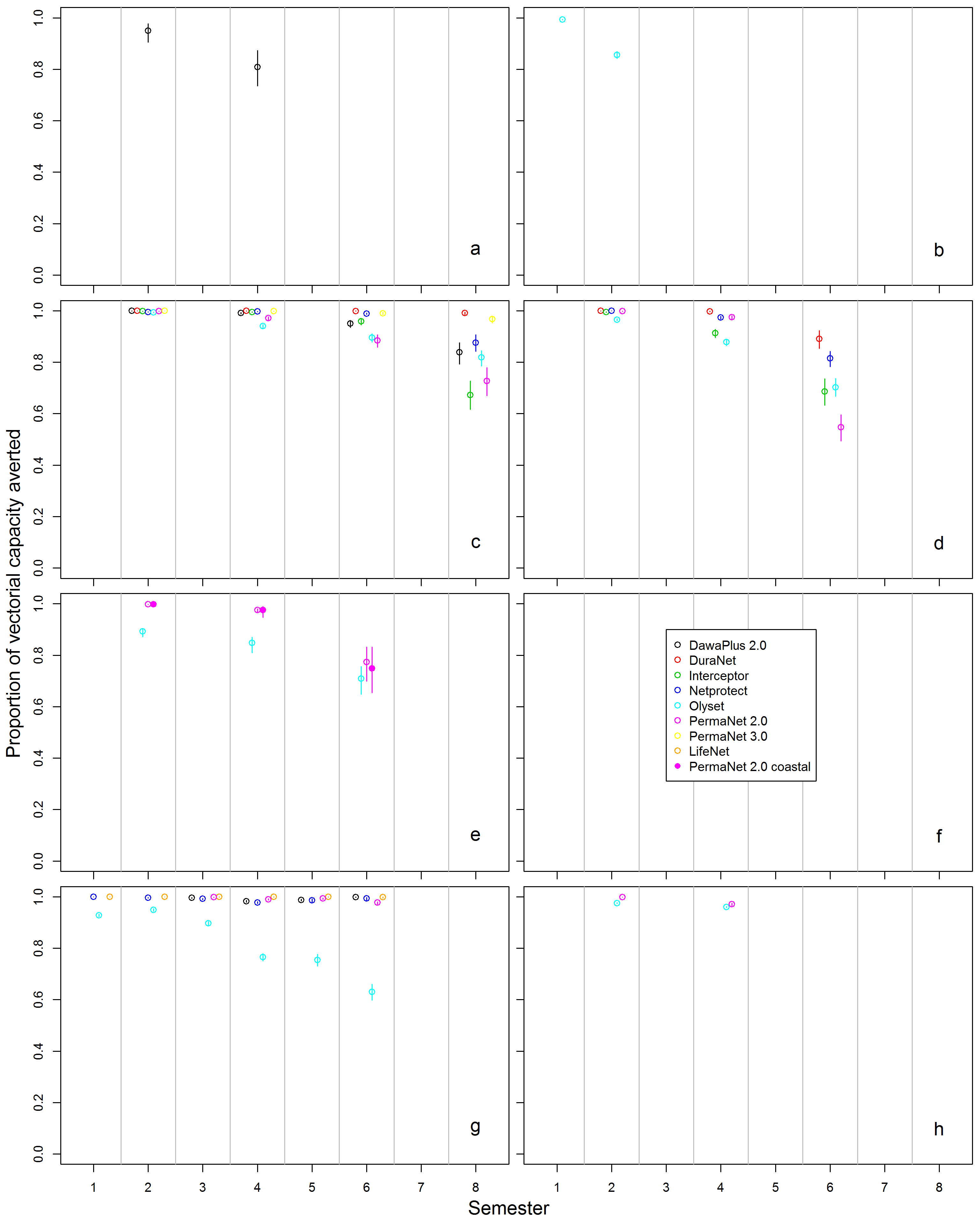


Figure S25. Proportion of vectorial capacity of resistant *An. gambiae* s.l. averted by LLINs in a scenario where all LLINs are being used

a Angola, b Benin, c Kenya, d Malawi, e Mozambique, f legend, g Senegal and h Zambia. Coloured vertical bars show 95% credible intervals.


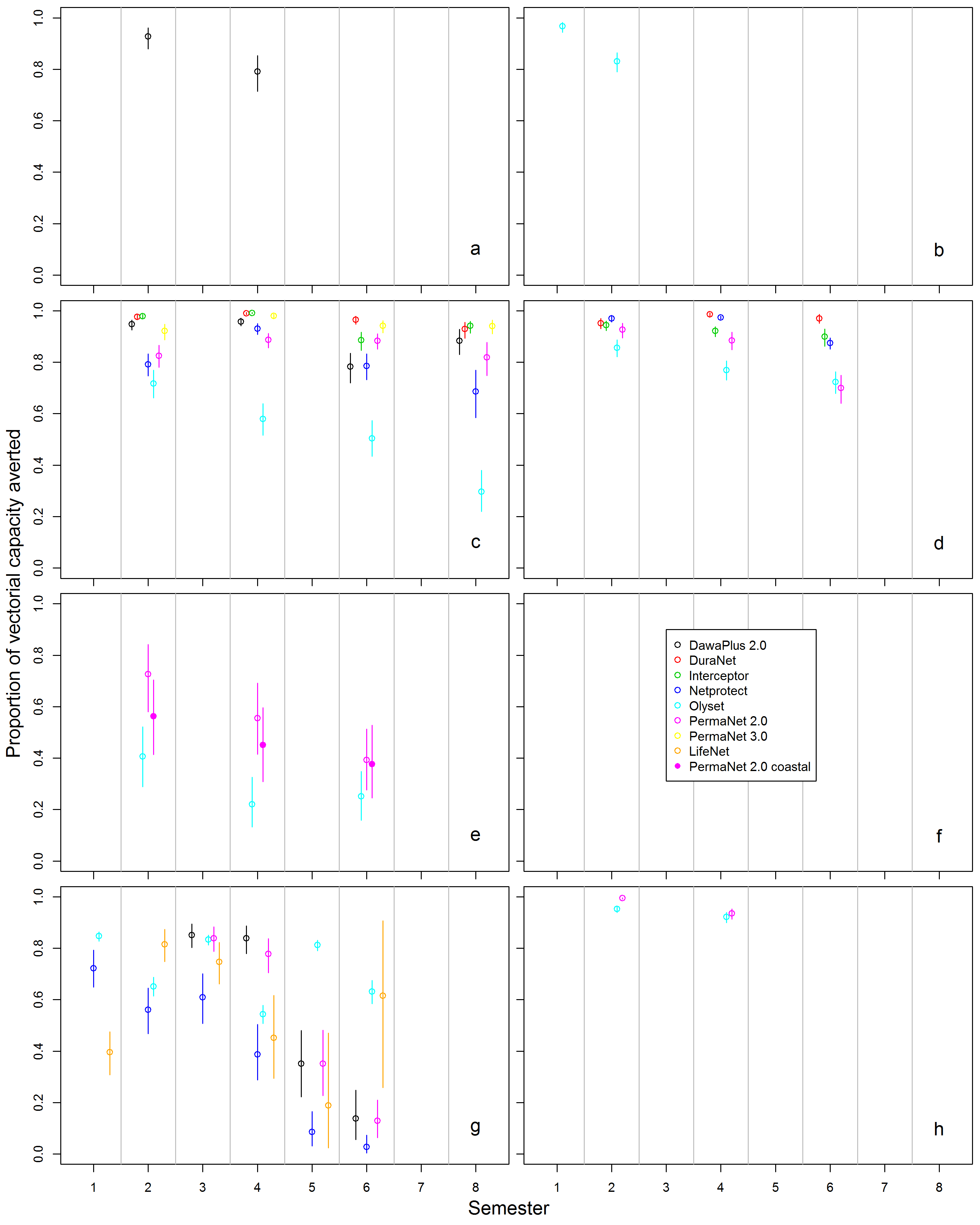


Figure S26. Proportion of vectorial capacity of resistant *An. gambiae* s.l. averted by LLINs in a scenario where LLINs do not attrite

a Angola, b Benin, c Kenya, d Malawi, e Mozambique, f legend, g Senegal and h Zambia. Coloured vertical bars show 95% credible intervals.


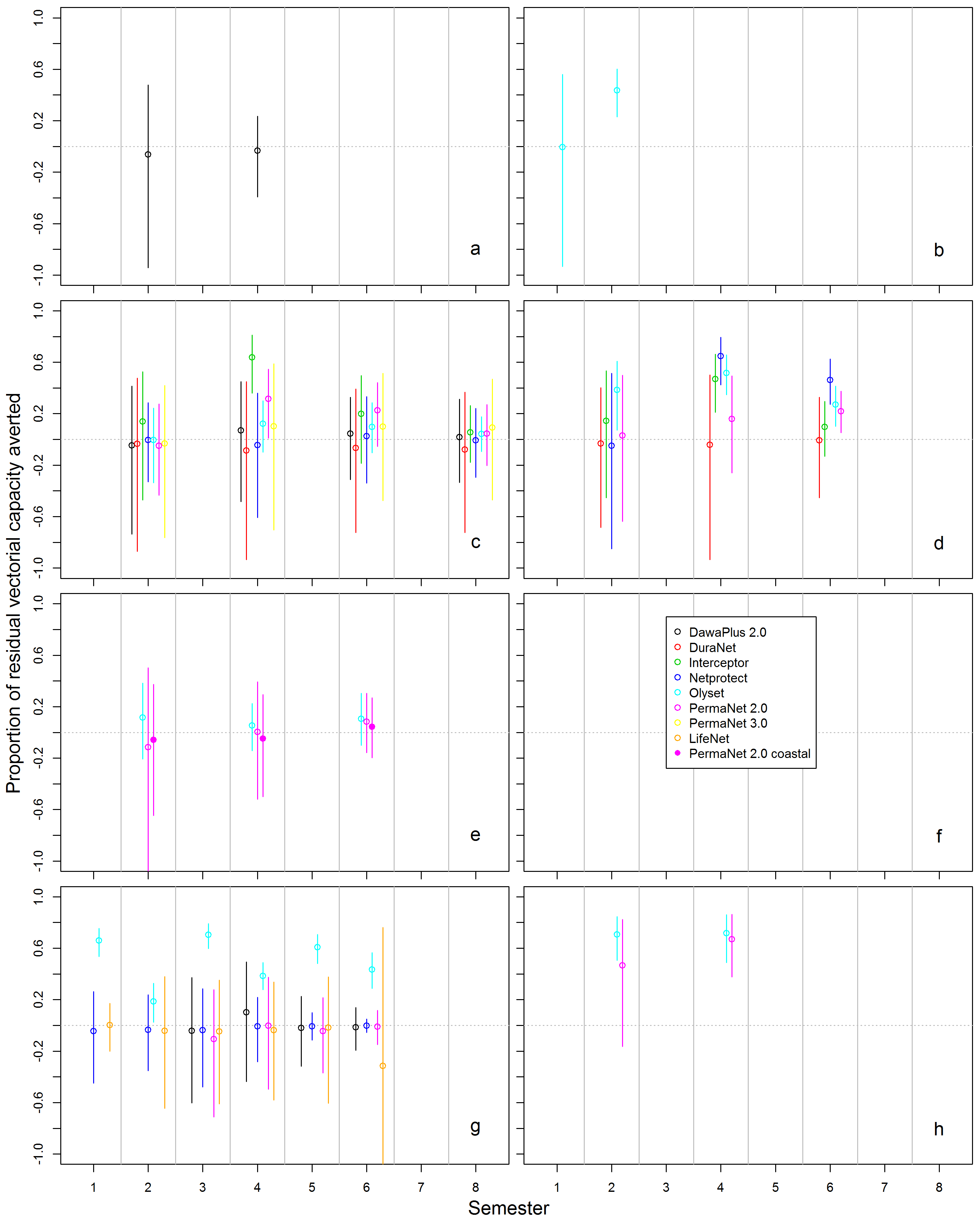


Figure S27. Proportion of residual vectorial capacity of resistant *An. gambiae* s.l. averted by LLINs in a scenario where all LLINs are intact instead of holed

a Angola, b Benin, c Kenya, d Malawi, e Mozambique, f Rwanda (no data), g Senegal and h Zambia. Coloured vertical bars show 95% credible intervals.


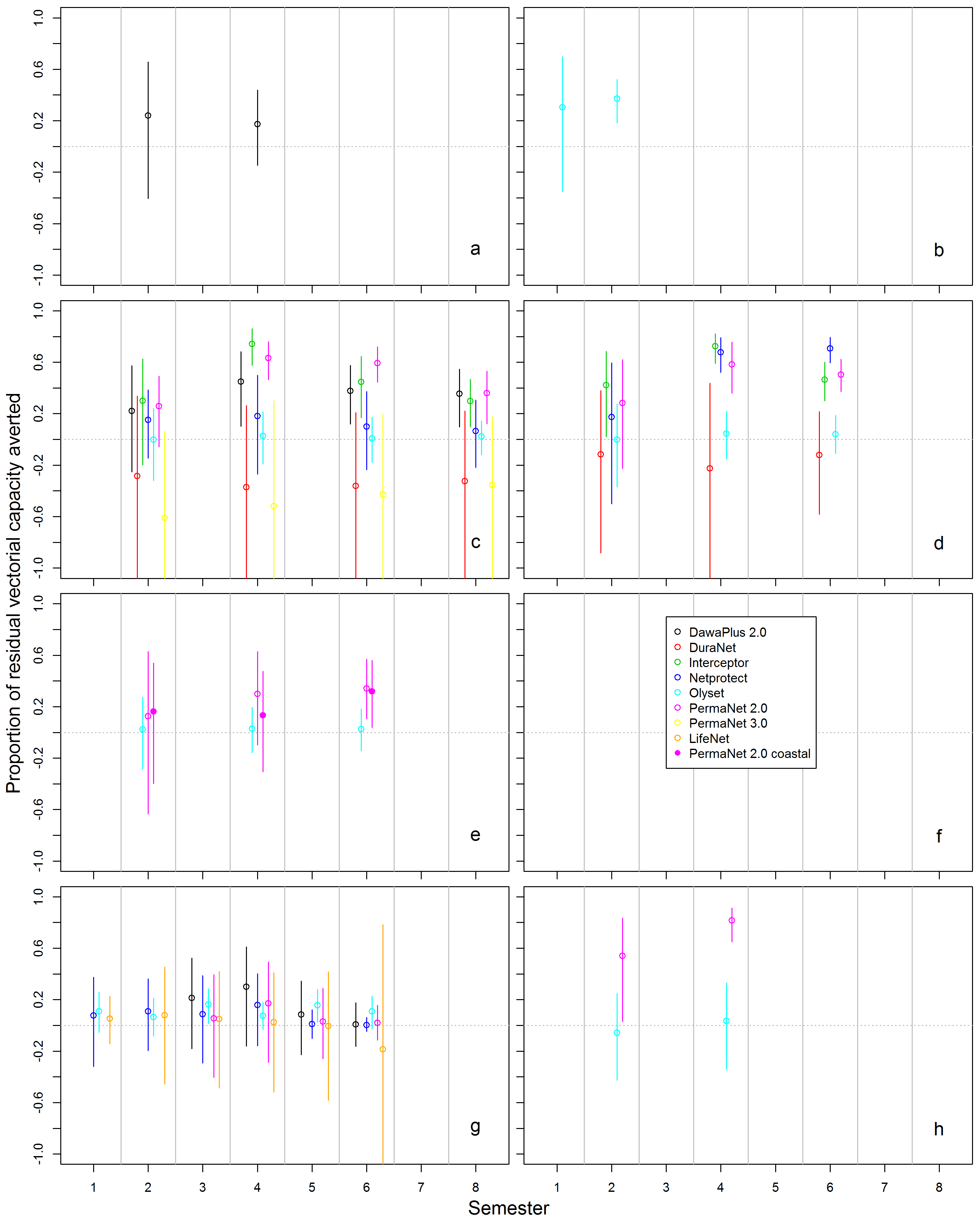


Figure S28. Proportion of residual vectorial capacity of resistant *An. gambiae* s.l. averted by LLINs in a scenario where all LLINs maintain their target insecticide content instead of a decayed content

a Angola, b Benin, c Kenya, d Malawi, e Mozambique, f legend, g Senegal and h Zambia. Coloured vertical bars show 95% credible intervals.

**
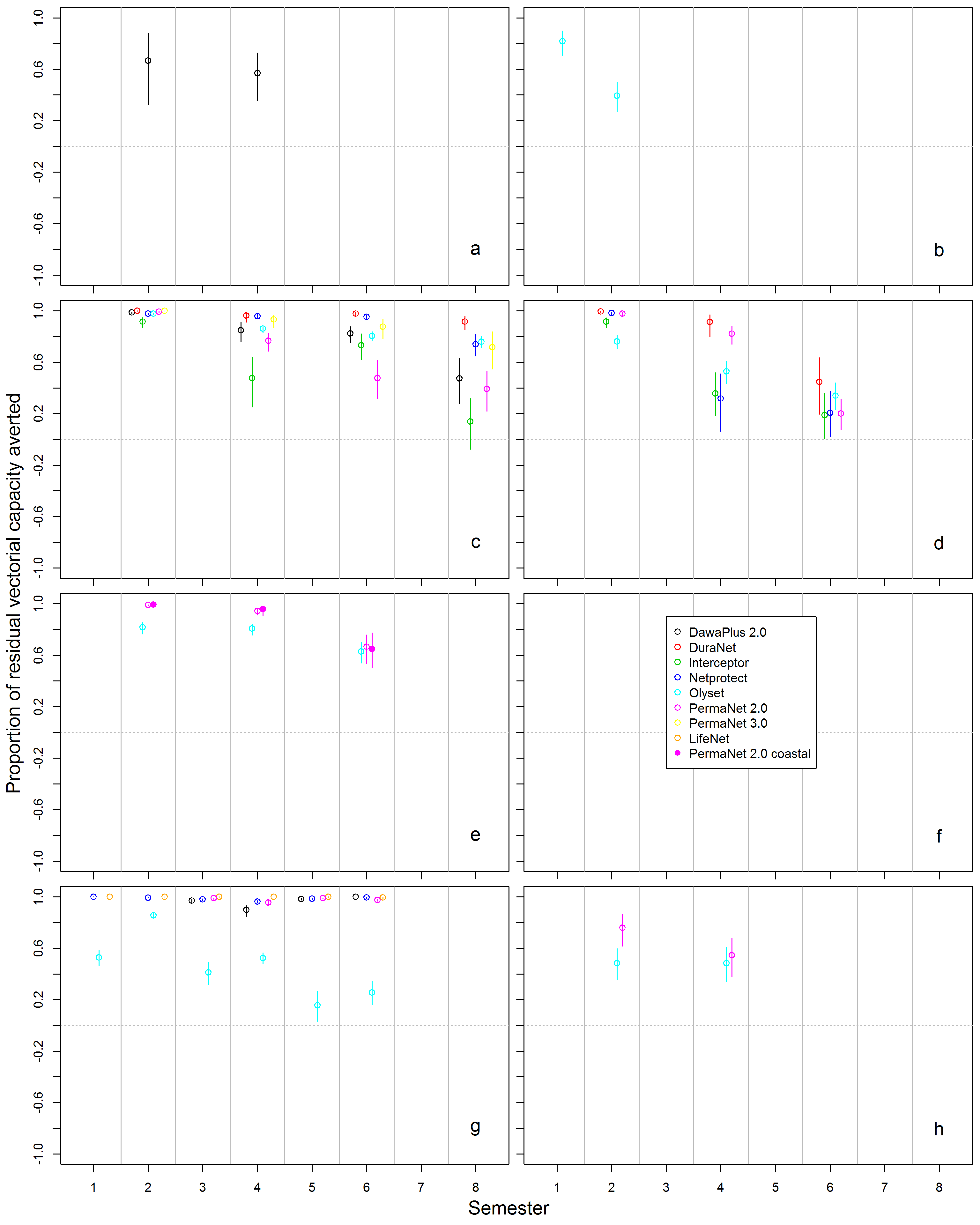
**

Figure S29. Proportion of residual vectorial capacity of resistant *An. gambiae* s.l. averted by LLINs in a scenario where all LLINs are used instead of measured use

a Angola, b Benin, c Kenya, d Malawi, e Mozambique, f legend, g Senegal and h Zambia. Coloured vertical bars show 95% credible intervals.


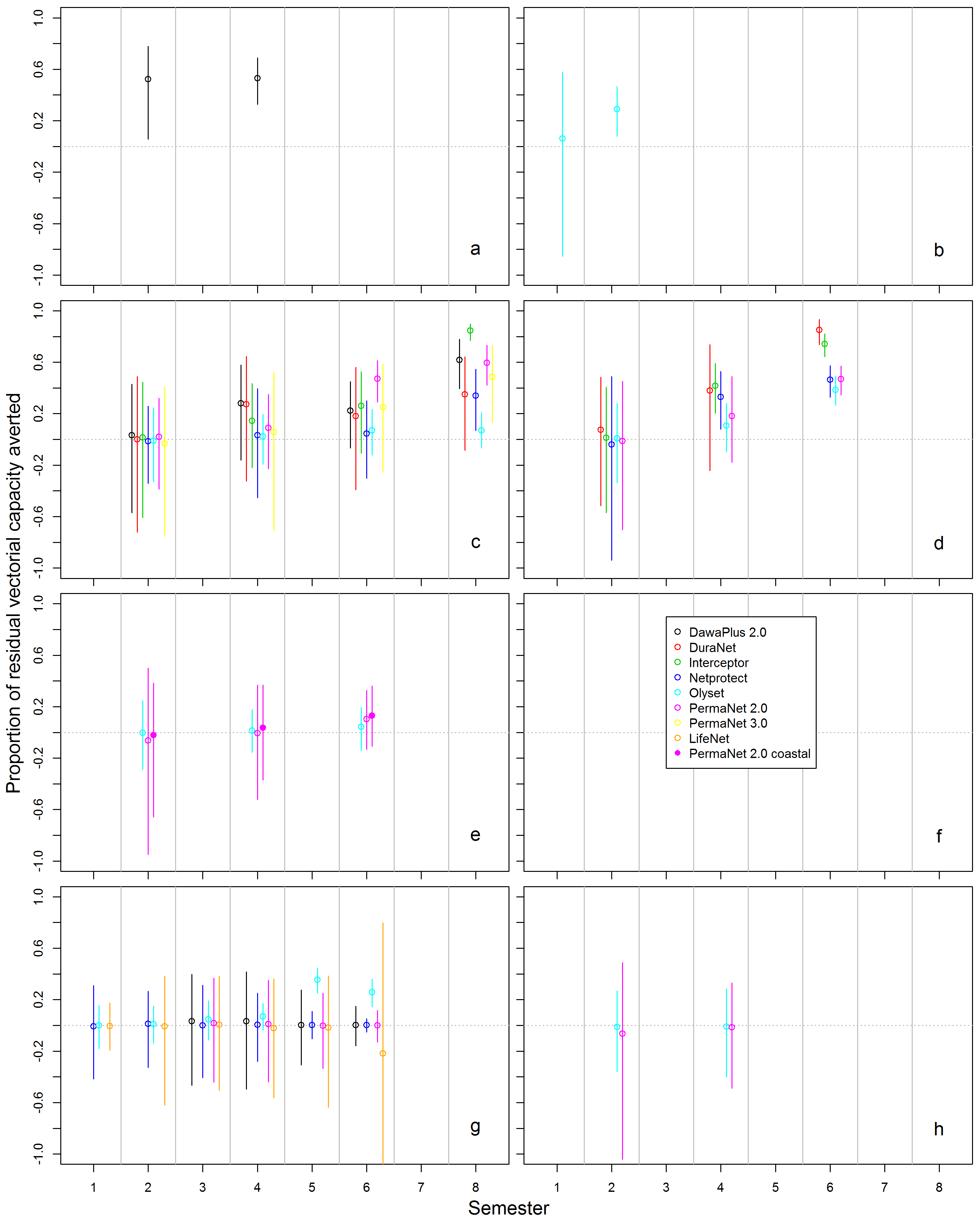


Figure S30. Proportion of residual vectorial capacity of resistant *An. gambiae* s.l. averted by LLINs in a scenario where LLINs do not attrite (instead of observed attrition in country studies)

a Angola, b Benin, c Kenya, d Malawi, e Mozambique, f legend, g Senegal and h Zambia. Coloured vertical bars show 95% credible intervals.

# References

1. Azondekon R, Gnanguenon V, Oke-Agbo F, Houevoessa S, Green M, Akogbeto M: **A tracking tool for long-lasting insecticidal (mosquito) net intervention following a 2011 national distribution in Benin.** *Parasites & Vectors* 2014, **7**.

2. R Core Development Team: *R: A language and environment for statistical computing.* Vienna, Austria 2006.

3. World Health Organisation GMP: **Test procedures for insecticide resistance monitoring in malaria vector mosquitoes.** 2nd edition. Geneva: World Health Organisation; 2016.

4. Briet OJ, Penny MA, Hardy D, Awolola TS, Van BW, Corbel V, Dabire RK, Etang J, Koudou BG, Tungu PK, Chitnis N: **Effects of pyrethroid resistance on the cost effectiveness of a mass distribution of long-lasting insecticidal nets: a modelling study.** *Malar J* 2013, **12:**77.

5. Randriamaherijaona S, Briet OJ, Boyer S, Bouraima A, N'Guessan R, Rogier C, Corbel V: **Do holes in long-lasting insecticidal nets compromise their efficacy against pyrethroid resistant Anopheles gambiae and Culex quinquefasciatus? Results from a release-recapture study in experimental huts.** *Malar J* 2015, **14:**332.

6. Briet OJ, Smith TA, Chitnis N: **Measurement of overall insecticidal effects in experimental hut trials.** *Parasit Vectors* 2012, **5:**256.

7. Chitnis N, Smith T, Steketee R: **A mathematical model for the dynamics of malaria in mosquitoes feeding on a heterogeneous host population.** *J Biol Dyn* 2008, **2:**259-285.

8. Briët OJTI, D.E., Chitnis, N.; Pothin, E.; Lemoine, J.F.; Frederic, J.; Smith, T.A.: **Models of effectiveness of interventions against malaria transmitted by Anopheles albimanus.** *Malar J* 2019, **18**.

9. Chitnis N, Schapira A, Smith T, Steketee R: **Comparing the Effectiveness of Malaria Vector-Control Interventions Through a Mathematical Model.** *American Journal of Tropical Medicine and Hygiene* 2010, **83:**230-240.

1. Country studies in Angola, Mozambique and Zambia collected information about the total number of nets in the household, and Zambia collected information on the number of other nets hanging. But since only households with study nets were in the active database, there is no information on what happened to net use after household dropped out (e.g. due to attrition of study-LLINs), and thus, about whether study-nets were effectively replaced by non-study nets. [↑](#footnote-ref-1)
