# Supplementary material for: Attrition, physical integrity and insecticidal activity of long-lasting insecticidal nets in sub-Saharan Africa and modelling of their impact on vectorial capacity
Source: Malar J. 2020 Aug 28;19:310. doi: 10.1186/s12936-020-03383-6 (PMC7456088; doi:10.1186/s12936-020-03383-6)
Supplement: Supplementary file 3 — Additional file 3: Ethics approval and consent to participate. [file 12936_2020_3383_MOESM3_ESM.docx]

**Additional file 3: Ethics approval and consent to participate**

Below are the ethical consideration sections of the study protocols and statements in publications:

### Angola

“Personal information collected on the questionnaire will only be used for follow-up purposes in locating house and the nets over the 3-year period. All data will be collected on locked PDAs and transferred to computers where only investigators will have access to.

Before interviewing and retrieving LLINs, surveyors will ask for permission of local leaders from that particular area. During follow up visits, a verbal consent text will be read in Portuguese or in the local language to the member of the household who receives the interviewer. A verbal consent text in shown in the survey questionnaire Appendix 2a and 2b.”

### Benin

Gnanguenon et al., 2014: Study clearance This prospective study was planned with and approved by the Ministry of Health. Community leaders were informed before the study and all gave verbal consent before initiation. Written consent was then obtained on the day of the study from all participating households.

### Kenya

#### Consent

During the initial survey of the study villages, informed consent will be obtained from the head of each household enrolled in study. Consent forms will be translated into the local language (DhoLuo) and back-translated into English to ensure accurate translation. The form will be read to each participant and, if they are unable to write, they will mark the form and a witness will sign indicating the study has been explained to the participant and s/he accepts to participate.

#### Risks to study participants

Risks to the study participants are minimal. The LLITN products we will distribute have been documented to cause minor side effects (itching/burning of the face, coughing, sneezing/runny nose) but these are usually temporary and fade after the first few days of use. Names will be retained for follow up purposes but all data will be collected on locked PDAs and transferred to computers where only the investigators and staff will have access. Paper forms will be used only as a backup to the PDAs if PDAs are unavailable or not working. If used, paper forms will be stored in a locked room in the entomology building at the Kisian campus. Access is restricted to the campus as well as the building where forms and data files will be stored.

#### Benefits to study participants

All participants will receive insecticide treated nets. Every 6 months over the next 5 years, 210 participants will receive replacement nets as their old nets are sampled for analysis. These nets have been demonstrated to reduce malaria morbidity and mortality in sub-Saharan Africa.”

### Malawi

The heads of all households enrolled in the study will be asked to sign a consent to participate in the study. The consent will be read to each person and a blank copy will be provided to all persons who agree to participate. A village representative will be asked to accompany study staff during the consenting process as literacy rates in this area are low. People who cannot write will be asked to provide a thumbprint and the form will be signed by the village representative as a witness. The use of a thumbprint and signature of a witness is a legally accepted practice in Malawi. The consent form will be translated into Chichewa, the primary language in this area and back-translated into English to ensure accuracy. All persons living in study villages will be eligible to participate in this study. Each product to be tested in the current study has been evaluated by WHOPES for safety and are considered safe for routine use (WHOPES, 2001, WHOPES, 2004, WHOPES, 2007). In addition, the insecticides used in these products and the nets have been registered and approved for use by the Pesticides Control Board (PCB) based at Bvumbwe Agricultural Research Station in Thyolo, Malawi. The insecticides on the nets may cause symptoms such as itching/burning of the face, coughing, or sneezing/runny nose. Rarely, people experience headaches or nausea. These problems usually go away a few days after you start using your net. All nets will be shipped directly from the manufacturers to Blantyre where they will be kept within individually wrapped packages. The individual net packages will be opened when persons enroll in the study and the nets are supplied to their houses.

Names of the heads of households and geographic location of households will be maintained for tracking purposes only. No data on individual persons will be collected. Names of heads of households and the geographic location of the households will be maintained on computer files and PDAs which are accessible only to study staff. Computer files and PDAs will be stored in locked areas and will be password protected.

Participants will likely benefit from this study. We will provide insecticide treated nets which have been shown to protect against severe disease and death due to malaria.

### Mozambique

VandenEng et al., 2015: Written consent was obtained by all study subjects or by their parent or guardian if they were under 16 years old. Participants signed or provided a thumbprint on two copies of the consent form, one of which was kept by the investigators and the other was given to the participants. Approval for this study and consent procedure was obtained from the Bioethical Committee of the Ministry of Health, Mozambique (CNBS, Maputo, Mozambique). CDC investigators participated under a non-research determination on the basis of routine public health evaluation.

Morgan et al, 2014: Approval for this study was obtained from the Bioethical Committee of the Ministry of Health, Mozambique (Maputo, Mozambique). Centers for Disease Control and Prevention (CDC) Institutional Review Board (IRB) approved the study as a routine public health evaluation that did not require CDC IRB review.

Study protocol: LLINs are an important part of the Mozambique’s national policy for malaria control. The 2007 national ITN distribution policy focuses on universal access to LLINs. This proposal is to monitor the performance of the LLINs distributed in this campaign as part of a public health program activity. Initials of the head of households in survey questionnaire will only be used for follow-purposes. All data will be collected on locked PDAs and transferred to computers where only investigators and staff will have access. Before interviewing and retrieving LLINs, surveyors will ask for permission of local leaders from that particular area. During the follow up visit, a verbal consent text will be read in Portuguese to the member of the household who receives the interviewer. The verbal consent text in shown in the survey questionnaire Appendix 1.

### Senegal

The protocol contains a “Fiche de consentement”.

### Zambia

The protocol for this study(Tan et al., 2016) was approved by investigational review boards at the US Centers for Disease Control and Prevention and the Tropical Disease Research Centre in Zambia. Written consent was obtained, using a consent form that had been translated to the local language, from an adult over the age of 18 years old at participating households.

#### Consent

All homes in each village will have an equal chance of being selected to participate as long as they have an LLIN from the 2011 mass distributions that is hung up. Informed consent will be obtained from all participants in Bemba or English, depending on participant preference. The one adult in each home who agrees to participate will sign the consent form. The consent form will only be signed during the first visit to enroll the home. For participants who may not be able to read, the consent will be read to them. A signed consent form, or thumbprint for those who cannot write, will be kept by the study coordinator. Households that refuse to participate will not be included in the study but this will not impact their interaction with other Peace Corps initiatives in their village.

#### Confidentiality

Houses will be assigned a study ID number. A house occupant’s first name and ID number will be recorded and kept by the partner. After analysis of the study data, lists linking the house occupants name to the study ID number will be destroyed.

#### Adverse Events

Risks to the participant are minimal and include answering personal questions about LLIN use.

TAN, K. R., COLEMAN, J., SMITH, B., HAMAINZA, B., KATEBE-SAKALA, C., KEAN, C., KOWAL, A., VANDEN ENG, J., PARRIS, T. K., MAPP, C. T., SMITH, S. C., WIRTZ, R., KAMULIWO, M. & CRAIG, A. S. 2016. A longitudinal study of the durability of long-lasting insecticidal nets in Zambia. *Malar J,* 15**,** 106.

WHOPES. 2001. Review of Olyset Net and Bifenthrin 10% WP. Report of the 5th WHOPES Working Group Meeting, 2001.

WHOPES. 2004. Review of Vectobac WG, PermaNet and Gokilaht-S 5EC. Report of the 7th WHOPES Working Group Meeting, 2004.

WHOPES. 2008. Review of Spinosad 0.5% GR and 12% SC, Lambda-Cyhalothrin 10% CS, K-O TAB 1-2-3®, Interceptor®. Report of the 10th WHOPES Working Group meeting, 2007.
